# ‘Multi-cropping’, Intercropping and Adaptation to Variable Environments in Indus South Asia

**DOI:** 10.1007/s10963-017-9101-z

**Published:** 2017-05-09

**Authors:** C. A. Petrie, J. Bates

**Affiliations:** 1 Division of Archaeology, University of Cambridge, Downing Street, Cambridge, CB2 3DZ UK; 2 Selwyn College, University of Cambridge, Cambridge, CB3 9DQ UK; 3 McDonald Institute for Archaeological Research, University of Cambridge, Downing Street, Cambridge, CB2 3ER UK

**Keywords:** Cropping strategies, ‘Multi-cropping’, Environmental diversity, Adaptation, Resilience, Indus Civilisation, South Asia

## Abstract

Past human populations are known to have managed crops in a range of ways. Various methods can be used, singly or in conjunction, to reconstruct these strategies, a process which lends itself to the exploration of socio-economic and political themes. This paper endeavours to unpack the concept of ‘multi-cropping’ by considering diversity and variation in the cropping practices of the populations of South Asia’s Indus Civilisation. It argues that nuanced interpretations of the evidence provided by the combinations of crop seeds and weeds present in specific contexts and phases of occupation can reveal much about Indus cropping strategies, which in turn enables consideration of issues related to adaptation, intensification and resilience in the face of changing social, political, economic and environmental climates.

**Electronic supplementary material:**

The online version of this article (doi:10.1007/s10963-017-9101-z) contains supplementary material, which is available to authorized users.

## Introduction

**Fig. 1:**
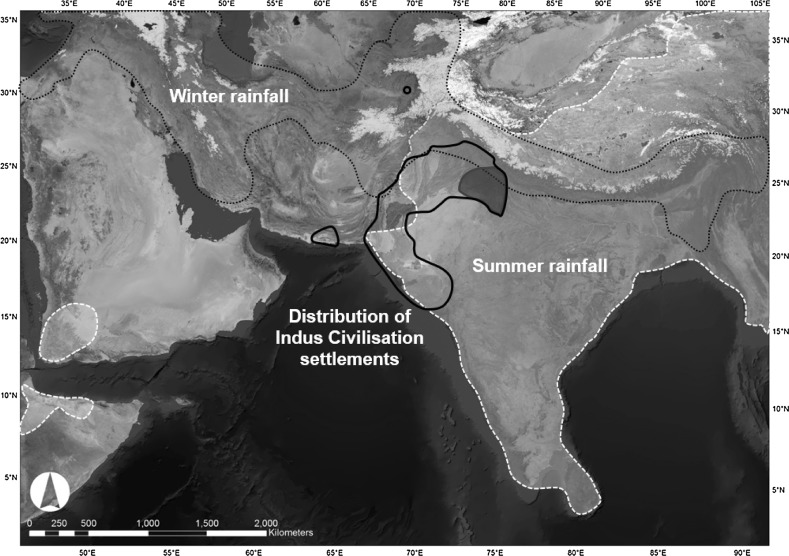
Distribution of Indus Civilisation settlements in relation to the environmental and climatic context of northwest South Asia

There is a growing body of literature devoted to investigating how human populations manage crops, with themes including the socio-economics of intensification, extensification, diversification, water supply, land ownership and labour organisation (e.g. Halstead 1992; Morrison 1994; Bogaard 2005; Marcus and Stannish [Eds.] 2006; Bogaard et al. 2013; Morehart and De Lucia [Eds.] 2015). The quotidian practices of sowing, tillage, rotation, fallow, weeding and watering provide fundamental insights into crop management, but it is challenging to resolve them archaeologically. These aspects have been investigated to varying degrees of resolution in regions that receive rainfall in specific seasons: for instance in places like Europe and the ancient Near East (e.g. Jones et al. 1999; Bogaard et al. 1999, 2001, 2011, 2015; Bogaard 2005, 2015; Halstead 2014; Styring et al. 2016), which are dominated by winter rainfall; and in Mesoamerica (e.g. Ford and Nigh 2010), sub-Saharan Africa (e.g. Stone et al. 1990; Stump 2013), parts of East Asia (e.g. Fuller and Qin 2009; Weisskopf et al. 2014, 2015), and south and eastern India (Morrison 1992; Kingwell-Banham et al. 2012; Weisskopf et al. 2014, 2015), which are dominated by summer rainfall. In these instances, where rainfall primarily comes in the one season, crop species suited to those water availability regimes were exploited, and farming activities were concentrated in particular times of the year. In comparison, the agricultural strategies utilised in the challenging climatic and environmental conditions that prevail in other parts of the world, where both winter and summer rainfall systems operate, have the potential to be multi-seasonal and thus more complex in terms of scheduling and management. They are, however, less studied and thus less well understood. The South Asian subcontinent stands out as a region characterised by a number of distinctive forms of early farming, including the exploitation of winter and summer cereals, pulses and fruits (Fuller 2011; Kingwell-Banham et al. 2015), the cultivation of which was enabled (and constrained) by the high level of environmental diversity. The populations of South Asia’s Indus Civilisation, who occupied areas of modern Pakistan and India, made use of a range of these crops and managed to occupy, and thrive in, a zone that straddled an important environmental threshold, where winter and summer rainfall systems overlapped (Fig. 1; Petrie et al. 2016, 2017; Petrie 2017).

The need to unravel the complexities of Indus cropping strategies has long been regarded as a fundamental challenge for South Asian archaeobotany (e.g. Fairservis 1967; Vishnu-Mittre and Savithri 1982a, b; Fuller and Madella 2002; Madella and Fuller 2006; Weber et al. 2010). Descriptions of Indus cropping practices often use terms like *mixed*-*cropping*, *double*-*cropping* and *multi*-*cropping* to characterise a range of strategies for growing multiple crops in one or more seasons (e.g. Vishnu-Mittre and Savithri 1982a, b; Chakrabarti 1988; Butler 1999; Weber 1999, 2003; Fuller and Madella 2002; Wright 2010). Such terms have implications for understanding the intensification of crop production, the degree to which diversification was important, and the impact of those factors on the sustainability of farming practices. While cropping in more than one season has certainly been discussed, and coherent models for diversity in practices have been developed (e.g. Weber 1999, 2003), the nuances of farming as practised on the ground have not typically been addressed. It is arguable that there has also been a tendency to apply the terms *multi*-*cropping* and *intensification* to archaeobotanical assemblages uncritically, and that the nuances of cropping multiple species with a range of environmental requirements during one season and the rotation of these species across seasons have not been explored in sufficient detail.

South Asia’s Indus Civilisation is an ideal laboratory for revisiting the way in which cropping practices and strategies are characterised, and examining how they can be identified archaeologically. This paper reviews the concepts that underpin our understanding of cropping strategies and explores the nature and distribution of the extant data that can be used in discussing the cropping and multi-cropping strategies of different Indus Civilisation populations. It also highlights the ways in which Indus cropping strategies were adapted to different environments. To do this, it will (a) unpack the term *multi*-*cropping*; (b) assess how multi-cropping and diversification have been identified archaeobotanically; (c) review prevailing paradigms about Indus subsistence; and (d) interrogate the archaeobotanical evidence for crop exploitation at Indus settlements in different environmental zones. The final section includes new data from northwest India, which illustrates the diverse ways that village farmers engaged in complex food production strategies involving both winter and summer crops before, during and after the existence of the first cities in the subcontinent. These data decisively advance our understanding of intra- and inter-regional diversity in practices, and facilitate more nuanced discussions of Indus cropping more broadly.

There should be no doubt that worldwide, early farming practices were diverse, and current evidence suggests that the ways in which particular crops were grown was variable (see Barker and Goucher 2015). Arguably, bringing precision to the description of cropping systems and ascertaining the degree to which particular practices were widespread or distinctive is essential for properly characterising the specific practices of different farming populations in the past. The overview presented here aims to explore how more complex definitions of cropping impact our understanding of Indus agricultural strategies, and provide a framework for the identification and discussion of cropping choices elsewhere.

## What is ‘Multi-cropping’?

*Multi*-*cropping* is a term developed by agronomists that can refer both to growing crops in multiple seasons and to growing more than one crop in a single season (Gallaher 2009, p. 255; see Andrews and Kassam 1976; Francis 1986). Gallaher (2009, p. 255) has clarified the definition by arguing that multi-cropping is ‘the production of two or more crops per year on the same land’. Multi-cropping is thus distinct from *mono*-*cropping* and *monoculture*, where one crop is grown on the same plot for one or more years respectively (Andrews and Kassam 1976; Francis 1986; Butler 1999, Table 24.1).

The principle patterns of multi- (or multiple) cropping were first defined by Andrews and Kassam (1976, Table 1; also Francis 1986; see Butler 1999), who divided it into two forms: *sequential multi*-*cropping* and *intercropping*. The first refers to the growing of crops in sequence on the same area of land in the space of one year, during which ‘the succeeding crop is planted after the preceding crop has been harvested’ (Andrews and Kassam 1976, p. 2). Farmers thus manage one crop in one parcel of land at one time. Andrews and Kassam (1976, Table 1) further subdivided sequential multi-cropping as follows:

- *Double cropping*: growing two crops in sequence
- *Triple/quadruple (etc.) cropping*: growing three/four (+) crops in sequence and
- *Ratoon cropping*: cultivation of regrowth from stubble/roots following initial harvest.

Gallaher (2009, p. 257) has added a number of extra definitions:

- *Mono*-*culture*: the same crop grown in succession (e.g. wheat followed by wheat)
- *Duo*-*culture*: successions of the same types of crops (e.g. grain followed by grain; pulse followed by pulse; fodder followed by fodder)
- *Poly*-*culture*: combinations of different types of crops grown for different purposes (e.g. grain followed by fodder).

It is also possible to grow multiple crops simultaneously on the same land, which is referred to as *intercropping* (Andrews and Kassam 1976, p. 2). As with sequential multi-cropping, Andrews and Kassam (1976, p. 2) suggested that intercropping can take several forms:

- *Mixed intercropping*: growing two or more crops at the same time with no distinct rows or divisions
- *Row intercropping*: growing two or more crops at the same time in rows
- *Strip intercropping*: growing two or more crops at the same time in strips wide enough apart to allow independent cultivation
- *Relay intercropping*: growing two or more crops at the same time for part of their life cycles (i.e. planting the second before the first has been harvested).

Andrews and Kassam (1976, p. 3) also noted that sequential multi-cropping should be distinguished from *mixed farming*, which they defined as ‘cropping systems which involve the raising of crops, animals and/or trees’; and *rotation* systems, in which there is ‘a repetitive cultivation of an ordered succession of crops (or crops and fallow) on the same land’, where a cycle of crop growth takes several years. Importantly, sequential multi-cropping and intercropping are not mutually exclusive, and can both be practised as part of one overarching strategy, provided due attention is paid to soil fertility, growth habits and the like. Although these categories and definitions provide a coherent framework for attempting to delineate different cropping strategies, discussion of the complexities of ‘multi-cropping’ agricultural strategies within archaeology has been relatively limited.

## How are Cropping and ‘Multi-cropping’ Identified Archaeologically?

Archaeology rarely provides direct evidence for cropping practices in the past. Occasionally fields have been identified (e.g. Hall 1982, 1988; Lal 2003, pp. 95–98), but the exposure of such features is not typically the objective of excavation. Analysis of plant remains can identify the crop and associated plant species found at excavated sites, but the plant remains that survive in the archaeological record are typically either discarded residues or material that has been preserved accidentally through waterlogging, desiccation, mineralisation or carbonisation (Dincauze 2000, pp. 332–343). Importantly, the material that is recovered from ‘closed’ or ‘open’ contexts within an archaeological settlement (e.g. pit fills, floor surfaces, collapse debris), does not necessarily provide a direct analogue for cultivation practices in the fields. Furthermore, human actions, such as the post-cultivation mixing of crops, can obscure the evidence for actual cropping practices before deposition, and post-depositional mixing can obscure things further. Nonetheless, several attempts have been made to differentiate cropping strategies archaeologically, particularly in parts of Europe, the ancient Near East and Mesoamerica.

In Europe and the Mediterranean there have been attempts to identify *maslin* cropping, where two crops are mixed for sowing. Amongst other things, maslin cropping is a form of risk buffering (Marston 2011; see Halstead and O’Shea 1989), and has been described ethnographically and archaeobotanically (e.g. Halstead and Jones 1989; Jones and Halstead 1995). The medieval western European strategy of sowing wheat and rye together is a maslin system (van der Veen 1995) and fits the definition of mixed intercropping given above. Several methods have been proposed to identify mixed intercropping archaeobotanically, including comparing the relative proportion of grain types and analysing the weed assemblages in relation to the weed suites expected of different crops (van der Veen 1995). However, factors such as soil conditions may affect decisions about crop mixtures, and definitive archaeological characterisation is hampered by several factors, including the challenge of interpreting a) variation in the proportions of the specific crops present, and b) behaviour that sees crops combined in the same pits after harvest (Jones and Halstead 1995). In their ethnographic study on the Greek island of Amorgos, Jones and Halstead (1995) noted that sown proportions of up to 80% wheat and 20% barley were considered mixed intercrops by farmers, but observed that changing conditions over the year may lead to one crop being more successful, thus changing the proportions in a harvested crop (Jones and Halstead 1995; van der Veen 1995). They also highlighted issues of contamination resulting from imperfect isolation of crops from previous growing cycles (Jones and Halstead 1995). Unfortunately, archaeological data is not typically able to reveal such nuances, and van der Veen (1995) has argued that careful statistical analysis, supported by proximate ethnographic analysis, is likely the only way to distinguish archaeologically between mono-crop and mixed intercropping strategies (e.g. Jones and Halstead 1995; see Marston 2011). It is notable that isotopic studies of samples of wheat and barley from a storage deposit at Vaihingen in Germany have identified examples of both species that shared distinctively low δ^13^C signatures relative to other samples, which suggests that they may have been grown together (Fraser et al. 2013).

Different problems beset the archaeobotanical interpretation of the *milpa* agricultural strategy, the distinctive Mesoamerican approach to farming involving the ‘three sisters’: maize (*Zea mays*), beans (*Phaseolus vulgaris* L.) and squash (*Cucurbita* sp.) (Emerson 1953; Ford and Nigh 2009, 2010). These three crops are grown simultaneously in companion planting where each of the species is interdependent. Milpa systems are therefore a specific form of mixed or even relay inter-cropping and often involve rotation over multiple years (Ford and Nigh 2009, 2010; Postma and Lynch 2012; see also Kennet et al. 2012).

Recent studies of weed ecologies and stable isotope ratios of crop grains from the eastern Mediterranean and western Asia have suggested that within an overarching arid to semi-arid environment where irrigation was being practised, wheat and barley were frequently grown in locations that were wetter and drier respectively (Wallace et al. 2015, p. 12). The implication is that wheat received greater levels of water and/or irrigation than barley, either in terms of the quantity of water or the number of irrigations (Wallace et al. 2015, p. 13). In contrast, pulses appear to have been grown in a range of watering conditions, suggesting that their watering was opportunistic (Wallace et al. 2015, p. 14). Although the growing of multiple crops was common across a broad area of the eastern Mediterranean and western Asia, these results confirm that early farmers were extremely cognisant of the water requirements of individual crops, and suggest that crops were consciously sown in specific locations, often in separate fields, where the supply of water could be managed to maximise yield. This system is essentially a form of mono-culture and/or mono-cropping, though it could also have involved the intercropping of pulses (see Butler 1999, Table 24.1).

In contrast to these examples of mono-cropping, or inter-cropping to grow more than one crop in one season, South Asia—and particularly the Indus Civilisation—provides us with an opportunity to examine the dynamics of cropping systems that incorporate both the growing of more than one crop in one season *and* the growing of crops in more than one season.

## Indus Subsistence and the Issue of ‘Multi-cropping’

The urban phase of the Indus Civilisation (c. 2600–1900 BC; Fig. 1) has long been characterized as a flourishing, culturally-integrated early complex society with a number of distinctive attributes, including: major urban settlements or cities surrounded by substantial fortification walls and/or built on platforms; houses, drains and wells made of mud- and/or fired-brick; and a distinctive material culture assemblage produced using a range of complex craft production techniques (see Lal 1997; Kenoyer 1998; Possehl 2002; Chakrabarti 2007; Wright 2010). Since the 1980s, however, there has been increasing recognition that there was a degree of cultural and geographical variation across the zone occupied by Indus populations (e.g. Possehl 1982, 1992, 2002; Vishnu-Mittre and Savithri 1982a, b; Joshi 1984; Meadow and Kenoyer 1997, p. 139; Weber et al. 2010; Wright 2010, pp. 180ff; Ajithprasad 2011; Petrie 2013; Petrie et al. 2017), and numerous authors have proposed that there was regional variation in subsistence practices (Vishnu-Mittre and Savithri 1982b; Chakrabarti 1988; Weber 1999, 2003; Fuller and Madella 2002; Singh et al. 2008; Weber et al. 2010; Petrie 2013; Petrie et al. 2016, 2017).

From the beginnings of Indus research, the issue of seasonality and single or multiple season cropping has been highlighted. Early excavations at the urban settlement of Mohenjo-Daro, for example, revealed evidence of the exploitation of wheat, barley (Mackay 1931, pp. 586–587; Luthra 1936), and field pea (Wheeler 1968, pp. 84–85), all of which would have been grown with the support of late summer inundation resulting from Himalayan snow-melt and monsoon rain in the regions to the northeast, complemented by winter rain (see Miller 2006, 2015; Petrie 2017). Thinking primarily about the areas of Baluchistan and Sindh, Fairservis (1967, 1971) subsequently hypothesised that winter or *rabi* cultivation was the norm for the Indus region. It has, however, long been argued that summer or *kharif* cultivation was also important (Vishnu-Mittre and Savithri 1982a, b; Weber 1989, 1991). Excavations at the Indus site of Rojdi in Gujarat quantitatively demonstrated that there was more to Indus cereal exploitation than wheat and barley, with the discovery of a sequence of occupation dominated by summer crops, particularly *Eleusine* sp., *Panicum sumatrense*, *Setaria* cf. *pumila* and *Setaria* cf. *italica* (Weber 1989, 1991, 1999). These attestations helped clarify earlier discoveries of what proved to be *Setaria* and *Eleusine* at Surkotada, also in Gujarat, where they were found with a mixture of mainly wild plant species (Vishnu-Mittre and Savithri 1982a, b, p. 214; Vishnu-Mittre 1990, pp. 388–391). The archaeobotanical evidence from these Indus sites in Gujarat was subsequently used to support a model of winter/*rabi* cropping in the ‘core’ and summer/*kharif* cropping in the ‘periphery’, where the periphery was regarded as unusual and not representative of the situation across the Indus Civilisation as a whole (Fuller and Madella 2002, pp. 353–355). Fuller and Madella (2002, p. 355) also suggested that ‘core’ areas practised more intensive agriculture, whereas populations in the summer cropping areas utilised more extensive systems.

It is now clear that there is a range of data suggesting that the model of winter/*rabi* core and summer/*kharif* periphery is too simplistic. Since the 1980s, it has been argued that Indus populations engaged in multi-cropping (Vishnu-Mittre and Savithri 1982a, b; Chakrabarti 1988, p. 96; 1995, p. 50; Weber 2003, p. 181), particularly in the areas of northwest India where it might have helped to buffer risk (Fuller and Madella 2002, pp. 354–355). However, the archaeobotanical evidence that might support this contention is not definitive, and there has been a lack of quantified archaeobotanical data sets from different locations within individual regions (Petrie et al. 2016, 2017). Excavations at Harappa have revealed evidence of the exploitation of crops grown in two seasons (see below), with an apparent increase over time in summer crops alongside continued use of winter crops, and this led Weber (2003, p. 181) to argue that ‘a complex multi-cropping strategy is evident in all periods of occupation’, although this strategy has not yet been characterised in detail. Winter and summer crops have also been discovered at a number of Indus settlements in northwest India, including Balu, Kunal, Banawali, Farmana, Rohira, Hulas and Sanghol (e.g. Saraswat 1986, 1993; Saraswat et al. 2000; Saraswat and Pokharia 2002, 2003; Weber et al. 2011; Kashyap and Weber 2013; Weber and Kashyap 2016). However, the precise date at which particular crops were being used at all of these sites has been unclear (Petrie et al. 2016). Fuller and Madella (2002, p. 354) have also suggested that the eastern zone of the Indus has evidence of ‘double-cropping’—using the term to denote the growing of both winter and summer crops. They argue that this was an extensive approach to agriculture, rather than one of the more intensive approaches used in some of the core regions of the Indus zone (Fuller and Madella 2002, p. 354).

More recent research has speculated that practices across the entire Indus zone and within individual areas were almost certainly far more varied than previously appeared, but there have still been limitations to the available data. For example, Singh and Petrie (2009) proposed that variation in local environmental conditions and water supply necessitated variation in farming practices within northwest India. Furthermore, Weber et al. (2010) hypothesised differences in agricultural strategy between Harappa, Mohenjo-Daro and Lothal based on variations in environment and rainfall. However, neither of these conjectures was supported by systematically collected archaeobotanical data. As Petrie (2013, p. 93) has noted, however, the true level of variation in practices will only be clear when evidence for the proportional exploitation of individual plant species in different regions and from different settlements within regions is widely available. Although there has been a shift in thinking away from simple seasonal dichotomy models, there has been little critical evaluation of the nuances of Indus cropping, and a looseness in the use of the terms *double*- or *multi*-*cropping*. Furthermore, there has been limited consideration of the implications that these terms have for discussion of adaptation to the wide environmental variability in the Indus region (Petrie et al. 2016, 2017). It is thus time to formulate a coherent theoretical foundation with which to characterise extant and future Indus archaeobotanical assemblages. Such an approach will allow us to coherently describe the diversity of Indus cropping strategies that can be detected and the degree to which different types of multi-cropping can be differentiated based on the available data sets.

## Differentiating Indus Cropping Strategies

Archaeobotanical remains have been recovered from only 55 of the 140+ Indus settlements that have been excavated, and systematic flotation and full publication of assemblages remains extremely rare (Fuller and Madella 2002, Table 1; Bates 2016, appendix 1). The available archaeobotanical evidence demonstrates that across the entire Indus zone, farmers grew a wide range of crops, including cereals, pulses, oilseeds, fibres and fruits (Table 1; see Weber 1999; Fuller and Madella 2002; Wright 2010; Bates 2016), with crops of each major type being grown in summer and winter. It is important to note, however, that variation in the distribution of rainfall and thus the supply of water in general means that it was not feasible to grow all of these crops in all of the regions occupied by Indus populations (Wright 2010, p. 168; Petrie 2017; Petrie et al. 2017). Miller (2006, 2015) has suggested that in central Punjab, water supply for Indus farmers is likely to have come primarily from inundation (produced by a combination of snowmelt and run-off from the Indian Summer Monsoon) and direct rainfall, while additional water was probably obtained via small-scale irrigation or well/lift irrigation. It is likely that these water supply mechanisms were used across the Indus zone, at differing levels of intensity, depending upon local environmental and climatic conditions (Petrie 2017). It is not yet possible to reconstruct the distribution of winter and summer rainfall across the Indus zone throughout the third millennium BC. However, modern instrumental rainfall data suggests that some of the areas inhabited by Indus populations are likely to have received direct winter rain, some probably received direct summer rain, some will have received direct rain from both systems, and others are not likely to have received direct rain from either (Fig. 2; Petrie 2017; Petrie et al. 2017; see Miller 2006, 2015). Furthermore, the steepness of the rainfall gradients means that there were likely differences in the quantity of direct rainfall that was received, with most areas receiving relatively limited direct rainfall (Fig. 2; Petrie 2017; Petrie et al. 2017). Petrie et al. (2016, 2017) have suggested that variation in local environmental conditions, vegetation, rainfall, and water supply would have necessitated distinctive adaptations for successful farming in different regions, including strategies relying on either winter crops like wheat or barley or summer crops like millet in some areas, or combinations of summer and winter crops.

**Table 1:** Major winter (*rabi*) and summer (*kharif*) crops grown by Indus Civilisation populations. Adapted from Weber 1999, table 1; 2003, table 5.2; Fuller and Madella 2002, table 2; Wright 2010, box 6.3; Bates 2016, tables 4.3, 4.5)

Winter (*rabi*) crops		Summer (*kharif*) crops	
*Cereals*			
Barley (*Hordeum vulgare*)	A	Rice (*Oryza* cf. *sativa*)	A
Wheat (*Triticum* sp.)	A	Signal grass millet (*Brachiaria ramosa*)	A
		Sawa millet (*Echinochloa colona*)	A
		African finger millet (*Eleusine coracana*)	A
		Proso millet (*Panicum miliaceum*)	A
		Little millet (*Panicum sumatrense*)	A
		Kodo millet (*Paspalum scrobiculatum*)	A
		Pearl millet (*Pennisetum glaucum*)	A
		Foxtail millet (*Setaria italica*)	A
		Yellow foxtail millet (*Setaria pumila*)	A
		Sorghum millet (*Sorghum bicolor*)	A
*Pulses*			
Lentil (*Lens* cf. *culinaris*)	A	Black bean (*Vigna mungo*)	A
Pea (*Pisum*)	A/P	Mung bean (*Vigna radiata*)	A/P
Chick pea (*Cicer*)	A/P	Moth bean (*Vigna acconitifolia*)	A
Sweet pea (*Vicia/Lathyrus*)	A	African gram bean (*Vigna* cf. *trilobata*)	A/P
		Horse gram bean (*Macrotyloma* cf. *uniflorum*)	A
*Oilseeds*			
Linseed/flax (*Linum usitatissimum*)	A	Mustard (*Brassica*)	A/P
		Sesame (*Sesamum indicum*)	A
*Fibres*			
Linseed/flax (*Linum usitatissimum*)	A	Cotton (*Gossypium arboreum*)	A/P
		Hemp (*Cannabis*)	A/P
		Jute (*Corhorus*)	A/P
*Fruits*			
		Cucumber/melon (*Cucumis*)	A
*Longer lived perennial fruits*			
Jujube (*Zizyphus*)	P	Date (*Phoenix*)	P
		Grape (*Vitis*)	P

‘A’ indicates annual and ‘P’ indicates perennial plant. ‘A/P’ indicates a plant that can be either. We have separated out longer-lived perennials here as they are not strictly winter or summer crops, though they do flower at specific times

**Fig. 2a:**
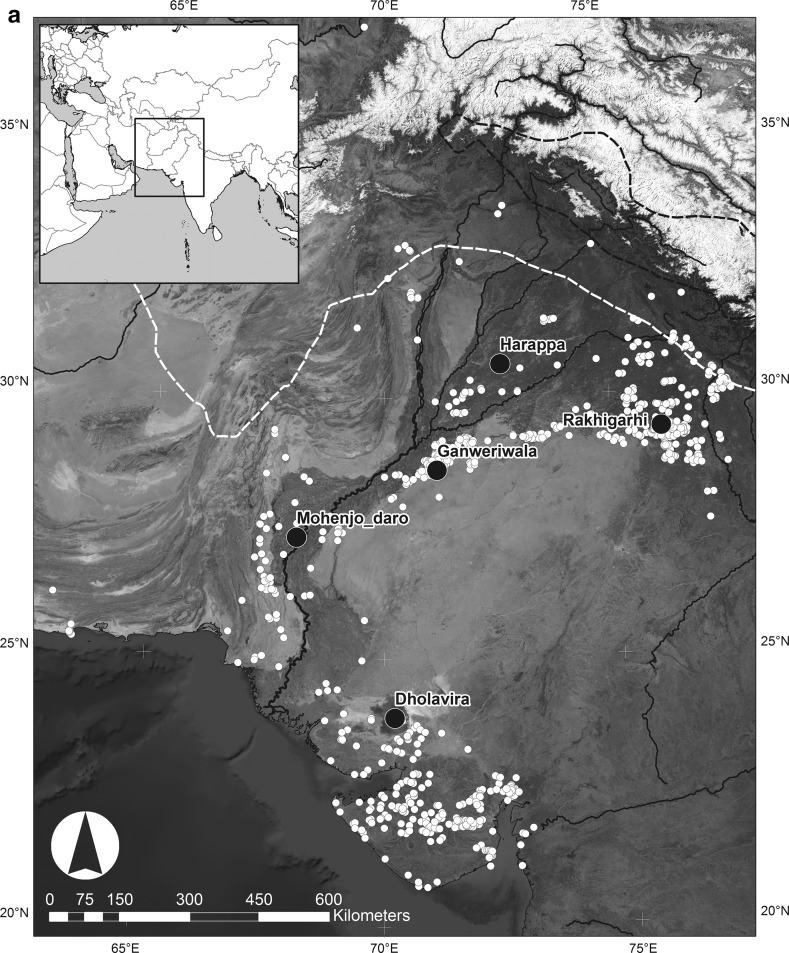
Distribution of modern mean winter (Jan–Mar) rainfall in relation to urban phase settlements. Urban centres are shown as *black circles* [*red* in online version], and other known urban period settlements are shown as *white* circles [*orange* in online version]. The *white dashed line* is the 100 mm isohyet, and the *black dashed line* is the 300 mm isohyet. Data extracted from Univ. of Delaware monthly global gridded high-res station (land) data set of precipitation from 1900 to 2008 (v2.01) by D.I. Redhouse

**Fig. 2b:**
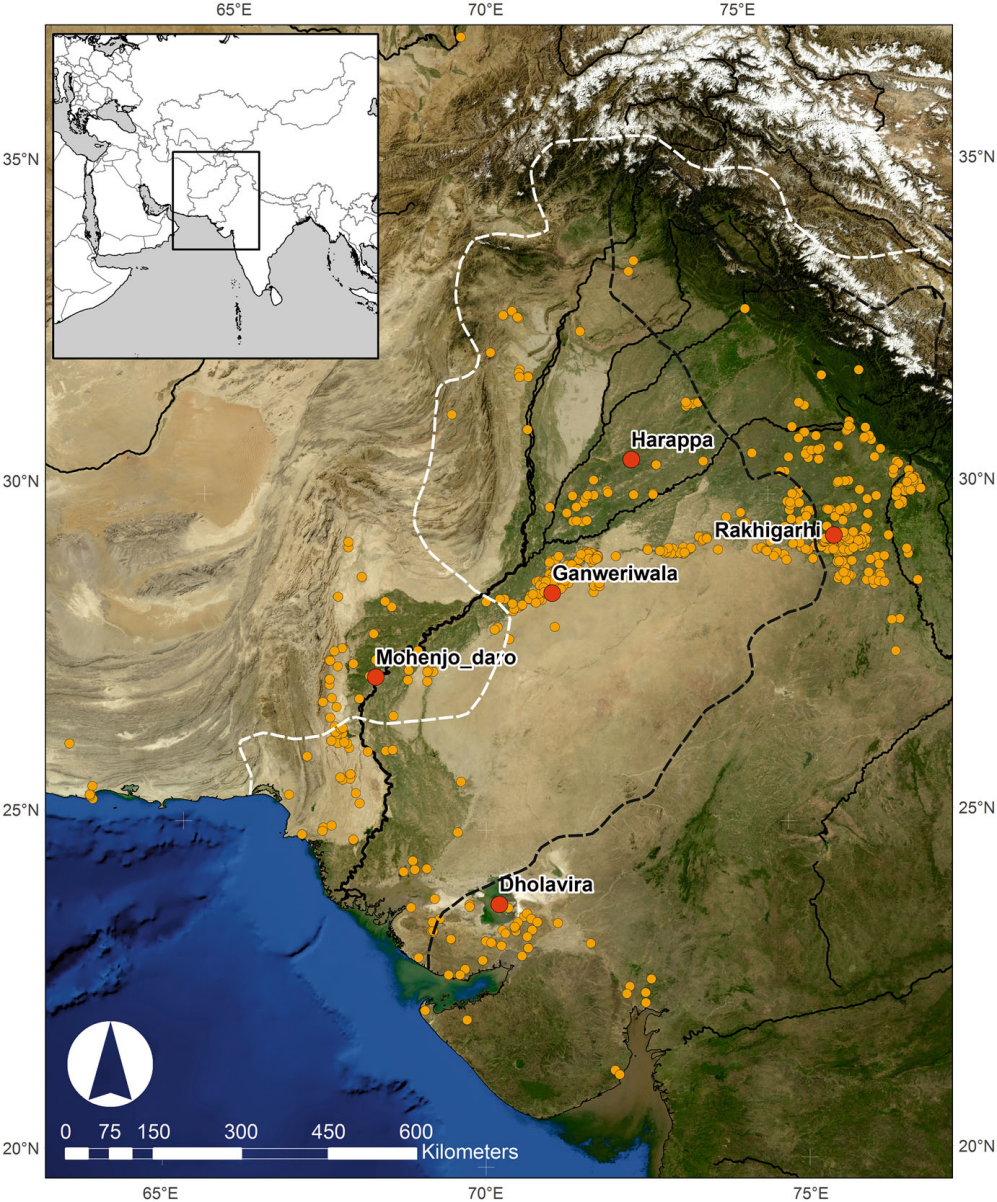
Distribution of modern mean summer (Jun–Aug) rainfall in relation to urban phase settlements. Urban centres are shown as *black circles* [*red* in online version], and other known urban period settlements are shown as *white circles* [*orange* in online version]. The *white dashed line* is the 100 mm isohyet, and the *black dashed line* is the 300 mm isohyet. Data extracted from Univ. of Delaware monthly global gridded high-res station (land) data set of precipitation from 1900 to 2008 (v2.01) by D.I. Redhouse

**Fig. 3:**
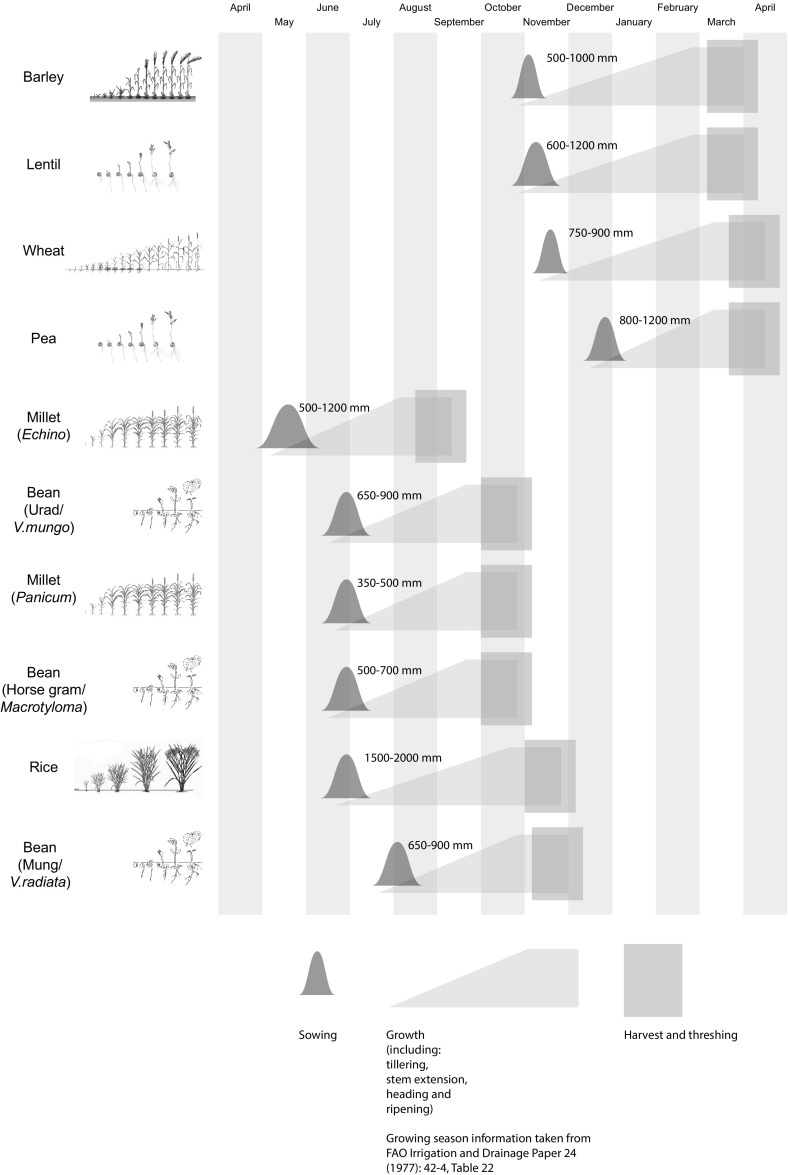
Comparison of the sowing times, growing periods, water requirements and harvest times of major Indus crops. Data primarily obtained from FAO Irrigation and Drainage Paper 24 (1977): 42–3, table 22.B; ECOCROP (2016)

The variation in water supply across the Indus zone (Petrie 2017) has implications for the dynamics of cropping that are explored here, as does the nature and timing of the sowing/growing/harvesting cycle for each crop. It is not yet possible to accurately reconstruct the growing cycles of ancient Indus crops, but assessment of data on modern varieties of the most common crops shows that each has distinctive sowing times, growing periods, water requirements and harvest times (Fig. 3, Table S1; see Food and Agriculture Organisation of the United Nations, www.fao.org).

The archaeological identification of cropping strategies presents several challenges, not least the need for good stratigraphic and taphonomic control. The only piece of direct archaeological evidence for Indus cropping is the remains of a field excavated at Kalibangan in northern Rajasthan (Lal 2003, pp. 95–98). The excavators noted morphological similarities between the criss-cross furrow marks in this Early Harappan field and the modern practice of growing chickpea at the edge of mustard crops (Lal 2003, pp. 95–98), though this association was not supported by microscopic archaeobotanical analysis of the field deposits themselves, which might have revealed the presence of specific types of phytoliths.

It is essential to recognise that taphonomic and preservation issues directly affect the archaeobotanical material that ends up in an archaeological context and whether it survives in the excavated record (Bates 2016, pp. 165–195). Most Indus contexts from which archaeobotanical samples have been collected contain multiple taxa. For example, Weber (2003, p. 179) has noted that at least three, and occasionally more than twenty, different species were present in any one deposit at Harappa, and pointed out that a range of taphonomic processes might have led to this mixing of crops in each instance. Many archaeological contexts represent mixed and multiple events, and can include crop remains that have been combined during growth, harvest, processing, cooking, waste removal, or even from post-human-use situations, such as bioturbation and post-depositional mixing.

Furthermore, the macrobotanical component of an archaeological assemblage is primarily made up of charred remains, and an additional complication comes from the fact that there are different pathways to preservation. Fuller et al. (2014) have pointed out that the charred remains are not those that are going to be eaten or used, but are related mainly to the final stages of crop processing before preparation for consumption, and as such do not necessarily directly represent what was grown in the fields. Furthermore, carbonised material may be the product of the burning of animal dung, and while such material will provide evidence of cultivation practices, this need not directly relate to human diet. During the charring process, the lighter and more fragile elements such as chaff or small weeds are more likely to be destroyed than the larger, denser elements such as cereal grains, and low temperatures (c. 350 °C) are better for good preservation, whereas higher temperatures will incinerate remains or render them unidentifiable (Boardman and Jones 1990). Whether crops are preserved is also partly dependent on the specific requirements for their use, for instance, hulled millets and pulses like *Vigna* sp. and *Macrotyloma uniflorum* require parching to make the hulls more fragile before removal (Reddy 1997, 2003; Fuller and Harvey 2006). In contrast, many cereals (e.g. wheat, barley and rice), fruits and some oilseeds rarely meet fire before final use, and some, such as fibre crops, are unlikely to encounter fire at all. Interpretation must thus invariably be subject to caveats.

Much of what can be said about Indus cropping is based on inference. For instance, we know that some crop species cannot be grown together on the same land (Table S1). Although documented as maslin crops in various regions (e.g. Halstead and Jones 1989; Jones and Halstead 1995), barley (*Hordeum vulgare*) and wheat (*Triticum* sp.) may not have been intercropped in close proximity (like the row or mixed intercropping found in less marginal situations) because of their different water and fertilisation requirements, and the competitive nature of barley. Thus when both are present, there is some likelihood that they were being grown as separate mono-crops (ECOCROP 2016). In some cases, the need to keep species apart is more acute. For example, rice (*Oryza* sp.) must be grown separately from sawa millet (*Echinochloa colona*), as the millet is highly competitive and extremely aggressive towards rice (Galinato et al. 1999). *Vigna mungo* is not a successful intercrop with rice as it reduces yields (Sengupta et al. 1985), though it can be row intercropped with *Sesamum* sp., which can also be intercropped with millet, as they all have similar water, fertility, soil, salinity and pH requirements. *Sesamum* sp. and rice are not suitable for being intercropped, however, because rice has greater water requirements than sesame and shallower soil planting needs. *Macrotyloma* cf. *uniflorum* is usually a mono-crop due to the labour-intensity of growing it as a row or ratoon crop of rice and sesame (ECOCROP 2016), but it can be grown in this way with more intense effort, as the growing requirements of each of these crops are similar. Furthermore, certain species, such as mung bean (*Vigna radiata*), moth bean (*Vigna acconitifolia*) and mukni bean *(Vigna trilobata*), can be perennial and grow over multiple years, if permanent patches of land are allocated. Other pulses, such as the pea (*Pisum* sp.) and various beans (*Vigna radiata, V. mungo, V. acconitifolia*, *V. trilobata*), are climber/prostrate species. Climber species in particular compete with cereal crops for height or space through climbing in a similar fashion to other vine species like bindweed. Although climbing peas grown with cereals have been observed ethnographically (Peña-Chocarro 1999, p. 167), efforts may well have been made to keep them apart. Alongside these pulses there are also some competitive vegetable/oilseed crops that out-compete other crops. There is less ambiguity with species like *Coccinia* cf. *grandis*, which is extremely aggressive and produces a thick, plant-killing blanket of material on a perennial basis, though it is often grown round the edges of fields (Xaygnalis et al. 1998). Perennial and climber/prostrate species may well have been grown on discrete plots of land (see Wright 2010, p. 168) or have required extremely careful management and intense labour input to ensure yields of other crops were not affected by their growth. Fruit exploitation can also lead to the allocation of separate parcels of land if the fruit is grown as orchard crops (see Wright 2010, p. 168). *Ziziphus mauritiana* is a common fruit at Indus sites, but it is not an orchard fruit like dates or citrus fruits, and instead is often grown at field edges or even in settlements. This does not suggest the setting aside of areas of land specifically for fruit crops, but rather more opportunistic exploitation.

In contrast to these conflicting requirements, *Echinochloa* sp., *Setaria* sp. and *Panicum* sp. share similar ecological and crop processing requirements and can therefore potentially be grown as mixed intercropping maslin crops (de Wet et al. 1983b). These are hardy and plastic millet species which can survive in a range of conditions. Furthermore, the presence of various annual pulse species, such as chickpea and lentil species, may indicate some type of row or strip intercropping alongside crops such as wheat and barley which share similar growth requirements, as the pulses would not require separate parcels of land all year round, and are advantageous to the growing of cereals because they enrich the soil with nitrogen (Agegnehu et al. 2006). Several other combinations of crops can be grown as ratoon crops to increase soil fertility (Table S1).

There is also a range of issues relating to the degree to which farmers employed techniques to increase yield as part of intensification or extensification strategies. *Intensification* refers to a set of strategies for ‘obtaining higher productivity over a period of time from the same land than could be obtained by simpler means’ (Brookfield 1986, p. 178; Morrison 1994) and includes specialisation, diversification and intensification proper (Kaiser and Voytek 1983), while *extensification* is increasing yield by using more land over a given time (Halstead 1992, Stevens 1996). Fuller and Madella (2002, pp. 353–355) have suggested that different strategies to increase yields were exploited across the Indus Civilisation, and argued that areas of the eastern Harappan region saw use of a strategy that was more extensive (i.e. diverse) than the more intensive specialised single-season core strategies. Evidence for the exploitation of different areas for farming has been demonstrated during Rojdi period C (c. 2000 BC; Weber, 1991, pp. 135–138), which saw the introduction of wetland sedges.

**Fig. 4:**
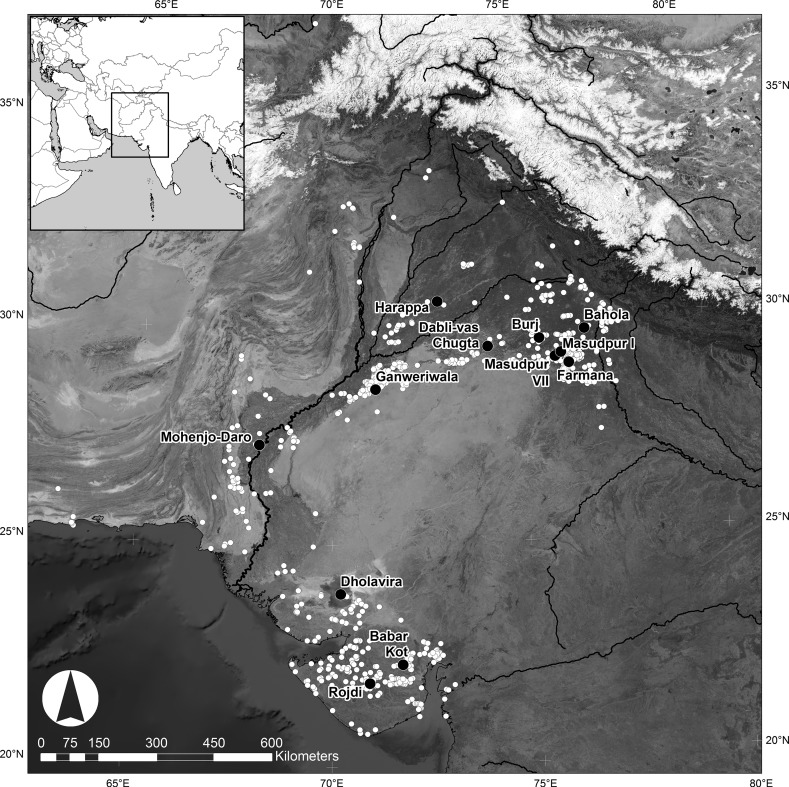
Location of Indus settlements discussed in this paper shown as *black circles* [*red* in online version]. Other known Indus settlements shown as *white circles* [*orange* in online version]

Only a subset of the archaeobotanical assemblages from Indus settlements have been published in such a way that they can be used to explore differences in cropping strategies. These include Harappa in Pakistani Punjab (Weber 2003), Rojdi (Weber 1989, 1991, 1999) and Babar Kot in Gujarat (Reddy 2003). Reddy’s (2003) analysis of material from Oriyo Timbo is also well published, but she concluded that the crops present were not cultivated by the inhabitants, so this assemblage will not be included here. The evidence for cropping at Harappa, Rojdi and Babar Kot will be contrasted with new data from excavations by the *Land, Water and Settlement* project in northwest India (Fig. 4).

### Harappa

Systematic archaeobotanical sampling at the urban city-site of Harappa (Fig. 4) has shown that a wide variety of crops were used there over time (Weber 1999, Table 3; 2003, Table 5.2). The agricultural strategies appear to have been dominated by the winter crops wheat and barley, though grains of summer crops, particularly ‘little millet’ (assumed to be *Panicum sumatrense*), were also recovered from the pre-urban Early Harappan period onwards (Weber 2003; Weber et al. 2010; Weber and Kashyap 2016). Using these data, Weber (1999, Table 1) has proposed a hierarchy of crop importance, with cereals forming a first tier; pulses, oilseeds, fibres and fruits forming a second; and a third being comprised of melons and legumes for forage. The diversity of this crop assemblage is the basis of Weber’s (2003, p. 181) assertion that agriculture at Harappa was characterised by a ‘complex multi-cropping strategy’.

The archaeobotanical assemblage from Harappa is still under analysis, but the data that have been published suggest that although multiple crops were being exploited, the growing of crops other than winter cereals is relatively minimal (Petrie et al. 2016). Summer crops, principally *Panicum* sp. millet, but also pulses, cotton and fruits, have a ubiquity of 9% in the Early Harappan period; this increases to 19% in the Mature Harappan and 47% in the Late Harappan period (Weber 2003, Table 5.3.a), with ubiquity being a measure of the percentage of samples from which specific taxa were recovered. These data suggest that over time, summer crops appeared in more contexts. However, these ubiquity statistics can be misleading, as Weber (2003, Table 5.3.c) has noted that in the Early Harappan period summer crops only equate to 2% of the overall charred crop assemblage in terms of relative abundance, and this only increased to 4% in the Mature Harappan and 7% in the Late Harappan period. In parallel with this minor increase in the relative abundance of summer crops over time, there was a marked decline in the relative abundance of winter crops, from 96% to 83% and then 77%, but the main source of increase is in the ‘weeds/unknown/other’ category (Weber 2003, Table 5.3.c).

Interpretation of these data is further complicated by plant morphology, particularly the fact that while *Panicum* sp. millet produces many more seeds per head than either wheat or barley, millet seeds are smaller and less calorific per grain (Bates et al. 2017b). Therefore, while summer crops were present at Harappa throughout the sequence, it is arguable that the preserved quantities of seeds indicate that they were a relatively minor component of the overall crop assemblage, particularly in contrast to the exploitation of wheat and barley. It is in fact plausible that the presence of such low quantities of summer crops indicates the actual transport of grain to Harappa from farther afield, rather than local exploitation, though this suggestion would be invalidated if evidence for the processing of summer crops at the site were preserved (Petrie et al. 2016).

The relative abundance of winter and summer crops at Harappa thus suggests the possibility that, if it was practised at all, sequential multi-cropping only ever formed a relatively minor component of the overall cropping strategy, even in the Late Harappan period. The winter crops wheat (*Triticum* sp.) and barley (*Hordeum* sp.) are by far the most commonly found taxa in all periods, though there was certainly a shift in which of these two crops was dominant over time, from barley to wheat and then back to barley (Weber 2003, Table 5.3.c). The presence of these summer crops, and the progressive increase in the use of summer and drought-tolerant crops, supports Weber’s (2003, pp. 181–189) assertion that the diversity of the crops exploited at Harappa increased over time, potentially related to the desire to create a more dependable food supply throughout the year. The details of the associated weed suite and the contextual details of the archaeobotanical assemblages are not yet available, so at present it is not possible to conduct a more complex analysis to assess whether the wheat and barley were part of a ‘within season’ intercropping system, or were being grown as parallel mono-crops in separate fields (i.e. a two-crop strategy; see Jones and Halstead 1995). The presence of brassicas (‘mustard’) and lentils does, however, suggest that some type of intercropping involving pulses was possible, though the preserved quantities of each suggest that this was likely small in scale.

### Rojdi

Weber’s (1989, 1991, 1999) analysis of material from the small Indus settlement site of Rojdi (Fig. 4) also demonstrated the use of a wide range of crops, but highlighted a very different cropping pattern to that seen at Harappa, with an assemblage dominated by millets and other summer crops. However, at no point does the assemblage consist entirely of crops grown in one season, and the proportions of the component crops remained fairly constant over the sequence. In Phase A (c. 2500–2200 BC) summer crops formed c. 98% of the crop assemblage, with *Eleusine* sp. millet being the dominant crop (although this identification of *Eleusine* has since been questioned: Fuller 2006, 2011), alongside some *Panicum miliare*, with a very small winter crop component of barley; in Phase B (c. 2200–2000 BC), summer crops increased slightly to 99% of the assemblage, with *Panicum miliare* being dominant, and a very minor winter component (1% of the crops) composed of *Brassica* sp. (mustard); and in Phase C (c. 2000–1700 BC) different millets in the form of *Setaria glauca* and *Setaria* cf. *italica* became a component of the summer crop assemblage. As with *Eleusine*, identifications of *Setaria italica* have since been questioned (Fuller 2006, 2011; Stevens et al. 2016). Summer crops proportionally dropped to 91% and winter crops increased to 9% of the overall crop assemblage, and winter crops increased in terms of the range of species present, as lentil, *Lathyrus* sp., *Vicia* sp. and flax (*Linum* sp.) were present along with the mustard (data acquired from converting densities into percentages of crop assemblage from Weber 1989, p. 270, table 18; p. 299, table 23; p. 315, table 29; pp. 366–367, table 33). Although crops from two seasons were present, the cropping strategies in Rojdi Phases A, B and C were almost entirely focused on the summer growing season. A slight decline in the role of summer crops was seen in the final mixed Late Harappan/Early Historic material of Phase C/D, with a reduction to 87% of the overall assemblage, and a mix of all three millet genera being attested, along with barley, *Lathyrus* sp., *Vicia* sp. and flax (data converted from density table in Weber 1989, pp. 366–367, Table 33). Weed species and a brief discussion of their ecologies are published from this site, but there is only limited consideration of the significance of those ecologies for understanding cropping strategies beyond their inclusion in the seed morphology descriptions (Weber 1989, pp. 194ff).

It is arguable that while the focus on the summer season cropping continued throughout the sequence at Rojdi, in terms of relative abundance and proportions, slightly more sequential multi-cropping might have been practised at Rodji than at Harappa. Looking at the species, in Phase A, *Eleusine* sp. millet was the most commonly found crop and formed the majority of the samples in which it is found, which could indicate mono-cropping. However, Weber (1989) also observed that *Eleusine* sp. was commonly found with some *Panicum miliare*, which could suggest intercropping, though as he notes ‘it is unclear whether the occurrence of these seeds together represents mixing at times of cultivation, processing or use’ (Weber 1989, p. 275). In Phase B, however, there was a change to *Panicum miliare* as the dominant crop, which could again indicate mono-cropping, though it is unclear whether the millet occurred only with weeds or with other crops. In Phase C there was a further change, with *Setaria* sp. becoming dominant. Weber (1989) noted the mixing of *Setaria* cf. *glauca* and *Setaria* cf. *italica*, and pointed out that each species has different management needs, with *S. glauca* preferring little management while *S. italica* needs heavy weeding. The difference in these requirements suggests that strip intercropping or mono-cropping of millets in separate fields was practised in Phase C. In Phase C/D, a different pattern was again seen, in which three millets were commonly found. These millets have similar husbandry and processing requirements and therefore could have been grown together, but there is little information on how commonly they are found mixed in individual samples, which makes it difficult to determine whether they were mono- or inter-cropped.

### Babar Kot

Further evidence of the summer-crop-dominant cropping regime has been documented at other Gujarati Harappan settlement sites such as Babar Kot (Fig. 4; Reddy 1994, 2003), in the semi-arid region of Saurashtra. Three ‘occupations’ attributed to the Harappan period were found dating to the late Mature Harappan (I) and transitioning into the Late Harappan periods (II, III) (Reddy 1994, 2003). The archaeobotanical crop assemblage shows an even more intensive focus on a summer cropping regime than at Rojdi, with summer crops forming: 94.3% of the crop assemblage in Occupation I, with a minor component of lentil forming the other 5.7%; 99.7% in Occupation II, the other 0.3% formed by lentil, *Lathyrus* sp. and flax; and 99.8% in Occupation III, with the other 0.2% composed of a minor inclusion of flax, *Brassica* sp. (cf. mustard), lentil, *Vicia* sp. and *Ziziphus* sp. (data obtained by deriving percentages from the densities in Reddy 2003, p. 122). Weed species are published from this site (Reddy 1997, 2003), but statistical analysis of the relationship between the weed ecology and cropping practices has not yet been attempted.

Millets, including *Panicum miliare* and *Setaria italica*, were the main crops throughout the sequence, with the dominant species being dependent on period of occupation and context (Reddy 2003, pp. 122, 129–130). In Occupation I, *Panicum miliare* (Reddy 2003, p. 122) was the only millet present, so it is likely that it was grown as a mono-crop. In Occupation II there was a notable change with *Setaria italica* forming 79.88% of the millet assemblage while *Panicum miliare* formed 19.11% (Reddy 2003, p. 122), although identifications of *Setaria italica* have since been questioned (Fuller 2006, 2011; Stevens et al. 2016). Reddy (2003, pp. 129–130) noted that *Setaria italica* was found in separate pits from *Panicum miliare*, and also pointed out that there are different weed suites associated with these two crops. The lack of evidence for mixing of these two species in any one context makes it likely that they were grown as mono-crops in different fields. In Occupation III *Setaria italica* formed 92.5% of the millet assemblage, and ‘seed pockets’, or distinct clumps of seeds were also found (Reddy 2003, pp. 128–129), again suggestive of mono-cropping focused on one cereal species.

### *Land, Water and Settlement* Project Sites

The *Land, Water and Settlement* project has excavated a number of Indus village settlements across northwest India (Singh et al. 2009, 2010, 2012a, b, 2013a, b; Petrie et al. 2009, 2017). Flotation samples have been analysed from Early, Mature and Late Harappan period deposits at five sites: Dabli vas Chugta, Burj, Masudpur VII, Masudpur I, and Bahola (Table 2; Bates 2016; Bates et al. 2017a, b, in press; Petrie et al. 2016, 2017). Although they are all located in northwest India, each of the settlements excavated by the *Land, Water and Settlement* project was situated in a distinctive environmental and ecological zone (Table 3), which potentially enabled and constrained the cropping practices of local farmers.

**Table 2:** Phases of occupation excavated at different *Land, Water and Settlement* project sites discussed here

Code	Name	Trench	Early Harappan	Mature Harappan	Late Harappan	Painted Grey Ware	Black Slip	Early Historic
DVC	Dabli vas Chugta	ZA6		X				X
DVC		ZI7		X				
BRJ	Burj	ZA2	X			X		X
BRJ		ZG9	X			X	X	
MSD VII	Bhimwada Jodha	YA2	X	X	X			
MSD VII		YB1	X	X	X			
MSD I	Sampolia Khera	XA1		X	X			
MSD I		YA3		X				
MSD I		XM2		X				
BHA	Bahola	AB1			X	X		X

**Table 3:** Modern environmental context of the *Land, Water and Settlement* project sites. Data compiled from Fagan and Townsend 1915; Punjab District Gazetteers 1918; Spate et al. 1967; Pascoe 1973; Bhatia and Kumar 1987; Weber 2003; Yadav et al. 2005; Kottek et al. 2006; Department of Agriculture 2007a, b, c; Wright 2010; Singh et al. 2010, 2012b; Neogi 2013; iiss 2014; Weatherbase 2015

	Dabli vas-Chugta	Burj	Masudpur I and VII	Bahola
Köppen–Geigger climatic classification	Transition: hot arid steppe to hot arid desert	Hot arid steppe	Transition: hot arid steppe to hot, dry winter, hot summer	Hot, dry winter, hot summer
Landscape	Flat alluvial plains with sand dunes	Flat alluvial plains with occasional dunes	Flat alluvial plains with occasional dunes	Flat alluvial plains with occasional dunes
Proximate landforms	Thar Desert	N/A	N/A	Seasonal *nullah*
Site location	Margin of flood zone	Margin of flood zone; Slightly raised sandy landform	Far from river flood zone; (VII on fossil dune) (I on bedded sand)	Margin of flood zone; Natural mound
Av. Annual Temp (°C)	25.6	25.3	25.2	24
Av. Summer Temp (°C)	31.7	29.2	30.5	29.2
Av. Winter Temp. (°C)	19.6	19.4	18.5	18.7
Av. Hottest temp. (°C)	35	34.5	34	32.4
Av. Coldest Temp. (°C)	13.9	13.9	13.6	13.5
Av. Annual rainfall (mm)	304.4	361.5	490.7	675.9
% Summer rainfall	88%	88%	86%	88%
% Winter rainfall	12%	12%	14%	12%
Av. no. of days of rain annually	22.5	24.2	25.8	27.5
No. of rainy days (S)	18.4	18.4	20.4	20.8
No. of rainy days (W)	5.7	5.6	5.4	6.7
Av. groundwater depth (m)	1.6–25	3–10	3–10	Max. 7.6
River system	Ghaggar	Ghaggar	–	Yamuna
River seasonality	Summer flooding	Summer flooding	Limited summer flooding	Perennial with summer flooding
River flow (million acre feet)	0.5–2.5 MAF	0.5–2.5 MAF	0.5–2.5 MAF	3.19 MAF
% River flow Summer	100%	100%	100%	80%
% River flow winter	0%	0%	0%	20%
Distance from river (est.)	<0.5 km (Ghaggar palaeochannel)	<0.5 km (Ghaggar palaeochannel)	>50 km (Ghaggar palaeochannel)	<0.5 km *nullah* c. 25 km (Yamuna)
River temperament	Low energy flooding	Low energy flooding	Low energy flooding	High energy flooding
Soil texture	Sand with silt–clay below	Silt–loam, some sand	Sand–loam, some clay–loam	Sand–loam
Soil pH	8.3–8.4 Weakly alkaline	10.24 Alkaline	9.04 Weakly alkaline	6.5–8.6 Neutral—weakly alkaline
Soil nitrogen	Low	Medium	Low–medium	Low
Soil phosphates	Low	Medium	Low–medium	Low
Soil potassium	High	Medium	Low–medium	High
Soil salinity	Often high	Low	Low	Low

#### Using Weed Ecology to Understand Cropping Practices

Different crop and weed seeds have different specific ecological requirements (SI.1, Tables S1–S2i–xi), so the presence or absence of specific crops and weeds, and the combinations in which the two appear, provide insight into their growing conditions and hence the cropping practices that local farmers used in each context. A range of factors—including soil pH and fertility, plant fertility and water requirements—affect what will grow, where it will grow and whether it will grow with other plants; if the conditions are optimal, more plants will grow (Stevens 1932, 1957). Ellenberg (1950) and van der Veen (1992) have argued that the type of crop can influence the associated weed flora through factors such as leaf shade and rhythm of growth. Given that we know little about growing conditions in the past, the ecology of plants, especially weeds, is important for understanding what the environment was like and how it may have been altered by human action. Numerous archaeological studies have explored weed ecology (e.g. Jones 1981, 1984, 1985, 1986; van der Veen 1992; Stevens 1996), but they have all used different methods—mainly phytosociology, autecology and FIBS, each with associated benefits and problems (Bates 2016, p. 117).

Phytosociology is the classification of the associations of species into groups representative of the ecological conditions (Ellenberg 1974). It has been applied in archaeology especially in Central Europe, where a large number of modern phytosociological models have been developed (e.g. van der Veen 1992; Stevens 1996, 2003), but comprehensive modelling of phytosociological groups is lacking for agricultural systems in South Asia. Autecology looks at the relationship of each species to its environment, rather than the relationship between plants (Ellenberg 1988). Autecology studies are uncommon in South Asia (Rao and Nagamani 2010), and those that have been carried out have focused on individual species, for example *Eleusine indica* (Singh 1968; Singh and Misra 1969); *Cyperus rotundus* (Ambasht 1964); *Chenopodium album* (Misra 1969). The species studied tend to be those that have a major impact on modern agriculture, and systematic studies of the range of species present are still lacking. FIBS (Functional Identification of Botanical Surveys), or functional ecology, is an approach to plant ecology (see Garnier et al. 2016) that has been adopted by archaeobotanists in response to the problems of local ecologies and the patchy nature of studies (Charles et al. 1997). This approach proposed the use of ‘functional attributes’ from plants, such as canopy height, leaf height, root depth and type, seed bank formation and reproductive habits, to identify human actions such as irrigation, soil disturbance, and manuring (Charles et al. 1997; Bogaard et al. 1998, 1999; Jones et al. 2000, 2010). However, as Jones et al. (2000) have noted, there is not necessarily a direct link between some functional attributes and single activities, for example root depth could be an indicator of either water stress or soil disturbance. Also, although it has been used in Europe and the ancient Near East (e.g. Charles et al. 1997; Bogaard et al. 1998, 1999; Jones et al. 2000, 2010), the applicability of the FIBS approach to archaeological issues has not been systematically demonstrated elsewhere. Furthermore, for our purposes, it is notable that no FIBS surveys have yet been carried out in South Asia or applied to agricultural methods that are of interest to South Asian archaeology. Future field research that explores the functional adaptation of weeds in the South Asian context is clearly required in order to address these issues specifically.

Given the limitations of the extant data, a method combining autecology and a FIBS-type approach looking at physical aspects of the plant has been developed for exploring the weeds to elucidate questions of crop management in the Indus context (Bates 2016, pp. 119–120). This approach is adapted from the one used by Stevens (1996) in the Upper Thames Valley, where he combined autecology data with information on other aspects such as seed bank and root depth. As no single comprehensive autecology study for South Asia or the Indus Civilisation region is available, we have followed van der Veen (1992) and Stevens (1996) in using a combination of data sources, such as floras and published autecology studies, which helps with a number of issues. For example, the question ‘where were the plants (weeds and thus by proxy crops) growing?’ can be approached by considering soil structure, soil pH, and soil moisture; the question ‘how often were the fields being used?’ can be approached by considering the reproductive techniques of weeds, non-arable weeds, and nitrophobic species; the question ‘were manuring or drainage techniques being used?’ can be approached by considering nutrient indicator species; the question ‘was tillage being used?’ can be approached by considering reproductive techniques, seed dormancy, and root type; and the question ‘what weeding practices were used?’ can be approached by considering seed dormancy and germination time (Bates 2016, p. 120).

The archaeobotanical material presented below includes the macroscopic remains of grains of seed crops and weed species from five of the settlements excavated by the *Land, Water and Settlement* project (SI.2, Tables S3–S4). These sites are discussed from west to east, starting with the site likely to have received the lowest annual direct rainfall (Dabli vas Chugta). It is important to note at the outset that in general crops and weeds appeared in relatively low abundance at these settlements, apparently as a product of poor preservation in alternating wet/dry conditions and sites with shallow depth of deposit (Bates 2016; Petrie et al. 2016). The significance of specific combinations of crops and weeds that have been recovered from each of these sites will be discussed below, and this will be supported by consideration of the specific ecological requirements of various crop and weed species.

#### Dabli vas Chugta

The site of Dabli vas Chugta lies 7 km from Kalibangan in Hanumangarh District, Rajasthan (Fig. 4), and although it was initially believed to have been occupied during the Early Harappan period (Singh et al. 2012b), radiocarbon dates now demonstrate that it was occupied during the early phase of the Mature Harappan period. The crop assemblage is diverse, and includes cereals, pulses, fruits and oil seeds, though cereals dominate (Table S5). Winter crops form the majority of the crop assemblage at 58.95% and summer crops make up 24.91%, with the remaining 11.52% being unidentified/unidentifiable cereals or pulse fragments that cannot be attributed to a season and 4.61% belonging to fruits (Table S5; Bates 2016, p. 195). Barley (*Hordeum vulgare*), which prefers dry soils, was the most common crop, in terms of abundance, ubiquity and density (Table S5). In most contexts, barley was the only crop present, though in some of the earliest levels it was recovered alongside some millets, including *Setaria* sp. and *Panicum* sp. (Bates 2016, p. 134), which are both suited to poor soils (in terms of fertility and moisture), and are tolerant of both flooding and drought (Table S1). While present, other crops such as wheat (*Triticum* sp.), lentil (*Lens* cf. *culinaris*), mustard seed (*Brassica* sp.), and jujube berry (*Ziziphus mauritiana*) were not common (Table S5), and presumably less important in the cropping strategy, though significant quantities of indeterminate Fabaceae were present.

In the weed assemblage (Table S6), only *Avena* sp. could be definitely attributed to the winter growing season, though *Chenopodium* cf. *album* was also present and could also have been a weed of the winter season. *Avena* sp. is a common weed of barley and wheat, and given the dominance of *Hordeum vulgare*, its presence is not surprising. *Avena* sp. is most commonly seen in low water moisture conditions and acidic soils, and the presence of *Chenopodium* cf. *album* indicates high nitrogen conditions (see Table S2ii, iv, v), so the combination of the two suggests that there was a range of soil fertility in the fields around Dabli vas Chugta. Although both species are annuals neither responds well to tillage, so their presence might indicate that there was not a high level of soil disturbance. Several summer season weeds were attested (Table S6), with a range of ecological preferences (Tables S2, S7). Most prefer wet soils, though the proportion of flood- and non-flood-tolerant species is roughly equivalent, and some drought-tolerant species were also present (Table S7). This combination of weeds implies that water supply was inconsistent in summer, and included both flood and drought. Weeds that can tolerate any soil conditions or only specifically sandy soils were noted, and extensive changes to the soil matrix, fertility and pH are not evident (Table S7), which may suggest that heavy soil disturbance was not occurring. This pattern is supported by the life cycles of the weeds, as although the majority were biennial, the main reproductive method was a combination of seed bank and rhizomes, again suggesting a lack of significant soil disturbance (Table S7). The adaptability of the summer crops and the range of tolerances seen in the summer weeds suggest that summer cropping could have been practised on a broad range of land types around the settlement.

The seasons of the crops and the growing conditions indicated by the crops and weeds at Dabli vas Chugta suggest a strategy dominated by mono-cropping of barley, possibly with some sequential multi-cropping involving millet in the earliest phases. Wheat might have been grown as an additional mono-crop, and it may well have been grown in fields separate from the barley, as they have different water and fertilisation requirements, and because of the competitive nature of barley. It is possible that the millets were grown as mixed intercrops (see de Wet et al. 1983a). Exploitation of pulses and fruits appears to have been relatively limited, though their presence suggests possible intercropping of winter pulses with barley, wheat or both. Such pulses and fruits may well have been grown at the edges of larger fields, and the intercropping of *Hordeum vulgare* with *Brassica* sp., such as *Brassica juncea* and *nigra*, and also with *Lens culinaris*, is common today.

#### Burj

Burj is a one-hectare village settlement site in Haryana, India (Fig. 4), established in the Early Harappan period, but then apparently abandoned until the considerably later Painted Grey Ware period (Singh et al. 2010). The Early Harappan period archaeobotanical assemblage from Burj was poorly preserved, with a large proportion of crops of uncertain seasonality (Table S8), which makes it difficult to analyse in terms of cropping strategies. Notably, no summer crops were identified in the Early Harappan period deposits, and only *Hordeum vulgare*, the indeterminate category *Hordeum/Triticum*, and *Ziziphus* sp. were identifiable, suggesting that barley and jujube fruits were the dominant Early Harappan period winter crops (Table S8). The preservation conditions noted for the crops at Burj in the Early Harappan period also hold for the weeds. Only one weed type was noted—an indeterminate small grass—and so it is not possible to discuss weed ecology for Early Harappan Burj.

Archaeobotanical preservation in the PGW period contexts was notably better, and a very different pattern emerges. There is considerably more diversity in the crop species present, including cereals, pulses and fruits (Table S9). In contrast to the Early Harappan deposits, summer crops make up the largest proportion of the assemblage at 83.25% in the PGW period (including *Echinochloa colona*, *Setaria* cf. *pumila* and *Panicum* sp); winter crops form only 9.91%; tree/orchard fruits 5.9%; and uncertain-seasonality crops 0.94% (Table S9). These proportions suggest that, while there was a focus on summer crops, some sequential cropping was also being carried out in the PGW period, as seen at Rojdi Phase C/D. One cereal crop was again dominant, this time *Echinochloa* sp. millet, which was found predominantly on its own or with small quantities of other small grained millets (Table S9; Bates 2016, Table 7.5). It is notable, however, that small quantities of winter pulses were attested, including chickpea and a possible type of sweet pea (Table S9).

Within the PGW period weed assemblage, no winter weeds were identified, so it is not possible to make comparisons between seasons at the settlement. This apparent absence of winter weeds is interesting as some winter crops were present, suggesting either that weeds were removed during crop processing, or possibly that winter grain was imported from elsewhere. However, the presence of winter cereal chaff phytoliths and Pooideae leaf/stem elements (Bates 2016; Bates et al. 2017b) suggests that early stage crop processing, as well as late stage processing, occurred on or near the site. It is thus less likely that these cereals were being imported to the site as a whole cereal ear including stem and stalk than that they were being grown in the vicinity of the site. The lack of winter weeds could relate to differential pathways to preservation to summer weeds (for example, not reaching charring at the same rate) or processing taking place elsewhere at the site.

A number of summer weed species were present at Burj (Table S10), and display particular ecological preference indicators (Table S11). The weed preference for moist to dry soils and a low tolerance for flooding suggest that irrigation may not have been utilised, but the fact that most weeds had a low drought and flooding tolerance suggests that there may have been some control of the water regime. This pattern is interesting in relation to the proximity of Burj to the seasonal river and the geoarchaeology that has been carried out at the site (Neogi 2013), both of which suggest a high potential for low intensity but regular flooding. The pattern also implies that in the summer agriculture may have been carried out either away from the areas affected by overbank flooding or (more likely) was carefully managed to prevent both flood and drought conditions. The summer weeds can tolerate any soil conditions or only specifically sandy soils, with indications of a low nitrogen system and moderate fertility. There appears to be a lack of extensive changes to the soil matrix, fertility and pH, suggesting that heavy soil disturbance was not occurring. This pattern is again supported by the life cycles of the weeds, where although the majority were biennial, the main reproductive method was through both a seed bank and rhizomes. This weed ecology fits fairly well with the limited ecological data available for the three main summer crops, *Echinochloa colona*, *Setaria* cf. *pumila* and *Panicum* sp (see Table S1). The absence of high-intensity water management indicated by the weeds fits well with these millets, as does the implied moderate soil fertility and the range of soil textures. They can also grow in a range of conditions from dry lands to marshy situations, and are competitive (Galinato et al. 1999), fitting well with the models of past land use for the area (Table 3).

The co-occurrence of winter cereals and perennial but summer flowering fruits in the Early Harappan deposits could suggest some form of ‘mixed farming’ (Andrews and Kassam 1976, p. 3), with the fruit trees potentially growing at the edges of cereal fields. Unfortunately, the weeds provide little insight into this situation. During the PGW period, crop proportions suggest that mixed intercropping of millet was predominant in the summer season, but that the overarching strategy involved sequential multi-cropping when the winter season crops are taken into consideration. It is notable, however, that no macro remains of winter weeds were attested, despite the phytolith evidence suggesting their presence (Bates 2016, Bates et al. 2017b). Exploitation of pulses and fruits at PGW period Burj was even more limited than at Dabli vas Chugta. Intercropping of some of the pulses is possible. *Cicer* sp. and *Lathyrus* sp. are annual legumes, and in northwest South Asia they are often intercropped with barley and wheat, usually in row intercropping, which helps with nitrogen enrichment of soil (see Agegnehu et al. 2006). In contrast, *Pisum* sp. is a climber/prostrate annual, and would have been difficult to intercrop with cereals as it would compete for height, so discrete parcels of land for growing peas are a possibility. *Vigna radiata* is perennial when wild but annual when domesticated, so may not have needed separate land over multiple years. However, it is often shrubby in appearance, or climbs, suggesting that it might need a discrete parcel of land, though this could take the form of a carefully managed strip intercrop, which would improve the nitrogen content of soil. Jujube trees may also have been cultivated at the edges of fields as part of a mixed farming approach (see Andrews and Kassam 1976, p. 3).

#### Masudpur VII

The site of Masudpur VII is a one-hectare village-sized settlement site situated 18 km from the Indus city of Rakhigarhi in the modern state of Haryana, India, and was occupied in the Early, Mature and Late Harappan periods (Petrie et al. 2009, 2016; Fig. 4). The crop assemblage from Masudpur VII was mixed, including a range of cereals, pulses and fruits (Table S12). In the Early Harappan period at Masudpur VII, there is clear evidence of a more mixed cropping system than seen at other contemporaneous sites, with 24.68% winter crops and 58.87% summer crops, 7.59% tree/orchard fruit and the final 8.86% consisting of crops of unidentified seasonality (Table S12). Two crop species dominated, one winter and one summer: wheat and *Echinochloa* sp. millet. *Macrotyloma* cf. *uniflorum* and *Ziziphus mauritiana* were, however, present in significant quantities, and rice and other legumes (e.g. *Vigna radiata* and *Pisum* sp.) were also attested.

No winter weeds were attested in any of the phases of occupation at Masudpur VII, but a range of summer weeds were attested in the Early Harappan assemblages (Table S13). The ecological preferences of the Early Harappan summer weeds (Table S14) suggest moist, sometimes wet, soil conditions and some flood tolerance, which indicates that there may have been some control of water supply to avoid desiccation, though there is no evidence that this involved irrigation. This perhaps suggests active management of rainfall-induced flooding/inundation. No particular soil was preferred, and evidence for leaching is low as the weeds preferred alkaline soils and moderate fertility. There was a lack of Chenopodiaceae weeds, but some Fabaceae weeds were present, perhaps suggesting low nitrogen levels, which would fit with the soil model for the area (Table 3), and perhaps suggest there was no leaching caused by heavy soil disturbance. The majority of the weeds were biennial, which also supports the idea of low soil disturbance, as does the presence of both vegetative and seed bank reproduction. As such, it appears that limited management was needed to produce a good yield.

The proportions of wheat and *Echinochloa* sp. millet and the growing conditions indicated by the crops and the weeds at Early Harappan Masudpur VII potentially indicate that, although there was a summer focus, sequential multi-cropping was being practised. The presence of both rice and *Echinochloa* sp. suggests that these crops are likely to have been grown as spatially distinct mono-crops on separate land in summer. The climber/prostrate nature of pea (*Pisum* sp.) suggests that it is likely to have been grown as a mono-crop on separate land in different seasons (Tables 1, S1). This is also likely for *Macrotyloma* cf. *uniflorum*, due to the labour-intensity of growing it as a row or ratoon crop of rice and sesame (Table S1; ECOCROP 2016). *Coccinia grandis* would also have required either discrete spaces, or more likely, would have been grown at field edges, and the same is likely for fruit trees. Some *Cicer* sp., *Vicia/Lathyrus* and indeterminate oil/fibre seeds were present, and thus some intercropping is possible. Although it is difficult to be precise, the combination of crops present indicates considerable complexity in the crop management strategy throughout the year at Early Harappan Masudpur VII.

In the Mature Harappan period the assemblage as a whole remains mixed and includes cereals, pulses and fruits, all in significant quantities (Table S12). Two crops again dominated, this time barley and *Echinochloa* sp., and there was a shift towards winter crop dominance (52.38%), with summer crops forming 31.75% and tree/orchard fruits making up a slightly larger proportion than in the Early Harappan at 12.7% (unknown-seasonality crops continue to form a small part of the assemblage at 3.17%) (Table S12). However, rice grains were not attested, and *Echinochloa* sp. was the only millet species. Peas and lentils, which are winter pulses, were not present, but a range of summer pulses were attested, including *Vigna radiata*, *Vigna mungo* and *Macrotyloma* cf. *uniflorum*; *Ziziphus mauritiana* was also present.

As in the Early Harappan period, no winter weeds were found, but the presence of both early- and late-stage processing waste and phytoliths from winter crops implies that winter crops were likely to have been grown close to the site. The ecological preferences of the summer weeds in the Mature Harappan assemblage (Table S16) show some differences, with slightly wetter conditions showing more flood tolerance, perhaps indicating more water management. The higher proportion of drought tolerance, however, also suggests that the range of water conditions may have increased in this period. Weeds that can tolerate any soil conditions were again noted, but the proportions of sand-preference weeds were slightly higher than in the Early Harappan period. Combined with wetter conditions, the presence of sand-tolerant weeds could suggest greater leaching potential. Indeed, more neutral and acid soils were indicated in the Mature Harappan period, but the majority of weeds indicating specific soils suggested that alkaline or neutral soils were being used, and that soil fertility was moderate. Neither Fabaceae weeds nor Chenopodiaceae weeds were noted in this period in the weed assemblage, perhaps suggesting that the conditions remained moderate in nitrogen, which in turn suggests that there may have been little soil disturbance. The lack of purely annual species and species reproducing by seed bank might, however, indicate that ard tillage was being utilised, leading to light soil disturbance (see Stevens 1996).

The seasons of the crops and the growing conditions indicated by the crops and the weeds suggest a sequential multi-cropping strategy during the Mature Harappan phase at Masudpur VII, involving a duo-culture of summer and winter cereals. In the summer, *Echinochloa colona* may have been intercropped with some of the pulses (e.g. *Brassica* sp.), while other pulses are likely to have been grown as mono-crops (*Macrotyloma* cf. *uniflorum* and *Vigna radiata*), potentially on perennially allocated land for mung bean if it was a non-domesticated form. Fruit trees will have also required their own space, but the only one that could be identified was *Ziziphus mauritania*, as at all sites, and so it seems unlikely that orchards were present and field boundary trees were being exploited. Interestingly, fewer varieties of crops appear to have been used during the Mature Harappan period, perhaps indicating deliberate choice to minimise diversity and focus on particular crops (Table S12) (Bates in prep).

Only three samples were available to study for the Late Harappan period at Masudpur VII; it is therefore questionable how representative these are of the Late Harappan agricultural strategy at the settlement. The material from these samples suggests a return to diversity in the crop assemblage and to the dominance of summer cropping (80.17%), with winter crops forming 11.2%, tree/orchard crops 3.45%, and unknown-seasonality crops 5.17% (Table S12). *Echinochloa colona* continued to be grown, and rice reappears in the summer cropping regime, but the overall assemblage is dominated not by a cereal but by *Coccinia* cf. *grandis*, which is a type of gourd and possible famine food (Table S12). This finding could resonate with evidence for dietary stress and disease at Harappa and at the end of the Mature and into the Late Harappan phases (Robbins Schug et al. 2013a, b, Robbins Schug and Blevins 2016), and also with indications that there was a weakening of the Indian Summer Monsoon in northwest India c. 2200 BC (Dixit et al. 2014), which is a local northwest Indian manifestation of a much more widespread phenomenon (Madella and Fuller 2006; MacDonald 2011; Berkelhammer et al. 2012; Giosan et al. 2012). It could, however, be a taphonomic issue, as *Coccinia* cf. *grandis* was found in one of the three contexts where little else was recovered, perhaps suggesting that several gourds were burnt in a single event, which is likely to have skewed the crop proportions when compared to the other two multiple-event type contexts found (see Fuller et al. 2014 for discussion of the types of contexts that can be found on sites; following Hubbard and Clapham 1992). It could therefore be a case of low sample size being affected by an unusual sample type and/or event, rather than an indication of a major impact on diet and agricultural choices.

Again, there were no winter weeds, but a range of summer weeds was present in the Late Harappan deposits (Table S17), though it should be acknowledged that there was only a small number of samples from this period. The ecological preferences of the summer weeds in the Late Harappan deposits appear to show some return to the Early Harappan conditions and/or cropping choices, but also some differences (Table S18). Moist soil conditions were common, though drier conditions than in the previous two periods might be suggested, as the proportion of dry-tolerant or dry-preference weeds was greater and the proportion of flood-tolerant species decreased compared with the Mature Harappan period. However, there were still high proportions of summer weed species that were tolerant of neither drought nor flood. This pattern could suggest the range of land exploited had increased to include areas that were poorly watered but not drought-ridden. It also suggests less water management than in earlier periods. However, this observation should be tempered by the limited number of samples from the Late Harappan period mentioned above. A greater proportion of weeds with sandy soil preference were seen compared with previous periods, although the majority of weeds had no preference for soil texture. This could suggest more leaching potential, but alkali-preference weed proportions increased in this period, and fertility remained moderate. The nitrogen levels again appear to be low because of the presence of only Fabaceae, and the lack of Chenopodiaceae. These levels might suggest that soil disturbance was not particularly high (see Stevens 1996). While the majority of species found were biennial (i.e. can reproduce as annuals or perennials), there was a lack of purely perennial species, with 15% reproducing only through annual cycles, and no species relying on vegetation to continue on to a second year of existence. Both the biennial reproduction and reproductive method of the weeds identified in this period suggest some slight disturbance of the soil, and the lack of perennial weeds suggests some use of a plough. The Late Harappan weed ecology fits well with the evidence for millets because of their plasticity. The reduced flooding suggested by the weeds would also have been good for the growth of the millets, which do not like flooding during early growth, and actually fits well with the ecological preferences of dry-farmed rice (Fuller and Qin 2009).

It is difficult to characterise the unusual pattern seen in the Late Harappan period at Masudpur VII, particularly given the low sample size, but in principle, it is best described as a sequential multi-cropping strategy. Both winter and summer cereals and pulses were being grown, alongside perennial fruits. The simultaneous cultivation of rice, millet, pulses and *Coccinia* cf. *grandis* is an interesting combination. Although the millets may have been grown as maslin crops (cf. de Wet et al. 1983b), *Echinochloa* sp. and rice are likely to have been grown separately, as are *Macrotyloma* cf. *uniflorum*, *Vigna mungo* and *V. acconitifolia*. The *Coccinia* cf. *grandis* will also have needed land to grow prostrate and not strangle other crops. Such land is commonly found at the side of fields or in marginal spaces near houses, although *Coccinia* cf. *grandis* can also be gathered as an opportunistic cultivar or even from the wild. The presence of *Coccinia* cf. *grandis* suggests that people may have been trying to use as much space as possible to extract maximal food potential from their land, or that they were expanding their range of food choices to include things previously considered weeds. The combination of these crops suggests that each of the major summer crop types may have been grown as a mono-crop in a separate parcel of land. The continued use of some sort of ard or plough is possible.

#### Masudpur I

The site of Masudpur I is a six-hectare town-sized settlement situated 13 km from Rakhigarhi, and was occupied in the Mature Harappan period, with some occupation potentially dating immediately before the transition to the post-urban Late Harappan phase (Petrie et al. 2009, 2016; Fig. 4). A range of cereals, pulses and fruits were attested at Masudpur I, but the archaeobotanical assemblage is different from that seen at Masudpur VII. Masudpur I had the greatest range of crops of any site (Table S3; Bates in prep). Of these, summer crops made up 66.12%, while winter crops formed only 28.87%, in contrast to the proportions seen at Mature Harappan Masudpur VII, only around five kilometres away. There were very few tree/orchard crops (0.6%), and a small proportion of unknown-seasonality crops (4.39%) (Table S19). There appears to have been a focus on barley, with a little wheat in the winter season, and in the summer season a mixture of *Echinochloa* sp., *Setaria* sp., and *Panicum* sp. grown alongside rice, all in similar proportions. Various pulses (e.g. *Vigna* sp., *Macrotyloma* cf. *uniflorum, Pisum* sp.*, Cicer* sp., *Lathyrus* sp., *Lens* cf. *culinaris*), fruits, oilseeds (e.g. *Brassica* sp., *Linum usitatissimum, Sesamum* sp., *Indigofera* sp.) and even a gourd (*Coccinia* cf. *grandis*) were present, but only in small quantities (Table S19).

Unlike Burj and Masudpur VII, winter weeds were attested at Masudpur I, along with summer weeds (Table S20). The winter weeds (Table S21) indicate a moist soil preference, though high drought tolerance could suggest reliance on winter rains rather than floodwaters, as there is no evidence for irrigation in the weed ecology. The weeds provide no indication of soil texture, but the alkali preference and the moderate soil fertility suggests leaching was not a problem. The ratio of Fabaceae to Chenopodiaceae weeds is non-season-specific, and suggests that soils were slightly more deficient in nitrogen than not (see Stevens 1996), which meets the expectation for the area around the site (Table 3). The majority of the weeds were biennial, but reproduction was solely through seed bank, which suggests high soil disturbance, and indicates possible plough tillage for winter crops. Some of this winter weed ecology fits well with the predominance of *Hordeum vulgare*, which can survive in drought conditions but prefers soils that are not overly dry, moderate soil fertility, and has no particular soil texture preferences. The high alkali content of the soil is not particularly suited to barley, which generally prefers slightly alkaline to acidic conditions, but given that weeds cannot reliably characterise how alkaline soils are, the high alkali indicators may indeed fit with the barley preferences.

The summer weeds show a different pattern (Table S22), with indications that the soil was dry, and some evidence for drought conditions. The presence of both dry-preference and drought-tolerant weeds indicates poor water management or fit with the distance from the closest river course, though summer flooding from rainfall is likely in this area (Table 3). A slight preference for sandy soils indicated amongst the summer weeds could suggest some potential for leaching, but the moderate fertility and alkaline pH measures argue against this and fit with the expected soil types around the site (Table 3), and also suggest a lack of extensive soil disturbance (Stevens 1996). The ratio of Fabaceae to Chenopodiaceae weeds is non-season-specific, and suggests that soils were generally deficient in nitrogen, which meets the expectation for soils around Masudpur I (Table 3; also see Stevens 1996). Significantly, the majority of weeds are annuals that reproduce through seed bank. Their presence suggests that heavy soil disturbance may have occurred, and their high proportions may indicate plough tillage, which was not seen at the other settlements.

The summer weeds fit well with the main crop choices at Masudpur I, particularly the millets, *Setaria verticillata* and *Echinochloa colona*, and rice, *Oryza* sp. As explored above, millets are very flexible in their ecologies, and the lack of water management indicated by the weeds fits well with these millet species, as does the moderate soil fertility and the range of soil textures. The rice ecology is, however, particularly interesting in relation to the debates surrounding rice agriculture in the Indus Civilisation (Bates et al. 2017a; Petrie et al. 2016). The drier summer conditions suggested by the analysis of the weeds at Masudpur I would suggest that the main cropping of *Oryza* sp. at the site was of a native, non-paddy rice species, perhaps a proto-indica (Bates et al. 2017a; Petrie et al. 2016; see Fuller, 2002, 2006, 2011). The ecological preferences of *Oryza indica* bear many similarities to the conditions indicated in the weed ecologies of Masudpur I:. dry-moist soils, but no flood or drought tolerance; a wide range of soil textures; and no preference regarding soil pH, as the roots are capable of breaking up heavy soils to encourage leaching to remove acids; moderate–low fertility; and a well-prepared seed bed, often produced through plough tillage (Table S1).

The proportions of crops attested in the Mature Harappan phase at Masudpur I thus suggest a sequential multi-cropping strategy, dominated by a cereal duo-culture, with a likelihood of the mono-cropping of wheat and barley in winter and rice and millet in summer (see Galinato et al. 1999). Interestingly, the millets might have been grown as a maslin crop (as they are typically recovered together), though this could also reflect taphonomy rather than cropping practices—as it might at all sites. Various pulses, fruits and oilseeds appear in small quantities, suggesting that they played a minimal role in the cropping system, but the presence of *Linum usitatissimum* is notable because it requires well-watered soil, so might only have been intercropped with *Triticum* sp. if not grown on separate land. Discrete parcels of land were likely required to grow *Pisum* sp., and are likely to have been set aside for *V. acconitifolia*, *V. trilobata*, *Coccinia* cf. *grandis* and potentially *Macrotyloma* cf. *uniflorum*, *Vigna radiata* and *Vigna mungo*. In contrast, *Lens* cf. *culinaris*, *Cicer* sp., and *Lathyrus* sp. could have been intercropped with the winter cereals, or with each other, and *Brassica* sp. and *Sesamum* sp. could have been intercropped with the summer cereals, the latter only with millet. As at Masudpur VII, the likelihood of some use of a plough is notable, though at Masudpur I heavier soil disturbance is likely, potentially indicating plough tillage.

#### Bahola

Bahola is a one-hectare site situated in modern-day Karnal District, Haryana, and has evidence of Late Harappan and PGW period occupation (Singh et al. 2012a, 2013a; Fig. 4). The crop assemblages from these phases are dominated by cereals and include both pulses and fruits, but they are distinct from those seen at the other *Land, Water and Settlement* sites, and also from each other (Tables S3, S23).

In the Late Harappan period there was a clear focus on summer crops: they formed 88.24% of the crop assemblage, which is similar to PGW period Burj and Phase C/D at Rojdi. Only 6.87% of the crop assemblage was identified as winter species; 0.46% was tree/orchard fruit; and 4.43% could not be seasonally assigned. Within the summer portion of the crops, a mix of *Oryza* sp., *Echinochloa* sp. and *Setaria* sp. was commonly observed, and there was also a high proportion of indeterminate small millets (Table S23). *Hordeum vulgare* and indeterminate *Hordeum/Triticum* were the main winter crops, but were relatively minor components of the overall crop assemblage. Various pulses were present, including *Vigna* sp., *Macrotyloma* cf. *uniflorum*, but no winter pulses were identified, although indeterminate Fabaceae were noted (Table S23).

Both winter and summer weeds were found in the assemblage from the Late Harappan levels at Bahola (Table S24). The ecological preferences of the winter weed assemblage suggest that although the soils were moist, drought-tolerant weeds predominated, potentially indicating a lack of water management and the potential for very dry situations (Table S25), which fits with Bahola’s isolation from a perennial water source. The weed soil texture preferences give little indication of the porosity of the soil or the potential for leaching, but do suggest that the soils were generally fertile, and the non-season-specific Chenopodiaceae to Fabaceae weed ratios showed high Chenopodiaceae proportions, suggesting good nitrogen levels, and thus potentially manuring. Soil pH can be modified towards acidic levels through manuring and this interpretation is supported by the high acidic-preference weed proportion, in an area that is mainly alkaline in its soil conditions (Table 3). The two weed species, *Chenopodium* cf. *album* and *Rumex* sp. are also both known to survive in the gut and are commonly found in manure. The high proportion of annuals and seed bank reproducers, together with high nitrogen, therefore suggests that manuring and plough tillage may have been utilised, and the limited land-types exploited could be indicative of intensive small-scale farming.

This contrasts with the ecological preferences of the summer weeds (Table S26), where moist soils, perhaps slightly wetter than those in the winter, and drought-tolerant plants dominate, with more flood-tolerant species in evidence. These data could suggest marginally less control over the water/moisture conditions than in the winter seasons, though given the prevalence of fast moving floods in the summer season in the area today it could in fact represent very careful water management to prevent devastation. The high proportion of sandy-soil weeds could suggest leaching. This is supported somewhat by the high proportion of acidophiles in an area that is predominantly alkaline (Tables 3 and S26), but looking at the weed soil fertility measures it seems that the soil was moderately fertile (more than expected for the area), and the high proportion of Chenopodiaceae mentioned above also suggests high nitrogen values. These data suggest that rather than leaching, the high proportion of acidophiles might again be due to manuring. The weed life cycles also differ from the winter season, as the majority of summer weeds are perennials. Although some annuals were present, vegetative spread was the main method of reproducing beyond one year, and there were also a fairly high proportion of seed-bank reproducers, suggesting that while there may have been some soil disturbance, it was minimal compared with the winter season. This seems to match the suggestion that the range of land exploited was greater than in the winter season, as it encompassed wetlands as well as wastelands, grassland/pastures and arable land.

Interestingly, the weed proportions (Table S24) and weed ecology (Tables S25–26) indicate that the most intensive agriculture was during the winter season, and although it is not visible in the crop assemblage, wheat potentially played a larger role in the winter cropping regime than barley. Barley does not require highly fertile soils or extremely well-prepared seed beds, whereas wheat has high fertility requirements, is slightly more tolerant of acidic conditions, and likes an extremely well-prepared seed bed (Table S1), all of which are suggested by the winter season weeds (Table S25). The main summer crops were *Echinochloa colona*, *Setaria pumila*, and *Oryza* sp., which fit well with the Bahola summer weed ecology. These millets are very adaptable, surviving in wetter soils, during dry periods, growing in poor soils and well-fertilised conditions, and also in a range of soil textures and pH. The rice, although preferring moderately fertilised fields, can also survive in highly fertilised fields, and, like the millets, does not require a well-prepared seed bed as it can break up the soil itself. Similarly, highly acidic conditions are not a problem, as the roots can encourage leaching to make conditions more favourable. This all fits with the less intensive soil disturbance noted in the summer weed ecologies.

These Late Harappan crop proportions from Bahola thus indicate the existence of a summer-dominated sequential multi-cropping strategy potentially involving mono-cropping of rice and a mixed intercrop of millets in summer, and mono-cropping of barley and indeterminate *Hordeum/Triticum*, perhaps wheat, in winter. The winter oilseed is potentially annual or perennial (*Brassica* sp.) (Tables 1, S24), but could have been grown as a strip intercrop with the winter cereals. The summer pulses include both annuals and potential perennials. However, the species that are often grown as mono-crops, either because of their growth habits (spreading, prostrate or shrubby nature) or because of their labour needs, are likely to have been grown in distinct plots of land as additional mono-crops (e.g. *Vigna radiata* and *Macrotyloma uniflorum*), while others might have been row- or strip-intercropped (e.g. *Vigna mungo* and *Vigna trilobata*, with millet or each other). Furthermore, the presence of *Coccinia* cf. *grandis* suggests exploitation of field edges for food growth, similar to the pattern seen in the Late Harappan at Masudpur VII, but also at Masudpur I in the Mature Harappan period. The pattern of the low proportions of *Coccinia* cf. *grandis* at sites where it is present indicates that the high proportion of *Coccinia* cf. *grandis* seeds seen at Masudpur VII in the Late Harappan is potentially due to sample size rather than to grand-scale changes in dietary or agricultural strategy relating to famine and climate change.

The PGW period at Bahola shows some increase in the proportion of winter crops (17.29%) and a decrease in summer crops (72.88%) (Table S23), perhaps suggesting a more balanced approach to sequential multi-cropping in this phase. Tree/orchard fruits also increased in proportion to 4.81%, and a small increase in the proportion of unknown-seasonality crops was also seen (5.02% of the crop assemblage) (Table S23). The most commonly occurring crops were again *Oryza* sp., *Echinochloa* sp., and *Setaria* sp. (Table S23), all of which commonly occur together in contexts (Bates 2016, table 7.11). Indeterminate *Hordeum/Triticum* and *Ziziphus mauritiana* appeared in increased proportions, and although fewer pulse species were attested, the winter crop lentil was found, as well as indeterminate Fabaceae, and small quantities of *Indigofera* sp., a dye crop, rather than the food crop of *Coccinia* cf. *grandis* (Table S23).

The PGW period weeds from Bahola were distinct from those recovered from the Late Harappan period contexts (Table S27). *Chenopodium* cf. *album* was present and is the only possible winter season weed, although it does grow across the seasons. The summer weeds suggest the exploitation of a range of soil moistures, though dry- and drought-tolerant species dominate, suggesting drier rather than wetter conditions (Table S28). These data potentially show similar patterns to the summer weeds of the Late Harappan period at Bahola, suggesting control of water to prevent flooding in the summer months. There is no clear preference as regards soil texture, though some sand preference was noted. Fertility was moderate, but the nitrogen was low according the non-season-specific Fabaceae to Chenopodiaceae weed ratio, and there was also an alkaline preference. All of this is in keeping with the local soil fertility conditions (Table 3). It appears that there was little leaching and also low disturbance of soils, which is supported in the life cycle of the weeds, which shows a combination of perennial and annual varieties, and seed bank and vegetative reproduction. Taken as a whole, the weed ecology indicates that agricultural practices were less intensive in the PGW period than in the Late Harappan period.

Like that of the Late Harappan period, however, the PGW period cropping strategy was summer dominated. It involved mono-cropping of rice and a mixed intercrop of millets in summer, and mono-cropping of barley/wheat in winter, along with the growing of summer pulses and perennial fruit, some of which will have required discrete parcels of land, as in the Late Harappan period, although *Vigna mungo* and *V. trilobata*, were not present. Lentils, a winter pulse, could be grown as a strip intercrop with the winter cereals. *Indigofera* sp. was present, though its precise growing conditions are difficult to characterise. It can be grown as an intercrop (Table S3) (although with which species is unclear), and as a potential biennial or even perennial it might have required land allocation over multiple years.

## Mono-cropping, Intercropping and Sequential Multi-cropping

Archaeobotanical research has developed to the point where a variety of methods can now be used singly or in combination to reconstruct the ways in which human populations managed crops (e.g. ethno-archaeology, macro- and microscopic archaeobotanical analysis, weed ecology, and stable isotope analysis). While these approaches lend themselves to research on a range of socio-economic and political themes, we argue that further research on Indus (and other) assemblages will benefit from more nuanced interpretation of the evidence provided by the combinations of crop seeds and weeds present in specific contexts and phases of occupation. This is because different crop and weed seeds have different ecological requirements, so the presence of specific crops and weeds and the combinations in which they appear provide insight into their growing conditions and hence the cropping practices that local farmers used in each context to produce them. Ellenberg (1950) and van der Veen (1992) have argued that the type of crop can also influence the associated weed flora through features such as leaf shade and rhythm of growth, so while it is necessary to reconstruct local weed ecologies, considerations of change also need to be incorporated. This observation is relevant both to the Indus context that has been explored here in some detail, and more broadly. By revisiting an established terminology and ascertaining which crops can be grown together, which crops can grow in a complementary fashion, and which must be kept apart, it is possible to hypothesise about the strategies used to grow particular assemblages of crops. Establishing how specific crops were grown in the past and whether as mono-crops, intercrops or sequential multi-crops, adds clarity and specificity to our understanding of cropping practices, and makes it possible to coherently characterise diversity and variation across both space and time. While such an approach will be informative more broadly when considering issues of adaptation, intensification and resilience, it comes into its own when used to disaggregate the topic of ‘multi-cropping’, particularly as it has been conceived in the Indus context.

### Environmental Variation, and Diversity in Indus Cropping Practices

We have argued here that the level of environmental variation across the zone occupied by Indus populations, and the diversity of responses to that variation, mean that nuanced characterisations of cropping strategies are essential for understanding the extent of variation in practice. This is particularly relevant when considering issues of adaptation and intensification (Bates in prep.), and response to and resilience in the face of environmental variation and change (see Petrie et al. 2017). While the term ‘multi-cropping’ has been has been used for some time in the Indus context, limited attention to the details of cropping practices has meant that nuanced and interesting variation in the patterns of cropping and ‘multi-cropping’ strategy within and between areas has been masked.

**Fig. 5:**
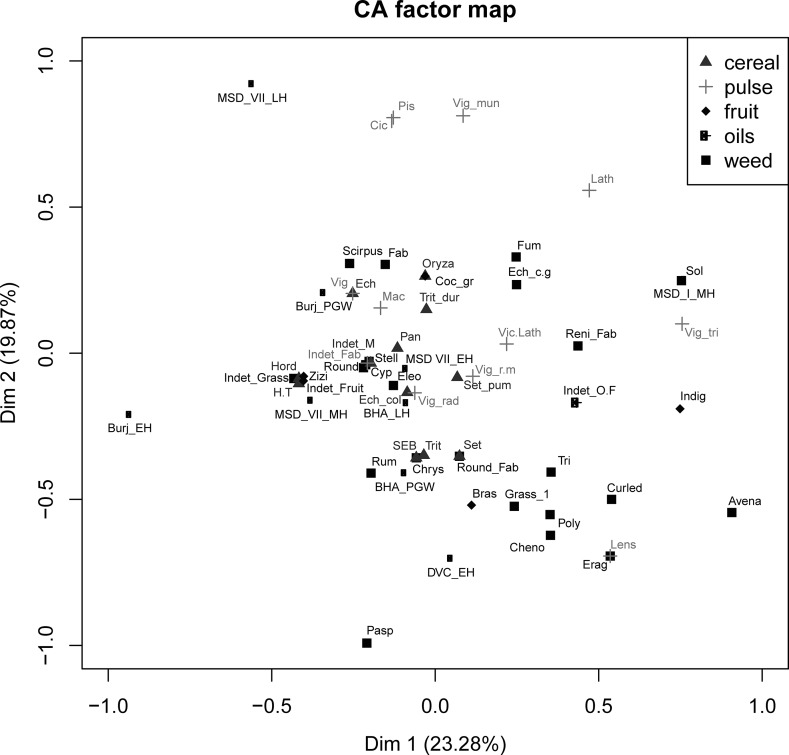
Results of the correspondence analysis that illustrates the relationship between crops, fruits and weeds as they appear at each of the sites investigated by the *Land, Water and Settlement project*

Thus far, Indus ‘multi-cropping’ has been inferred from archaeological assemblages on the basis of the seasonality of particular crops present in specific samples and/or periods of occupation, and contextual associations, such as crops mixed in storage bins (e.g. Weber 1989, 2003). However, there has been limited consideration of how specific crops were grown, and how combinations of winter and summer crops might have been grown in relation to one another within and across seasons. The approach adopted here has considered a combination of crop and weed ecology and modern ethnographic/agricultural data on intercropping potentials to explore the data from Harappa, Rojdi, Babar Kot and the *Land, Water and Settlement* project, and suggests that cropping strategies varied significantly from period to period and site to site. The degree of variation is highlighted by a correspondence analysis of the presence and absence of specific crop and weed seeds at all of the *Land, Water and Settlement* project sites (Fig. 5). This plot shows the clustering of some crop and weed species, and while groups of pulses (e.g. *Pisum*, *Cicer*, *Vigna mungo*) and weeds cluster together, the general pattern is one of variation across both space and time. For example, the presence/absence of crop and weed species, and hence the cropping strategies used, at the Early, Mature and Late Harappan phase occupations at Masudpur VII are distinct and each phase thus appears in different parts of the plot (see above, Table 4). The presence/absence of crop and weed species in the Mature Harappan phases at Dabli vas Chugta, Masudpur VII and Masudpur I are similarly distinct and appear in different parts of the plot (see above, also Table 4).

**Table 4:** Summary of the cropping strategies at Indus sites discussed in this paper

Period	Region	Site	Data	Cropping strategy		
				Winter (main species)	Summer (main species)	Overall
E	W Punjab	Harappa	Crop seeds	*Mono*-*cropping* of barley and wheat with possible pulse *intercropping*	*Mono*-*cropping* of millet	Some *sequential multi*-*cropping* involving (dominant) winter *mono*-*crops*; (minor) summer *mono*-*crops*, with potential *intercropping* of pulses and fruits
M	W Punjab	Harappa	Crop seeds	*Mono*-*cropping* of wheat and barley with possible pulse *intercropping*	*Mono*-*cropping* of millet with possible *intercropping* or *mono*-*cropping* of pulses	Some *sequential multi*-*cropping* involving (dominant) winter *mono*-*crops*; (minor) summer *mono*-*crops*, with potential *intercropping* of pulses and fruits, and *mono*-*crop* of flax
L	W Punjab	Harappa	Crop seeds	*Mono*-*cropping* of barley and wheat with possible pulse *intercropping*	*Mono*-*cropping* of millet and rice(?) with possible *intercropping* of pulses	*Sequential multi*-*cropping* involving (dominant) winter *mono*-*crops*; (minor/supplementary) summer *mono*-*crops*, with potential *intercropping* of pulses and fruits, and *mono*-*crop* of flax
E/A	Gujarat	Rojdi	Crop seeds, weed seeds*	*Mono*-*cropping* of barley	Possible *intercropping* of *Eleusine* sp. and *Panicum miliare*	Some *sequential multi*-*cropping* involving (dominant) summer; (trace/possibly non-local) winter *mono*-*crop,* with intercropping of fruit (*Ziziphus*)
M/B	Gujarat	Rojdi	Crop seeds, weed seeds*	*Mono*-*cropping* of Brassica (mustard)	*Mono*-*cropping* of *Panicum miliare*	Some *sequential multi*-*cropping* involving (dominant) summer *mono*-*crops*; (trace/possibly non-local) winter *mono*-*crop,* with *intercropping* of fruit (*Ziziphus*)
L/C	Gujarat	Rojdi	Crop seeds, weed seeds*	Possible *intercropping* of lentil, *Brassica* (mustard), *Lathyrus* sp. and *Vicia* sp.	*Mono*-*cropping* of *Setaria* cf. *glauca* and *Setaria italica*	Some *sequential multi*-*cropping* involving (dominant) summer cereal *mono*-*crops*; (trace/possibly non-local) winter *intercropping* of cereals and pulses, and *intercropping* of fruit (*Ziziphus*) and pulses
L/C/D	Gujarat	Rojdi	Crop seeds, weed seeds*	Possible *intercropping* of barley, *Lathyrus* sp. and *Vicia* sp.	*Intercropping* of *Eleusine, Panicum miliare* and *Setaria* sp.	Sequential *multi*-*cropping* involving (dominant) summer *intercropped* cereals; (minor/possibly non-local) winter *intercropped* cereals, fruit (*Ziziphus*) and pulses
IM/I	Gujarat	Babar Kot	Crop seeds, weed seeds*	*Mono*-*crop* of lentil	*Mono*-*cropping* of *Panicum miliare*	Very limited *sequential multi*-*cropping* involving (dominant) summer *mono*-*crop*; (trace/minor) winter *mono*-*crop*
L/II	Gujarat	Babar Kot	Crop seeds, weed seeds*	*Mono*-*crop* or *intercropping* of lentil and *Lathyrus* sp. and *mono*-*crop* flax	*Mono*-*cropping* of *Setaria italica* and *Panicum miliare*	Very limited *sequential multi*-*cropping* involving (dominant) summer *mono*-*crop*; (trace) winter *mono*-*crop* or *intercropping*
L/III	Gujarat	Babar Kot	Crop seeds, weed seeds*	*Mono*-*crop* or *intercropping* of mustard, lentil and *Vicia* sp. and *mono*-*crop* flax	*Mono*-*cropping* of *Setaria italica*	Very limited *sequential multi*-*cropping* involving (dominant) summer *mono*-*crop*; (trace) winter *mono*-*crop* or *intercropping* of mustard and pulses, and a *mono*-*crop* of flax
M	Rajasthan	Dabli vas Chugta	Crop seeds, weed seeds	*Mono*-*cropping* of barley	*Mono*-*crop* or *mixed intercrops* of millets	Some *sequential multi*-*cropping* involving (dominant) winter *mono*-*crops* of barley (and wheat), potentially *intercropped* with pulses/mustard; (supplementary) summer *mono*-*crop* of millets, *intercropped* with fruit
E	Haryana	Burj	Crop seeds, weed seeds	*Mono*-*cropping* of barley, *intercropping* of Zizyphus		Winter only (dominant) *mono*-*crop* of barley and possible *intercropping* with fruit
PGW	Haryana	Burj	Crop seeds, weed seeds	*Mono*-*cropping* of barley	*Mixed intercropping* of millets	Some *sequential multi*-*cropping* involving (dominant) summer millets with *intercropped* tropical pulses, and *strip intercropping* or *mono*-*cropping* of mung bean; (supplementary/minor) winter *mono*-*crop* of barley, possibly *intercropped* with winter pulses. Also *mono*-*cropping* or *strip intercropping* of pea and *intercropping* of fruit
E	Haryana	Masudpur VII	Crop seeds, weed seeds	*Mono*-*cropping* of barley and some wheat	*Intercropping* of millet	*Sequential multi*-*cropping* involving (dominant) summer *mixed intercropped* millets, possible *intercropping* of pulses and *strip intercropping* or *mono*-*cropping* of horsegram and *Coccinia grandis*, also *mono*-*cropping* of rice; (supplementary) winter *mono*-*crop* of barley and some wheat with possible *intercropping* of pulses and some *strip intercropping* or *mono*-*cropping* of pea. *Intercropping* of fruit
M	Haryana	Masudpur VII	Crop seeds, weed seeds	*Mono*-*cropping* of wheat and some barley	*Mono*-*crop* of sawa millet	*Sequential multi*-*cropping* involving (dominant) winter *mono*-*crops* of wheat and some barley with possible *intercropping* of pulses and mustard; (supplementary) summer *mono*-*crop* of sawa millet with possible *intercropping* of pulses and *strip intercropping or mono*-*cropping* of horsegram and mung bean. *Intercropping* of fruit
L	Haryana	Masudpur VII	Crop seeds, weed seeds	*Mono*-*cropping* of barley	*Mono*-*crops* of rice and *mixed intercropping* millets	*Sequential multi*-*cropping* involving (dominant) summer *mono*-*crops* of rice and some *mixed intercropping* of millets with possible *intercropping* of pulses some *strip intercropping* or *mono*-*cropping* of horsegram and *Coccinia grandis*; (supplementary/minor) winter *mono*-*crop* of barley with possible *intercropping* of pulses and some *strip intercropping or mono*-*cropping* of pea. *Intercropping* of fruit
M	Haryana	Masudpur I	Crop seeds, weed seeds	*Mono*-*cropping* of barley and some wheat	*Mono*-*crops* of rice and *mixed intercropping* of millets	*Sequential multi*-*cropping* involving (dominant) summer *mono*-*crops* of rice and some *mixed intercropping* of millets with possible *intercropping* of pulses; some *strip intercropping* or *mono*-*cropping* of horsegram, mung bean, sesame, *Indigofera* sp. and *Coccinia grandis*; (supplementary) winter *mono*-*crop* of barley and some wheat with possible *intercropping* of pulses and mustard and some *strip intercropping* or *mono*-*cropping* of pea and flax. *Intercropping* of fruit
L	Haryana	Bahola	Crop seeds, weed seeds	*Mono*-*cropping* of barley	*Mono*-*crops* of rice and *mixed intercropping* of millets	*Sequential multi*-*cropping* involving (dominant) summer *mono*-*crops* of rice and separate *mixed intercropping* of millets, possible *intercropping* of pulses and some *strip intercropping* or *mono*-*cropping* of horsegram, mung bean and *Coccinia grandis*; (supplementary/minor) winter *mono*-*crop* of barley. *Intercropping* of fruit
PGW	Haryana	Bahola	Crop seeds, weed seeds	*Mono*-*cropping* of barley and wheat	*Mono*-*crops* of rice and *mixed intercropping* of millets	*Sequential multi*-*cropping* involving (dominant) summer *mono*-*crops* of rice and separate *mixed intercropping* of millets, possible *intercropping* of pulses; some *strip intercropping* or *mono*-*cropping* of horsegram, mung bean and *Indigofera* sp.; (supplementary/minor) winter *mono*-*crop* of barley with possible *intercropping* of pulse and mustard. *Intercropping* of fruit

The strategies used for the main winter and summer species are differentiated, and then the overall strategy is characterised in terms of the ‘dominant’ crop/season, and whether additional crops are ‘supplementary’, ‘minor’ or ‘trace’

* The weed assemblages from Rojdi and Babar Kot have not been analysed in detail here

The conclusions presented here are in many ways speculative, as the full range of relevant data is not available from all of the Indus sites for which we have good quality archaeobotanical data, but they nonetheless constitute an important first step towards a more nuanced and sophisticated understanding of Indus crop management practices. The assessment of the cropping strategies used at the Indus sites that have been discussed here are summarised in Table 4.

Much of the discussion here has avoided direct consideration of the dynamics of water supply. In the Indus context, it has been argued that perennial and ephemeral water courses were exploited for flood inundation when present, and when not, the inhabitants relied on rainfall, small-scale irrigation, well/lift irrigation and also ponds to supply water (Miller 2006, 2015; Petrie 2017; also Weber 1989, pp. 367–369). There is considerable variation in the distribution of Indus settlements in relation to proximity to these water resources, whether they are produced by rainfall or groundwater. Figure 2 displays the spatial relationship between the known Indus settlements, including the location of the relatively small number of Indus urban centres, and the distribution of winter and summer rainfall. It shows that some Indus settlements were situated close to perennial rivers, while others were located close to ephemeral water courses, or in areas that have no obvious ground water supply (Fig. 2; Petrie et al. 2017). The locations of the settlements that are suitable for nuanced discussion of cropping strategies are shown in Fig. 3. The distribution of these settlements makes it clear that we do not presently have the data for a comprehensive discussion of the transformations that might have accompanied the shift to urbanism, or of the diversity and variation in Indus cropping practices. This is particularly true of the potential variation in cropping practices at the different Indus urban centres, which is certainly likely, but cannot yet be demonstrated. The data from the *Land, Water and Settlement* project sites emphasises the importance of considering smaller village sites, and the potential for considerable variation within regions.

At present we lack high-resolution archaeobotanical information from any of the Indus settlements in Sindh, which is situated along the lower course of the Indus river. The excavations at the urban site of Mohenjo-Daro in the early twentieth century only revealed evidence for winter crops, which might suggest that the farmers that provisioned the urban centre practised single-season winter cropping (Petrie 2017; Petrie et al. 2016, 2017; see Weber et al. 2010, p. 72), potentially exploiting inundation flooding caused by summer snow-melt and Indian Summer Monsoon run-off to saturate the soil ready for winter planting (Petrie 2017; see Miller 2006, 2015). This is, however, largely conjecture, and new excavations and sampling are necessary to ascertain the nature of Indus subsistence and cropping in Sindh, and also the potential transformations that accompanied the rise and decline of the urban centre. It is notable, however, that archaeobotanical assemblages from fourth and third millennium BC occupation at Miri Qalat and Sohr Damb, in west and east Baluchistan respectively, demonstrate the predominance of winter crops (Tengberg 1999; Beneke and Neef 2005). It remains to be established whether all of the settlements in Sindh utilised similar approaches to cropping, which can only be demonstrated through systematic archaeobotanical analysis.

The areas of Gujarat which lie to the east of Sindh have almost no access to winter rain, and the subsistence strategies attested at the sites of Rojdi and Babar Kot are perhaps logically almost entirely dominated by crops grown in summer, particularly millets (Weber 1989, 1991; Reddy 1994, 2003). The available evidence suggests that farmers practised single-season summer cropping in Gujarat, in sharp contrast to the picture hypothesised for Sindh (Petrie 2017). Citing the evidence from Rojdi, Weber et al. (2010, p. 73) have indicated that winter crops were also present in Gujarat, but as outlined above, it is more likely that winter crops made a relatively minimal contribution (in terms of relative abundance), though this appears to have increased over time. This impression is also matched by the less resolved archaeobotanical evidence from sites including Surkotada (Vishnu-Mittre 1990) and Kanmer (Pokharia et al. 2011), which both present a wide range of summer crops, though quantification of these assemblages is lacking. We do not as yet have any coherent information about the plant economy and cropping strategies attested at the urban site of Dholavira. Investigations at earlier sites in the region, such as Loteshwar, have emphasised the importance of interdunal areas for local cropping strategies (García-Granero et al. 2016), and it is likely that such contexts continued to be important in the Indus period.

In the areas of greater Punjab that lie to the north of Sindh and Gujarat, the extremes in the availability of water in summer and winter are less pronounced, though there is certainly spatial variation in the quantities of rainfall in either season. Some areas benefit from direct rainfall from both summer and winter rainfall systems, but most do not receive direct winter rain, and the quantity of summer rainfall is variable (Fig. 2). The geomorphology of the river channels of these northern regions is also likely to have been markedly different to the riverine geomorphology in Sindh (see Weber et al. 2010, table 2).

There has been some discussion about the types of cropping that were possible in greater Punjab, with the assumption that summer and winter cropping were both possible and practised (e.g. Weber 1999, 2003; Weber et al. 2010). However, the limited amount of well-resolved data from across this region has made holistic discussions problematic, though there has been a tendency to suggest that the plains of northwest India received more summer rain, and thus may have seen more use of summer crops (Fuller and Madella 2002, p. 354; Madella and Fuller 2006; Fuller and Murphy 2014).

The possibility that there was some variation in practices across greater Punjab intersects with an on-going debate about the date from which Indus populations commonly used winter and summer crops in conjunction (see Petrie et al. 2016). The observations about cropping practices that have been drawn from the *Land, Water and Settlement* project sites, and also from Harappa, have implications for our understanding of the chronology and spatial resolution of ‘multi-cropping’ in the Indus context. Previously it has been argued that, other than in Gujarat, the exploitation of summer crops was not widespread until the very end of the Indus urban phase, and only became common in the period when the Indus urban system transformed into a rural economy from c. 1900 BC (e.g. Meadow 1996; Fuller and Madella 2002; Fuller 2011; Fuller and Murphy 2014; Pokharia et al. 2014). It has long been clear that the archaeobotanical assemblages from Harappa in the western Punjab show the use of summer crops from the earliest phases of occupation (Weber 1999, 2003), though these crops were relatively minor in terms of relative abundance. The archaeobotanical assemblages from the settlements excavated by the *Land, Water and Settlement* project also clearly indicate that early farmers in eastern Punjab/central Haryana engaged in complex cropping strategies from at least the early third millennium BC. However, it is only from Masudpur VII and Masudpur I that direct radiocarbon dates are now available for several of the winter and summer crops from the same site and contexts (Petrie et al. 2016). As shown in Table 4, the data from these two settlements in particular provide explicit evidence for the use of complex and variable sequential multi-cropping strategies involving the complex exploitation of winter and summer crops in the periods before, during and after the Indus urban phase (Bates 2016; Bates et al. 2017a, b, in press; Petrie et al. 2016, 2017). The degree of variation across space and over time seen at these two sites is paralleled by the evidence from Dabli vas Chugta, Burj, and Bahola, which are each situated in different parts of this eastern zone. Interestingly both winter and summer weeds indicate that various land types were being exploited at each of these settlements (e.g. arable, wasteland, grassland/pastures, wetland).

Several interesting patterns in the combinations of crops by season are evident at the *Land, Water and Settlement* project sites, and this has implications for the practice of intercropping. *Echinochloa* sp., *Setaria* sp. and *Panicum* sp. frequently occur together (Table S3), and they may well have been grown as mixed intercrops (see de Wet et al. 1983b). There is, however, little other evidence for mixed intercropping, suggesting that, particularly for the main crops, the focus was on management of individual crops on distinct parcels of land. The crops that fall into this category include wheat (*Triticum* sp.), barley (*Hordeum vulgare*), pea (*Pisum*), and linseed/flax (*Linum*) in winter, and rice (*Oryza* sp.), sawa millet (*Echinochloa colona*), and mung bean (*Vigna radiata*) and horsegram (*Macrotyloma uniflorum*) in summer. The growth and management of the latter two require the allocation of land either annually or perennially. There is the possibility of row intercropping of several species, including for example urad bean *(Vigna mungo)* and sesame *(Sesamum* sp.) in some periods/at some sites, though some combinations of crops, like *Vigna mungo* and rice, are not productive and they are likely to have been kept apart (Table S1). The only identifiable fruit in the macrobotanical remains at all sites in all periods was jujube (*Ziziphus mauritiana*), but as there is no evidence for land allocation for long term growth of other more likely orchard fruit trees, *Ziziphus* trees were probably growing on field edges as part of a wider strategy of exploitation of natural resources. *Coccinia* cf. *grandis*, though not often found in such high proportions, is similarly likely to have been part of this exploitation strategy, as it is also often grown around the edges of fields or on marginal land, either wild or cultivated for additional vegetable matter.

**Fig. 6:**
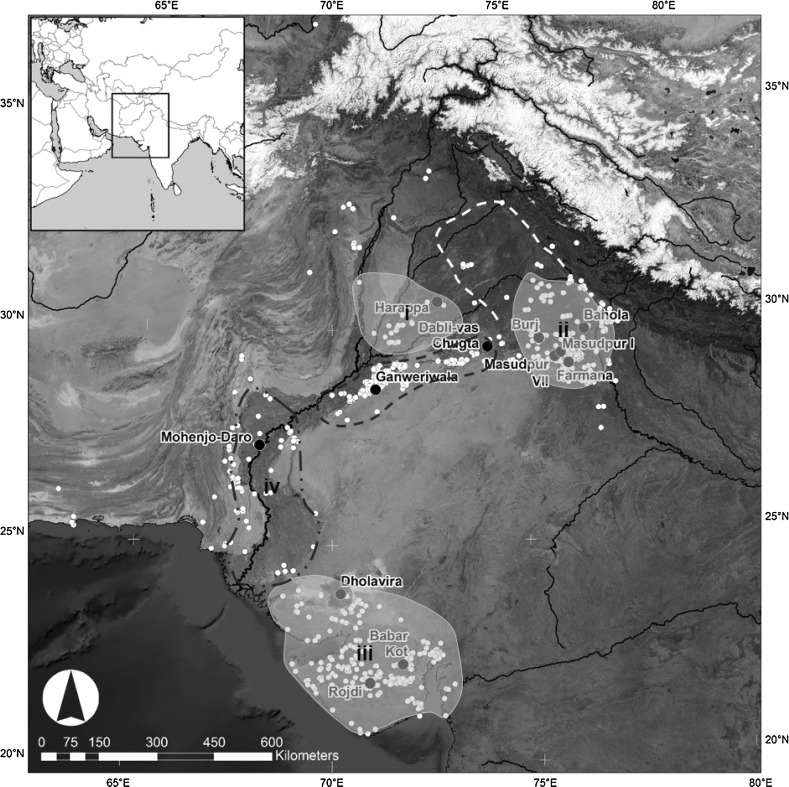
Hypothetical areas within the Indus zone where distinct cropping strategies were practised. Hypothetical areas that are at least partially supported by archaeobotanical data are shown with *solid outlines* and *shading*. Possible areas that lack archaeobotanical data are shown with *dashed lines*

When taken together, the data from all of the *Land, Water and Settlement* project sites clearly demonstrate that there was variation in the proportions of the crops being exploited across space and over time in northwest India (Table 4; Bates 2016; Petrie et al. 2016, 2017). When these data are compared with the evidence from Harappa, it appears that the practices in the more easterly regions were distinct from those in western Punjab, at least as represented at Harappa (Table 4). We hypothesise that the areas of greater Punjab can be divided up into at least two and probably more zones where distinct cropping practices were carried out: the first being the western/central Punjab (Fig. 6i)—as characterised by Harappa—where a degree of sequential multi-cropping was possible, but mono-cropped winter cereals predominated; and the second being the area of the eastern Punjab/Haryana (Fig. 6ii), and potentially also the zone along the base of the Himalaya (*dashed white line* [*green* in online version]), where relatively abundant direct rainfall occurs in both summer and winter. We argue that the climatic and environmental conditions within this latter zone are likely to have made it possible for Indus farmers to grow winter and summer crops in a flexible way, which potentially involved a degree of choice or even innovation in terms of crop selection and particular cropping strategy. It is difficult to be specific about cropping strategies in other areas, because the evidence is simply not available. There has been some discussion about the importance of the Cholistan region as a zone of intensive and extensive cultivation (e.g. Madella 2014), but at present we have no direct evidence for cropping in this zone (Fig. 6, *dashed grey line* [*yellow* in online version]). It lies at the edge of the alluvial plain on the desert margin, and the only potentially analogous assemblage that has been analysed is that from Dabli vas Chugta, in the arid zone of northern Rajasthan at the edge of the Ghaggar palaeochannel (Fig. 6). Geomorphological analysis of the area around Dabli vas Chugta has suggested that it is likely to have benefited from monsoon-induced inundation during the early Mature Harappan periods, and presumably later (Singh et al. 2012a, b; Neogi 2013; Petrie et al. 2017).

The proposed zonation of cropping practices across the area occupied by Indus Civilisation populations is similar to, yet fundamentally distinct from, some of the ‘domains’ or ‘culture-geographic’ regions postulated by Possehl (1982, 1992, 1999, 2002) and Joshi (1984; also Weber et al. 2010). It also complements the suggestion by Petrie et al. (2017) that the variation in practices evident in northwest India indicates that Indus populations in this region engaged in adaptive subsistence strategies to make use of the prevailing winter and summer rainfall in this region, and potentially involved choice and innovation. Further, it supports their suggestion that these adaptive and variable strategies had the potential to be sustainable and resilient, mitigating risk and enabling local populations to cope with varying climatic conditions (see Petrie 2017). Taken together with the evidence for winter-dominated assemblages involving some summer crops in western Punjab (Fig. 6i), the summer-dominated assemblages in Gujarat (Fig. 6iii), and the hypothetical winter-dominated assemblages in Sindh (Fig. 6iv), this evidence suggests considerable diversity in cropping practices across the entire Indus zone (see Petrie 2013; Petrie et al. 2017). It is also likely that there was some diversity in terms of the ‘multi-cropping’ strategies used between these cropping regions, and at least *within* the northeastern area (Petrie et al. 2017). In particular, we argue that there was considerably more ecological and cultural variation across greater Punjab than has been suggested previously (see Petrie et al. 2017). There is some likelihood that adaptive strategies akin to those hypothesised for northwest India (Petrie et al. 2017) and Pakistani Punjab (Weber 2003; Weber et al. 2010) were also ultimately adopted elsewhere within the greater Indus zone, potentially to cope with variable and changing climatic conditions.

## Conclusions

It is hoped that the extended discussion of Indus cropping strategies presented here makes an important contribution to our understanding of ‘multi-cropping’ in the Indus context and more broadly. In terms of cropping strategies, the Indus Civilisation appears to present multiple trajectories to urbanism, with centres in different areas likely to have been more or less reliant on summer and/or winter crops, and there are undoubted connections to the dynamics of direct and indirect water availability (Petrie 2017; Petrie et al. 2017). Although winter-grown wheat and barley were clearly important staple crops in many regions, it has long been clear that summer-grown millets were important in others (Weber 1991), and it is now apparent that locally domesticated rice was also being exploited in northwest India (Bates et al. 2017a; Petrie et al. 2016). This diversity was occurring across an area where populations were simultaneously sharing some categories of material culture and maintaining regional diversity in others (e.g. Wright 2010; Petrie et al. 2016). The Indus Civilisation thus remains a distinctive and compelling case of early complexity that is worthy of much further study.

The conclusions presented here are not intended to be definitive, but to open debate on multi-cropping, ascertain the degree to which it can be characterised archaeologically, and encourage further research on the strategies available to the peoples of the Indus and beyond. We have certainly not exhausted the range of methods that might be utilised, even with our own data. The final publication of the *Land, Water and Settlement* project excavations will incorporate multi-variate statistical analysis focusing on the relationships between crop and weed species at individual sites, and whether strategies change over time. Future analysis by the ERC funded *TwoRains* project will see more comprehensive statistical analysis, combined with the more systematic analysis of new archaeobotanical evidence in conjunction with ethnographic analysis (along the lines of that attempted at various sites/locations in the Mediterranean [see van der Veen 1992; Halstead 2014], and nuanced isotopic analysis attempted in Europe and the ancient Near East [Bogaard et al. 2013, 2015; Fraser et al. 2013; Wallace et al. 2015; Styring et al. 2016]). Such multi-method approaches will facilitate more nuanced characterisations of cropping strategies, creating considerable potential for more focused studies, particularly of adaptation, intensification and resilience.

## Electronic supplementary material

Below is the link to the electronic supplementary material.

Supplementary material 1 (DOCX 230 kb)
