# Supplementary material for: ‘Multi-cropping’, Intercropping and Adaptation to Variable Environments in Indus South Asia
Source: J World Prehist. 2017 May 9;30(2):81–130. doi: 10.1007/s10963-017-9101-z (PMC6991972; doi:10.1007/s10963-017-9101-z)
Supplement: Supplementary file 1 — Supplementary material 1 (DOCX 230 kb) [file 10963_2017_9101_MOESM1_ESM.docx]

Paper submitted to:

*Journal of World Prehistory*

‘Multi-cropping’, Intercropping and Adaptation to Variable Environments in Indus South Asia

SUPPLEMENTARY INFORMATION (SI)

C.A. Petrie* and J. Bates

Department of Archaeology and Anthropology

University of Cambridge

Downing Street, Cambridge, CB2 3DZ

Corresponding author email address: [cap59@cam.ac.uk](mailto:cap59@cam.ac.uk)

# SI.1. Ecological requirements of crop and weed species

Table S1. Ecological requirements of crop species (data obtained from ECOCROP 2016 [find form] website, accessed 15/02/2016)

| ***Crop*** | ***Season*** | ***A/P*** | ***Growth*** | ***Water*** | ***Rainfall***  ***Optimal range***  ***(min-max)*** | ***Salinity*** | ***Soil*** | ***Soil depth*** | ***Fertility*** | ***pH***  ***Optimal range***  ***(min-***  ***max)*** | ***Management*** | ***Companion Crops*** | ***Cycle*** | ***Additional*** |
| --- | --- | --- | --- | --- | --- | --- | --- | --- | --- | --- | --- | --- | --- | --- |
| *Triticum* cf. *aestivum/durum* | R | A | Erect, bright light | Well drained with dry spells but not drought tolerant, requires higher rainfall than other winter cereals. | 750-900  (300-1600)  500-700  (400-800) | Low to medium tolerance (<4 dS/m to 4-10 dS/m) | Medium to heavy to medium texture | Medium (50-150cm) to shallow (20-50cm) | High to moderate | 6-7 (5.5-8.5) | Permanent rain fed mono- cropping  Permanent rain fed ley cropping^[[1]](#footnote-1)^ with high mechanization but low labour intensity |  | 90-250 days  120-180 days |  |
| *Hordeum vulgare* | R | A | Erect, bright light, freely tillering | Well drained with dry spells, tolerant drought and of ‘dry conditions’ due to its precocity (Harlan and Martini 1936). However, does not do well in excessive moisture. | 500-1000  (200-2000) | Low to high tolerance (<4 dS/m to >10 dS/m) | Medium to any soil texture | Deep (>>150cm) to medium (50-150cm) | Moderate to low | 6.5-7.5 (6-8) | Permanent rain fed ley cropping with high mechanization and low intensity | None but suitable for rotation with legumes and pasture | 90-240 days |  |
| *Oryza* sp. (indica used) | K | A | Erect, very bright | Poorly drained, saturated for >50% of year | 1500-2000  (1000-4000) | Low (<4 dS/m) | Any texture; Wide texture generally but dependant on species and management system | Medium (50-150cm) to shallow (20-50cm) | Moderate to low | 5-8 (3.5-9) | Range of management systems dependant on rice species and watering system (see Fuller et al. 2011: Fig. 1; Weisskopf et al. 2014: Fig. 1) |  | 80-200 days | Cannot be grown with *Echinochloa colona* as this is a competitive species (Galinato et al., 1999) |
| *Echinochloa colona* | K | A | Erect but tillering multi-stem and spreads (Galinato et al., 1999), very bright to light shade | Poorly drained, saturated for >50% of the year to well drained with dry spells | 500-1200  (400-2000) | Low to medium tolerance (<4 dS/m to 4-10 dS/m) | Heavy to medium texture | Shallow (20-50cm) | Moderate | 5.5-6.5 (5-7) |  | Can be grown as maslin with various *Setaria* sp. and *Panicum* sp. (de Wet et al., 1983a) | 45-180 days | Cannot be grown with *Oryza* sp. as this is a competitive species (Galinato et al., 1999). |
| *Setaria pumila* (P. sumatrense used in text) | K | A | Erect, very bright | From excessively dry to poorly drained, saturated for >50% of year, both drought and flood tolerant | 350-500  (150-1200) | Low (<4 dS/m) | Medium to light to any soil texture | Medium (50-150cm) to shallow (20-50cm) | Moderate to low | 5.5-6.2 (5-7) |  | Can be grown as maslin with various *Setaria* sp. and *Panicum* sp. (de Wet et al., 1983a) | 75-150 days |  |
| *Panicum* sp.  (P. sumatrense used in text) | K | A | Erect, very bright | From excessively dry to poorly drained, saturated for >50% of year, both drought and flood tolerant | 350-500  (150-1200) | Low (<4 dS/m) | Medium to light to any soil texture | Medium (50-150cm) to shallow (20-50cm) | Moderate to low | 5.5-6.2 (5-7) |  | Can be grown as maslin with various *Setaria* sp. and *Panicum* sp. (de Wet et al., 1983a) | 75-150 days |  |
| *Vigna radiata* | K | A/P | Climber/prostrate, very bright, vine/sub-shrub | Excessively dry to well drained with dry spells, drought resistant, not tolerant to waterlogging | 650-900  (500-1250) | Low to medium tolerance (<4 dS/m to 4-10 dS/m) | Medium to any soil texture | Medium (50-150cm) to shallow (20-50cm) | Moderate to low | 5.5-6.2 (4.3-8.3) |  |  | 50-120 days |  |
| *Vigna mungo* | K | A | Semi-erect/prostrate/procumbent , very bright | Excessively dry to well drained with dry spells, best grown during dry weather with residual soil moisture, drought tolerant | 650-900 (530-2430) | Low to medium tolerance (<4 dS/m to 4-10 dS/m) | Heavy/ medium texture | Deep (>> 150cm) to medium 50-150cm) | High to moderate | 5.5-6.5 (4.5-7.5) |  |  | 60-130 days |  |
| *Vigna aconitifolia* | K | A/P | Semi-erect/prostrate/procumbent, very bright | Excessively dry to well drained with dry spells, intolerant to floods | 500-900 (400-2500) | Low (<4 dS/m) | Light to any texture | Medium (50-150cm) to shallow (20-50cm) | Low | 6.5-7.5 (5-8) | Low management in permanent rain fed systems | Millet and cotton inter-cropping | 60-90 days |  |
| *Vigna trilobata* | K | A/P | Semi-erect/prostrate/procumbent | Well drained with dry spells to poorly drained (including saturated soils for up to 50% of year) | 700-900 (520-1440) | Low to medium tolerance (<4 dS/m to 4-10 dS/m) | Heavy/medium to any texture | shallow (20-50cm) | Moderate to low | 7-8.5 (6.5-9) | Low management (small scale, manual) in grazing system with ‘phase planting’ | *Panicum coloratum, P. maximum, Setaria incrassate, Clitoria ternatea, Desmanthus* sp*. Stylosanthes seabrana* | 30-60 days | Grazing and browsing tolerance, yields of 3 tonnes/ha/yr possible but may be considerably less, can be eaten as veggie but mostly a green manure |
| *Macrotyloma uniflorum* | K | A | Prostrate/procumbent/semi-erect/climber/scrambler/scadent, very light | Well drained with dry spells to excessively dry, drought tolerance, not flood tolerant | 500-1200 (300-4300) | Low to medium tolerance (<4 dS/m to 4-10 dS/m | Medium/light to any texture | Medium (50-150cm) to shallow (20-50cm) | Moderate to low | 5.5-7 (5-8) | Low to medium depending on system ((if permanent rain fed intercropping or ratoon = medium) | Permenant rain fed intercropping with sorghum, maize, niger seed, cotton, *Eleusine*, lentils; permanent rain fed ratoon with rice, sesame; grazing with *Heteropogon* *contortus*, *Panicum maximum, Macroptilium atropurpureum*, *Stylosanthes scabra*; can also be permanent rain fed mono cropping; also arable irrigated mono cropping | 40-180 days | Yields in India vary between 200-900kg/ha |
| *Pisum (sativum* used in text) | R | A | Prostrate/procumbent/semi-erect/climber/scrambler/scadent, very light | Well drained with dry spells | 800-1200 (350-2500) | Low (<4 dS/m) | Any texture | Medium (50-150cm) to shallow (20-50cm) | Moderate to low | 5.5 -7 (4.5-8.3) |  | Arable irrigated ley cropping (rotating crops with legumes and grass pasture) | 60-140 days |  |
| *Cicer* (arietinum used in text) | R | A | Erect, very light | Well drained with dry spells to excessively dry | 600-1000 (300-1800) | Low to medium tolerance (<4 dS/m to 4-10 dS/m | Heavy/ medium to any texture | Medium (50-150cm) to shallow (20-50cm) | Moderate to low | 6-8.5 (4.7-9.5) |  | Arable irrigated ratoon with pearl millet, sorghum, maize, cotton, guar, sesame, rice, jute, durum wheat, wheat, barley, linseed, rapeseed, safflower, tef, faba bean, berseem; arable irrigated intercropping with safflower, sorghum, maize | 90-180 days |  |
| *Lens culinaris* | R | A | Erect, very light | Well drained with dry spells to excessively dry | 600-1000 (250-2500) | Low to medium tolerance (<4 dS/m to 4-10 dS/m | Heavy/medium to any texture | Medium (50-150cm) to shallow (20-50cm) | Moderate to low | 5.5-7.5 (4.5-8.2) |  |  | 70-240 days |  |
| *Lathyrus* (*Sativus* used in text) | R | A | Erect, very light | Well drained with dry spells but also from poorly drained (including saturated for up to 50% of year) to excessively dry | 500-1300 (320-3000) | Low (<4 dS/m) | Any texture | Medium (50-150cm) to shallow (20-50cm) | Moderate to low | 6-7.5 (4.5-8.3) |  |  | 100-190 days | If eaten together with *V. sativa* can have paralytic effects. |
| *Vicia (ervilia* used in text*)* | R | A | Erect/climber or scrambler/scandent, multi-stem | Well drained with dry spells, drought tolerant | 500-700 (300-1200) | Low (<4 dS/m) | Any texture | Medium (50-150cm) to shallow (20-50cm) | High to moderate | 7-7.5 (5.6-8.2) |  |  | 90-150 days |  |
| *Ziziphus mauritiana* | N/A | P | Erect, very light | Well drained with dry spells but also from poorly drained (including saturated for up to 50% of year) to excessively dry, drought tolerant | 300-1500 (130-4000) | Low to medium tolerance (<4 dS/m to 4-10 dS/m Salt sensitive!!!! (better to exploit *Z. nummularia*) | Medium/light, to wide texture | Medium (50-150cm) to shallow (20-50cm) | Moderate to low | 5.5-7.5 (5-8.5) |  |  |  | Takes 6-8 years to start to bear fruit; yield increases after 15-20 years |
| *Brassica (nigra* used in text*)* | R | A/P | Erect | Well drained with dry spells | 600-1400 (300-2500) | Low (<4 dS/m) | Medium to any texture | Medium (50-150cm) to shallow (20-50cm) | Moderate to low | 6 to 7 (5.3 to 7.8) |  |  | 120-180 days | Dry season after emergence of fruit will hamper development of seeds, causing severe loss of yield |
| *Indigofera (tinctoria* used in text *because most greedy)* | K | A/B/P | Erect, very light | Well drained with dry spell | 1300-1700 (640-3000) | Low (<4 dS/m) | Medium to medium and light | Medium (50-150cm) to shallow (20-50cm) | Moderate to low | 6 to 7 (4.3 to 8.7) | Arable irrigated intercropping |  | 90-120 days | Grown on a small scale |
| *Sesamum (indicum* used in text) | K | A | Erect, very light | Well drained with dry spells to excessively dry | 500-1000 (300-1500) | Low (<4 dS/m) | Medium to any texture | Deep (>>150cm) to medium (50-150cm) | Moderate to low | 5.5-7.5 (4.5-8) | Low (ley cropping but also high mechanization) to medium (intercropping) | permanent rainfed intercropping with sorghum, millet, maize, pigeon peas, finger millet |  |  |
| *Linum usitatissimum* | R | A/B/P | Erect, cloudy skies | Well drained with dry spells | 500-800 (250-1300) | Low (<4 dS/m) | Heavy/medium | Shallow (20-50cm) | High | 6-6.5 (5.5-7) |  |  | 80-180 days |  |

Table S2. Ecological requirements of weed species (after Bates 2016, tableVI.1-13)

S2.i: Details by species for water preferences of weeds

| Species | Wet | Dry | Wet/dry | References |
| --- | --- | --- | --- | --- |
| *Chenopodium album* |  |  | X | Holm et al. (1977); Sen (1981) prefer wetter but can grow in dry; Saraswat (1993); |
| *Fumaria sp.* |  |  | X | eFlora (2016) [Fumaria] 'moist'; Murrumbidgee (2016) [Fumitory];Anbg (2016) [Fumaria] does not like flooding |
| *Trianthema triquetra/*  *portulacastrum* | X |  |  | Galinato *et al*. (1999) |
| *Solanum dulcamara* | X |  |  | Fed (2016) [Solanum] |
| *Coix lacrym-jobi* | X |  |  | Proseanet (2016) [Coix];  Pfaf (2016) [Coix] |
| *Chrysopogon* cf. *aciculatus* |  |  | X | FAO (2016) [Chrysopogon] moist but well drained |
| *Echinochloa crus-galli* |  |  | X | Caton et al. (2010); Galinato *et al*. (1999); Holm et al. (1977); Saraswat (1993); Sen (1981) wet/dry, but more often wet; Hooker (1875) wet |
| *Papaver* cf. *rhoeas* |  |  | X | Pfaf (2016) [Papaver] 'moist' |
| *Pennisetum glaucum* |  | X |  | FAO (2016) [Pennisetum];  ECOCROP (2016) [Pennisetum] |
| *Paspalum* sp. – *scrobiculatum* used | X |  |  | ECOCROP (2016) [Paspalum] |
| *Brachiaria* sp. *ramosa* used |  |  | X | Idao (2016) [Brachiaria]; Icunredlist (2016) [Brachiaria]; ECOCROP (2016) [Brachiaria] |
| Wild *Hordeum* (*murinum*) |  |  | X | Johnston et al. (2009) |
| *Aeluropus* sp. *lagopoides* used |  |  | X | Arkive (2016) [Aeluropus]; Tropicos (2016) [Aeluropus] |
| *Eragrostis* sp. |  | X |  | Tropicos (2016) [Eragrostis];  ECOCROP (2016) [Eragrostis] |
| *Medicago/Melilotus/Trifolium* |  |  | X | Pfaf (2016) [Medicago]; Ucanr (2016) [Medicago] |
| *Stellaria* sp. |  |  | X | Fascicles of the Flora of India; Pfaf (2016) [Stellaria];  Ucipm (2016) [Stellaria] |
| *Eleocharis* sp. | X |  |  | Tropicos (2016) [Eleocharis];  Pfaf (2016) [Eleocharis] |
| *Scirpus* sp. | X |  |  | Tropicos (2016) [Scirpus] |
| *Avena* sp. |  | X |  | ECOCROP (2016) [Avena] – well drained |
| Polygonaceae |  |  | X | Tropicos (2016) [Polygonaceae] |
| Cyperaceae |  |  | X | Saraswat (1993) |
| Fabaceae |  |  | X | Tropicos (2016) [Fabaceae] |
| *Rumex* sp. | X |  |  | Tropicos (2016) [Rumex] |

Table S2.ii. Details by species for soil fertility preferences of weeds

|  | Fertile | Interm. | Infertile | References |
| --- | --- | --- | --- | --- |
| *Chenopodium album* | X |  |  | Holm et al. (1977); Sen (1981); Weedecology (2016) [Chenopodium] |
| *Fumaria sp.* |  |  | X | ECOFLORA (2016) [Fumaria] |
| *Trianthema triquetra/*  *portulacastrum* |  | X |  | Galinato *et al*. (1999); Tanveer et al. (2013); Tropicos (2016) [Trianthema] likes saline soil |
| *Solanum dulcamara* | X |  |  | Fed (2016) [Solanum] |
| *Coix lacrym-jobi* | X |  |  | Proseanet (2016) [Coix];  Prota (2016) [Coix]; FAO (2016) [Coix] |
| *Chrysopogon* cf. *aciculatus* |  | X |  | ECOCROP (2016) [Chrysopogon] |
| *Echinochloa crus-galli* | X |  |  | Caton et al. (2010); Galinato et al. (1999); Holm et al. (1977); Sen (1981); Hooker (1875); ECOCROP (2016) [Echinochloa];  Tropicos (2016) [Echinochloa] |
| *Papaver* cf. *rhoeas* | X |  |  | Homeguides (2016) [Papaver] |
| *Pennisetum glaucum* |  | X |  | FAO (2016) [Pennisetum]  well drained; ECOCROP (2016) [Pennisetum] |
| *Paspalum* sp. – *scrobiculatum* used | X |  |  | ECOCROP (2016) [Paspalum] |
| *Brachiaria* sp. *ramosa* used |  | X |  | ECOCROP (2016) [Brachiaria];  Tropicos (2016) [Brachiaria] |
| Wild *Hordeum* (*murinum*) | X |  |  | Archive (2016) [Hordeum] |
| *Aeluropus* sp. *lagopoides* used |  |  | X | Arkive (2016) [Aeluropus]; Tropicos (2016) [Aeluropus] |
| *Eragrostis* sp. |  | X |  | USDA (2016) [Eragrostis] |
| *Medicago/Melilotus/Trifolium* | X | X |  | Pfaf (2016) [Medicago];  Ucanr (2016) [Medicago]; nitrogen fixers |
| *Stellaria* sp. |  | X |  | Pfaf (2016) [Stellaria];  Ucipm (2016) [Stellaria];  likes fertile soils but not necessary |
| *Eleocharis* sp. |  | X |  | USDA (2016) [Eleocharis] |
| *Scirpus* sp. |  | X |  | Tropicos (2016) [Scirpus] |
| *Avena* sp. |  | X |  | ECOCROP (2016) [Avena] – well drained; Sen (1981) |
| Polygonaceae |  | X |  | Tropicos (2016) [Polygonaceae] |
| Cyperaceae |  | X |  | Tropicos (2016) [Cyperaceae] |
| Fabaceae |  | X |  | Tropicos (2016) [Fabaceae]; nitrogen fixers |
| *Rumex* sp. |  | X |  | Stevens (1996) |

Table S2.iii. Details by species for soil texture preferences of weeds

|  | Heavy/  Clay | Loam | Light/ Sandy | References |
| --- | --- | --- | --- | --- |
| *Chenopodium album* | X | X | X | Holm et al. (1977); Hooker (1875) |
| *Fumaria sp.* |  |  |  | No information available |
| *Trianthema triquetra/*  *portulacastrum* |  |  | X | Plantnet (2016) [Trianthema] |
| *Solanum dulcamara* | X | X | X | Fed (2016) [Solanum] |
| *Coix lacrym-jobi* | X | X | X | Pfaf (2016) [Coix] |
| *Chrysopogon* cf. *aciculatus* |  |  | X | Caton et al. (2010); Galinato et al. (1999); ECOCROP (2016) [Chrysopogon] |
| *Echinochloa crus-galli* |  | X | X | Caton et al. (2010); Galinato et al. (1999 |
| *Papaver* cf. *rhoeas* |  | X | X | RHS (2016) [Papaver] |
| *Pennisetum glaucum* |  |  | X | Galinato et al. (1999);  ECOCROP (2016) [Pennisetum] |
| *Paspalum* sp. – *scrobiculatum* used | X | X | X | ECOCROP (2016) [Paspalum] |
| *Brachiaria* sp. *ramosa* used |  |  | X | Galinato et al. (1999);  ECOCROP (2016) [Brachiaria] |
| Wild *Hordeum* (*murinum*) | X |  |  | Archive (2016) [Hordeum] |
| *Aeluropus* sp. *lagopoides* used |  |  | X | Tropicos (2016) [Aeluropus] |
| *Eragrostis* sp. | X | X | X | ECOCROP (2016) [Eragrostis] |
| *Medicago/Melilotus/Trifolium* | X | X | X | Pfaf (2016) [Medicago];  Ucanr (2016) [Medicago] |
| *Stellaria* sp. | X | X | X | Pfaf (2016) [Stellaria];  Ucipm (2016) [Stellaria] |
| *Eleocharis* sp. | X | X | X | Pfaf (2016) [Eleocharis] |
| *Scirpus* sp. | X | X | X | Tropicos (2016) [Scirpus] |
| *Avena* sp. | X | X | X | ECOCROP (2016) [Avena] |
| Polygonaceae | X | X | X | Tropicos (2016) [Polygonaceae] |
| Cyperaceae | X | X | X | Tropicos (2016) [Cyperaceae] |
| Fabaceae | X | X | X | Tropicos (2016) [Fabaceae] |
| *Rumex* sp. | X |  |  | Tropicos (2016) [Rumex] |

Table S2.iv. Details by species for soil pH preferences of weeds

|  | Alkaline (9-15) | Near neutral (6-8) | Acid  (1-5) | References |
| --- | --- | --- | --- | --- |
| *Chenopodium album* | X | X | X | Holm et al. (1977); Hooker (1875) |
| *Fumaria sp.* | X | X |  | Murrumbidgee (2016) [Fumitory_revised]; ECOFLORA (2016) [Fumaria] |
| *Trianthema triquetra/*  *portulacastrum* | X | X |  | Tanveer et al. (2013) |
| *Solanum dulcamara* | X | X | X | Fed (2016) [Solanum] |
| *Coix lacrym-jobi* |  | X | X | Pfaf (2016) [Coix] |
| *Chrysopogon* cf. *aciculatus* |  |  | X | Galinato et al. (1999); ECOCROP (2016) [Chrysopogon] |
| *Echinochloa crus-galli* |  | X |  | ECOCROP (2016) [Echinochloa] |
| *Papaver* cf. *rhoeas* | X | X | X | RHS (2016) [Papaver] |
| *Pennisetum glaucum* |  | X | X | FAO (2016) [Pennisetum];  ECOCROP (2016) [Pennisetum] |
| *Paspalum* sp. – *scrobiculatum* used |  |  | X | ECOCROP (2016) [Paspalum] |
| *Brachiaria* sp. *ramosa* used |  | X |  | Miles et al. (1996);  ECOCROP (2016) [Brachiaria] can tolerate slightly towards the acidic |
| Wild *Hordeum* (*murinum*) |  |  |  | No information available |
| *Aeluropus* sp. *lagopoides* used | X |  |  | Tropicos (2016) [Aeluropus] |
| *Eragrostis* sp. |  | X | X | ECOCROP (2016) [Eragrostis] |
| *Medicago/Melilotus/Trifolium* | X | X |  | Pfaf (2016) [Medicago];  Ucanr (2016) [Medicago]; not acidic |
| *Stellaria* sp. | X | X |  | Pfaf (2016) [Stellaria];  Ucipm (2016) [Stellaria] |
| *Eleocharis* sp. |  | X |  | Pfaf (2016) [Eleocharis]; |
| *Scirpus* sp. | X | X | X | Tropicos (2016) [Scirpus] |
| *Avena* sp. |  | X | X | ECOCROP (2016) [Avena] |
| Polygonaceae | X | X | X | Tropicos (2016) [Polygonaceae] |
| Cyperaceae | X | X | X | Tropicos (2016) [Cyperaceae] |
| Fabaceae | X | X | X | Tropicos (2016) [Fabaceae] |
| *Rumex* sp. |  |  | X | Tropicos (2016) [Rumex] |

Table S2.v. Details by species for flood preferences of weeds

|  | Flood | Neither | Drought | References |
| --- | --- | --- | --- | --- |
| *Chenopodium album* |  |  | X | Weedecology (2016) [Chenopodium] |
| *Fumaria sp.* |  |  | X | Murrumbidgee (2016) [Fumitory_revised] |
| *Trianthema triquetra/*  *portulacastrum* |  | X |  | Galinato et al. (1999) |
| *Solanum dulcamara* |  |  |  | No information available |
| *Coix lacrym-jobi* | X |  |  | FAO (2016) [Coix] |
| *Chrysopogon* cf. *aciculatus* |  |  | X | ECOCROP (2016) [Chrysopogon]; JStor (2016) [Chrysopogon] |
| *Echinochloa crus-galli* | X |  |  | Galinato et al. (1999); ECOCROP (2016) [Echinochloa], can tolerate acidic |
| *Papaver* cf. *rhoeas* |  | X |  | Homeguides (2016) [Papaver] |
| *Pennisetum glaucum* |  |  | X | FAO (2016) [Pennisetum];  ECOCROP (2016) [Pennisetum] |
| *Paspalum* sp. – *scrobiculatum* used | X |  |  | ECOCROP (2016) [Paspalum] |
| *Brachiaria* sp. *ramosa* used |  |  | X | Galinato et al. (1999);  ECOCROP (2016) [Brachiaria] |
| Wild *Hordeum* (*murinum*) |  |  |  | No information available |
| *Aeluropus* sp. *lagopoides* used |  |  | X | Tropicos (2016) [Aeluropus] |
| *Eragrostis* sp. |  | X |  | ECOCROP (2016) [Eragrostis] |
| *Medicago/Melilotus/Trifolium* |  |  | X | Ucanr (2016) [Medicago]; not flood |
| *Stellaria* sp. |  | X |  | Pfaf (2016) [Stellaria];  Ucipm (2016) [Stellaria] |
| *Eleocharis* sp. | X |  |  | Pfaf (2016) [Eleocharis] |
| *Scirpus* sp. | X | X | X | Tropicos (2016) [Scirpus] |
| *Avena* sp. |  |  |  | ECOCROP (2016) [Avena] – well drained |
| Polygonaceae | X | X | X | Tropicos (2016) [Polygonaceae] |
| Cyperaceae | X | X | X | Tropicos (2016) [Cyperaceae] |
| Fabaceae | X | X | X | Tropicos (2016) [Fabaceae] |
| *Rumex* sp. | X | X | X | Tropicos (2016) [Rumex] |

Table S2.vi. Details by species for reproductive cycle of weeds

|  | Perennial | Biennial | Annual | References |
| --- | --- | --- | --- | --- |
| *Chenopodium album* |  |  | Arable, grassland, pasture, wasteland | Holm et al. (1977); Sen (1981); Hooker (1875); Tropicos (2016) [Chenopodium] |
| *Fumaria sp.* |  |  | Arable, wasteland | Hooker (1875); Tropicos (2016) [Fumaria]; Murrumbidgee (2016) [Fumitory_revised] |
| *Trianthema triquetra/*  *portulacastrum* |  |  | Arable, pasture, wasteland | Galinato et al. (1999); Hooker (1875); Tropicos (2016) [Trianthema] |
| *Solanum dulcamara* | Wetland, grassland, woodland |  |  | Fed (2016) [Solanum] |
| *Coix lacrym-jobi* | Arable, grassland, wetland |  | Arable, wetland | Hooker (1875); FAO (2016) [Coix]; Proseanet (2016) [Coix] |
| *Chrysopogon* cf. *aciculatus* | Arable, pasture, grassland, wasteland |  |  | Caton et al. (2010); Galinato et al. (1999); Hooker (1875); ECOCROP (2016) [Chrysopogon]; Indiabiodiversity (2016) [Chrysopogon] |
| *Echinochloa crus-galli* |  |  | Arable, wetland, grassland | Caton et al. (2010); Galinato et al. (1999); |
| *Papaver* cf. *rhoeas* |  |  | Arable, grassland, pasture, wasteland | Hooker (1875);  Kew (2016) [Papaver] |
| *Pennisetum glaucum* |  |  | Arable, grassland, pasture, wasteland | Galinato et al. (1999); Hooker (1875); |
| *Paspalum* sp. – *scrobiculatum* used | Arable, pasture |  | Arable, pasture | Hooker (1875); ECOCROP (2016) [Paspalum] |
| *Brachiaria* sp. *ramosa* used |  |  | Arable, pasture, grassland, wasteland | Galinato et al. (1999); Hooker (1875);  ECOCROP (2016) [Brachiaria]; Tropicos (2016) [Brachiaria]; undisturbed land preferred |
| Wild *Hordeum* (*murinum*) |  |  | Arable, grassland | Hooker (1875); Tropicos (2016) [Hordeum] |
| *Aeluropus* sp. *lagopoides* used | Arable, wasteland |  |  | Tropicos (2016) [Aeluropus];  Kew (2016) [Aeluropus] |
| *Eragrostis* sp. |  |  | Arable, wasteland | Hooker (1875); Tropicos (2016) [Eragrostis]; ECOCROP (2016) [Eragrostis] |
| *Medicago/Melilotus/Trifolium* | Arable, pasture, wasteland | Arable, pasture, wasteland | Arable, pasture, wasteland | Hooker (1875); Ucanr (2016) [Medicago]; not flood |
| *Stellaria* sp. | Grassland, pasture |  | Grassland, pasture | Pfaf (2016) [Stellaria];  Ucipm (2016) [Stellaria] |
| *Eleocharis* sp. | Wetland |  | Wetland | Hooker (1875); Pfaf (2016) [Eleocharis] |
| *Scirpus* sp. |  |  |  | Hooker (1875); Tropicos (2016) [Scirpus] wetland |
| *Avena* sp. | Arable |  | Arable | Holm et al. (1977); Sen (1981); Hooker (1875); ECOCROP (2016) [Avena] – well drained |
| Polygonaceae | Various | Various | Various | Tropicos (2016) [Polygonaceae] |
| Cyperaceae | Various | Various | Various | Tropicos (2016) [Cyperaceae] |
| Fabaceae | Various | Various | Various | Tropicos (2016) [Fabaceae] |
| *Rumex* sp. | Arable, grassland |  |  | Holm et al. (1977); Hooker (1875); Tropicos (2016) [Rumex] |

Table S2vii. Details by species for seasonality of weeds

|  | *Rabi* | *Kharif* | Other | References |
| --- | --- | --- | --- | --- |
| *Chenopodium album* |  |  | X  (Jan.-Sept.) | Hooker (1875); Tropicos (2016) [Chenopodium] |
| *Fumaria sp.* | X | X |  | Hooker (1875) Dec.-March; Tropicos (2016) [Fumaria] March-June; FlowersofIndia (2016) [Fumaria]  April-May; eFlora (2016) [Fumaria] late winter |
| *Trianthema triquetra/*  *portulacastrum* |  | X |  | Hooker (1875); Tropicos (2016) [Trianthema] |
| *Solanum dulcamara* |  | X |  | Hooker (1875); Tropicos (2016) [Solanum] |
| *Coix lacrym-jobi* |  | X |  | Hooker (1875); FAO (2016) [Coix]; Proseanet (2016) [Coix] |
| *Chrysopogon* cf. *aciculatus* |  | X |  | Indiabiodiversity (2016) [Chrysopogon] |
| *Echinochloa crus-galli* |  | X |  | Saraswat (1993) |
| *Papaver* cf. *rhoeas* |  | X |  | Hooker (1875) |
| *Pennisetum glaucum* |  | X |  | FAO (2016) [Pennisetum] |
| *Paspalum* sp. – *scrobiculatum* used |  | X |  | Hooker (1875); ECOCROP (2016) [Paspalum] |
| *Brachiaria* sp. *ramosa* used |  | X |  | Saraswat (1993) |
| Wild *Hordeum* (*murinum*) | X |  |  | Hooker (1875); Tropicos (2016) [Hordeum] |
| *Aeluropus* sp. *lagopoides* used |  | X |  | Tropicos (2016) [Aeluropus] |
| *Eragrostis* sp. |  | X |  | Tropicos (2016) [Eragrostis] |
| *Medicago/Melilotus/Trifolium* | X |  |  | Hooker (1875); Ucanr (2016) [Medicago]; Agriculture-Aajtak (2016) [Medicago]; Irrd (2016) [Medicago] |
| *Stellaria* sp. |  | X |  | Pfaf (2016) [Stellaria];  Ucipm (2016) [Stellaria] |
| *Eleocharis* sp. |  | X |  | Hooker (1875); Pfaf (2016) [Eleocharis] |
| *Scirpus* sp. |  |  |  | No information available |
| *Avena* sp. | X |  |  | Sen (1981); Saraswat (1993); |
| Polygonaceae |  |  |  | Various |
| Cyperaceae |  |  |  | Various |
| Fabaceae |  |  |  | Various |
| *Rumex* sp. | X |  |  | Holm et al. (1977); Hooker (1875); Tropicos (2016) [Rumex] |

Table S2.viii. Details by species for natural habitat of weeds

|  | Natural habitat | References |
| --- | --- | --- |
| *Chenopodium album* | Fields, gardens, ruderal, roadsides, irrigated land, slopes | Hooker (1875); Tropicos (2016) [Chenopodium];  Weedecology (2016) [Chenopodium] |
| *Fumaria sp.* | Wasteland, field borders | Hooker (1875); Tropicos (2016) [Fumaria] |
| *Trianthema triquetra/*  *portulacastrum* | Disturbed land, rocky, hillsides, cultivated areas, wasteland | Hooker (1875); Shetty & Singh (1987); Gonçalves (1978); Tropicos (2016) [Trianthema]; Indiabiodiversity (2016) [Trianthema] |
| *Solanum dulcamara* |  | No information available |
| *Coix lacrym-jobi* | Marshy land | Hooker (1875); FAO (2016) [Coix]; Proseanet (2016) [Coix] |
| *Chrysopogon* cf. *aciculatus* | Abandoned cultivated soil, dry deciduous forests, plains | Indiabiodiversity (2016) [Chrysopogon] |
| *Echinochloa crus-galli* | Swampy areas, ponds | Galinato et al. (1999); ECOCROP (2016) [Echinochloa] |
| *Papaver* cf. *rhoeas* | Arable fields, disturbed land | Wildseed (2016) [Papaver] |
| *Pennisetum glaucum* | Waste places, stubble fields, pastures, meadows | Galinato et al. (1999) |
| *Paspalum* sp. – *scrobiculatum* used | Arable weed, pasture, watery places, swampy places, | ECOCROP (2016) [Paspalum] |
| *Brachiaria* sp. *ramosa* used | Wasteland, undisturbed places, pastures, ditches, cultivated land | Galinato et al. (1999); ECOCROP (2016) [Brachiaria]; Tropicos (2016) [Brachiaria]; undisturbed land preferred |
| Wild *Hordeum* (*murinum*) | Cultivated land only (introduced to region) | Hooker (1875); Tropicos (2016) [Hordeum] |
| *Aeluropus* sp. *lagopoides* used | Salt marshes, wasteland, abandoned cultivated land | Tropicos (2016) [Aeluropus] |
| *Eragrostis* sp. | Wasteland, abandoned cultivated land | Tropicos (2016) [Eragrostis] |
| *Medicago/Melilotus/Trifolium* | Fields, cultivated land, wasteland, disturbed area | Hooker (1875); Ucanr (2016) [Medicago];  Agriculture-Aajtak (2016) [Medicago];  Irrd (2016) [Medicago] |
| *Stellaria* sp. | Disturbed land | Pfaf (2016) [Stellaria];  Ucipm (2016) [Stellaria] |
| *Eleocharis* sp. | Wetlands, marshes, ponds | Pfaf (2016) [Eleocharis] |
| *Scirpus* sp. |  | No information available |
| *Avena* sp. | Cultivated land only (introduced to region) | Sen (1981); Saraswat (1993); |
| Polygonaceae |  | Various |
| Cyperaceae |  | Various |
| Fabaceae |  | Various |
| *Rumex* sp. |  | No information available |

Table S2.ix. Details by species for photosynthetic pathways of weeds

|  | C3 | C4 | References |
| --- | --- | --- | --- |
| *Chenopodium album* | X |  | Weedecology (2016) [Chenopodium] |
| *Fumaria sp.* | X |  | ECOFLORA (2016) [Fumaria] |
| *Trianthema triquetra/*  *portulacastrum* |  | X | Sikolia et al. (2009) |
| *Solanum dulcamara* |  |  | No information available |
| *Coix lacrym-jobi* |  | X | Proseanet (2016) [Coix] |
| *Chrysopogon* cf. *aciculatus* |  | X | Rundell (1980) |
| *Echinochloa crus-galli* |  | X | Galinato et al. (1999) |
| *Papaver* cf. *rhoeas* | X |  | Garćia-Palacios et al. (2011) |
| *Pennisetum glaucum* |  | X | Galinato et al. (1999); FAO (2016) [Pennisetum] |
| *Paspalum* sp. – *scrobiculatum* used |  | X | ECOCROP (2016) [Paspalum] |
| *Brachiaria* sp. *ramosa* used |  | X | ECOCROP (2016) [Brachiaria] |
| Wild *Hordeum* (*murinum*) | X |  |  |
| *Aeluropus* sp. *lagopoides* used |  | X | Waghmode (1979) |
| *Eragrostis* sp. |  | X | Waller & Lewis (1979) |
| *Medicago/Melilotus/Trifolium* | X |  | Garćia-Palacios et al. (2011); Boutton et al. (1980); Kimble et al. (2000) |
| *Stellaria* sp. | X |  | Pfaf (2016) [Stellaria] |
| *Eleocharis* sp. | X |  | Bruhl & Wilson (2007) |
| *Scirpus* sp. |  |  | No information available |
| *Avena* sp. | X |  |  |
| Polygonaceae |  |  | No information available |
| Cyperaceae | X | X | Bruhl & Wilson (2007) |
| Fabaceae |  |  | No information available |
| *Rumex* sp. |  |  | No information available |

Table S2.x. Details by species for reproductive method of weeds

|  | Seed bank | Vegetational spread | Both | References |
| --- | --- | --- | --- | --- |
| *Chenopodium album* | X |  |  | Weedecology (2016) [Chenopodium] |
| *Fumaria sp.* | X |  |  | Murrumbidgee (2016) [Fumitory_revised] |
| *Trianthema triquetra/*  *portulacastrum* |  |  | X | Tanveer et al. (2013); Galinato et al. (1999);  Tropicos (2016) [Trianthema];  Plantnet (2016) [Trianthema] |
| *Solanum dulcamara* |  |  |  | No information available |
| *Coix lacrym-jobi* |  | X |  | Proseanet (2016) [Coix]; FAO (2016) [Chrysopogon] |
| *Chrysopogon* cf. *aciculatus* |  | X |  | ECOCROP (2016) [Chrysopogon] |
| *Echinochloa crus-galli* |  |  | X | Galinato et al. (1999) |
| *Papaver* cf. *rhoeas* | X |  |  | Kew (2016) [Papaver] |
| *Pennisetum glaucum* |  | X |  | FAO (2016) [Pennisetum] |
| *Paspalum* sp. – *scrobiculatum* used |  | X |  | ECOCROP (2016) [Paspalum] |
| *Brachiaria* sp. *ramosa* used |  |  | X | Bhatt & Singh (2007) |
| Wild *Hordeum* (*murinum*) |  |  |  | Neither: seed only, little evidence for seed dormancy  Archive (2016) [Hordeum];  Wric (2016) [Hordeum] |
| *Aeluropus* sp. *lagopoides* used |  | X |  | Gulzar & Khan (2001);  Tropicos (2016) [Aeluropus];  Kew (2016) [Aeluropus] |
| *Eragrostis* sp. | X |  |  | Tropicos (2016) [Eragrostis] |
| *Medicago/Melilotus/Trifolium* | X |  |  | Fed (2016) [Melilotus] |
| *Stellaria* sp. |  |  | X | Ucipm (2016) [Stellaria];  Pfaf (2016) [Stellaria] |
| *Eleocharis* sp. |  |  | X | Pfaf (2016) [Eleocharis] |
| *Scirpus* sp. |  |  |  | No information available |
| *Avena* sp. | X |  |  | ECOCROP (2016) [Avena] – well drained |
| Polygonaceae |  |  | X | Various |
| Cyperaceae |  |  | X | Various |
| Fabaceae |  |  | X | Various |
| *Rumex* sp. |  | (X) |  | Tropicos (2016) [Rumex] statement that can produce rhizomes and vegetation spread but no statement on seed dormancy |

Table S2.xi. Details by species for root system of weeds

|  | Tap root | Rhizome | Both | References |
| --- | --- | --- | --- | --- |
| *Chenopodium album* | X |  |  | Weedecology (2016) [Chenopodium] |
| *Fumaria* sp*.* | X |  |  | Gupta & Rao (2012);  Murrumbidgee (2016) [Fumitory_revised] |
| *Trianthema triquetra/*  *portulacastrum* | X |  |  | Botanicgardens (2016) [Trianthema] |
| *Solanum dulcamara* |  |  |  | No information available |
| *Coix lacrym-jobi* |  |  |  | No information available |
| *Chrysopogon* cf. *aciculatus* |  | X |  | ECOCROP (2016) [Chrysopogon] |
| *Echinochloa crus-galli* |  | X |  | Galinato et al. (1999); ECOCROP (2016) [Echinochloa] |
| *Papaver* cf. *rhoeas* | X |  |  | McNaughton & Harper (1964); ECOFLORA (2016) [Papaver] |
| *Pennisetum glaucum* |  | X |  | FAO (2016) [Pennisetum] |
| *Paspalum* sp. – *scrobiculatum* used |  | X |  | ECOCROP (2016) [Paspalum] |
| *Brachiaria* sp. *ramosa* used |  | X |  | Idao (2016) [Brachiaria]; Icunredlist (2016) [Brachiaria];  ECOCROP (2016) [Brachiaria] |
| Wild *Hordeum* (*murinum*) |  |  | X | Not a deep tap root but not rhizomous in its spread  Archive (2016) [Hordeum] |
| *Aeluropus* sp. *lagopoides* used |  | X |  | Tropicos (2016) [Aeluropus];  Kew (2016) [Aeluropus] |
| *Eragrostis* sp. |  |  | X | Not a deep tap root but not rhizomous in its spread  ECOCROP (2016) [Eragrostis] |
| *Medicago/Melilotus/Trifolium* | X |  |  | Fed (2016) [Melilotus];  Ucanr (2016) [Medicago];  Ibaf (2016) [Medicago] |
| *Stellaria* sp. |  |  | X | Shallow tap root with rhizomes  Ucipm (2016) [Stellaria];  Pfaf (2016) [Stellaria]; |
| *Eleocharis* sp. |  | X |  | Matting  Tropicos (2016) [Eleocharis] |
| *Scirpus* sp. |  |  |  | No information available |
| *Avena* sp. |  |  | X | Not a deep tap root but not rhizomous in its spread  ECOCROP (2016) [Avena] |
| Polygonaceae |  |  | X | Unknown species so assume both types possible |
| Cyperaceae |  |  | X | Unknown species so assume both types possible |
| Fabaceae |  |  | X | Unknown species so assume both types possible |
| *Rumex* sp. |  |  | X | Can produce rhizomes along tap root Tropicos (2016) [Rumex] |

Table S2.xii. Details by species for soil depth of weeds

|  | Shallow | Deep | References |
| --- | --- | --- | --- |
| *Chenopodium album* |  | X | Weedecology (2016) [Chenopodium] |
| *Fumaria sp.* | X |  | Gupta & Rao (2012) |
| *Trianthema triquetra/*  *portulacastrum* |  | X | Hooker (1875) |
| *Solanum dulcamara* |  |  | No information available |
| *Coix lacrym-jobi* |  |  | No information available |
| *Chrysopogon* cf. *aciculatus* | X |  | ECOCROP (2016) [Chrysopogon] |
| *Echinochloa crus-galli* | X |  | Galinato et al. (1999);  ECOCROP (2016) [Echinochloa] |
| *Papaver* cf. *rhoeas* | X |  | ECOFLORA (2016) [Fumaria] |
| *Pennisetum glaucum* |  | X | Deep soil needed  Galinato et al. (1999); FAO (2016) [Pennisetum];  ECOCROP (2016) [Pennisetum];  CAES (2016) [Pennisetum] |
| *Paspalum* sp. – *scrobiculatum* used |  | X | ECOCROP (2016) [Paspalum] |
| *Brachiaria* sp. *ramosa* used | X |  | ECOCROP (2016) [Brachiaria] |
| Wild *Hordeum* (*murinum*) | X |  | Archive (2016) [Hordeum] |
| *Aeluropus* sp. *lagopoides* used | X |  | Tropicos (2016) [Aeluropus] |
| *Eragrostis* sp. | X |  | ECOCROP (2016) [Eragrostis] |
| *Medicago/Melilotus/Trifolium* | X |  | Fed (2016) [Melilotus];  Ucanr (2016) [Medicago] |
| *Stellaria* sp. | X |  | Ucipm (2016) [Stellaria]; Illinoiswildflowers (2016) [Stellaria]; PSU (2016) [Stellaria] |
| *Eleocharis* sp. | X |  | Tropicos (2016) [Eleocharis] |
| *Scirpus* sp. |  |  | No information available |
| *Avena* sp. | X |  | ECOCROP (2016) [Avena] |
| Polygonaceae |  |  | No information available |
| Cyperaceae |  |  | No information available |
| Fabaceae |  |  | No information available |
| *Rumex* sp. | X |  | Tropicos (2016) [Rumex] |

# SI.2. Crop and weed species identified at Land, Water and Settlement sites

*Table S3. Presence/absence of crop species by site (after Bates 2016, table 7.1)*

| Species | Species Code | DVC  (EH) | Burj  (EH) | Burj (PGW) | MSD  VII (EH) | MSD  VII (MH) | MSD  VII (LH) | MSD I  (MH) | BHA (LH) | BHA (PGW) |
| --- | --- | --- | --- | --- | --- | --- | --- | --- | --- | --- |
| *Hordeum* *vulgare* | Hord. | X | X | X | X | X | X | X | X | X |
| *Triticum* sp. | Trit. | X |  | X | X | X |  | X |  | X |
| *Triticum* cf. *durum*/*aestivum* |  |  |  | X | X | X |  | X |  |  |
| *Hordeum/Triticum* | H/T | X | X | X | X | X | X | X | X | X |
| *Oryza* sp. | Oryza |  |  |  | X |  | X | X | X | X |
| *Echinochloa* sp. | Ech. |  |  | X | X | X | X | X | X | X |
| *Echinochloa colona* |  |  |  | X | X | X |  | X | X | X |
| *Setaria* sp. | Set. | X |  | X | X |  |  | X | X | X |
| *Setaria* cf. *pumila* |  |  |  | X | X |  |  | X | X | X |
| *Panicum*  sp. | Pan. | X |  | X | X |  | X | X | X | X |
| SEB^^[[2]](#footnote-2)^^ | SEB | X |  | X | X | X |  | X | X | X |
| Indeterminate small millet | Indet. M. | X |  | X | X | X | X | X | X | X |
| *Vigna* sp. | Vig. |  |  | X | X | X | X | X | X | X |
| *Vigna radiata* | Vig. rad. |  |  | X |  | X |  | X | X | X |
| *Vigna mungo* | Vig. mun. |  |  |  |  |  | X | X | X |  |
| *Vigna radiata/mungo* | Vig. r/m |  |  |  | X | X |  | X | X |  |
| *Vigna acconitifolia* | Vig. acc. |  |  |  |  |  | X | X |  |  |
| *Vigna* cf. *trilobata* | Vig. tri. |  |  |  |  |  |  | X | X |  |
| *Macrotyloma*  cf. *uniflorum* | Mac. |  |  |  | X | X | X | X | X | X |
| *Pisum* sp. | Pis. |  |  | X | X |  | X | X |  |  |
| *Cicer* sp. | Cic. |  |  | X | X |  | X | X |  |  |
| *Lens* cf. *culinaris* | Lens | X |  |  |  |  |  | X |  | X |
| *Lathyrus* sp. | Lath. |  |  | X |  |  |  | X |  |  |
| *Vicia/Lathyrus* | Vic/Lath |  |  |  | X | X |  | X |  |  |
| Indeterminate Fabaceae | Indet. Fab. | X |  | X | X | X | X | X | X | X |
| *Ziziphus mauritiana* | Zizi. | X | X | X | X | X | X | X | X | X |
| Indeterminate Fruit | Indet. Fruit | X | X | X | X | X | X | X | X | X |
| *Brassica* sp. | Bras. | X |  |  | X | X |  | X |  | X |
| cf. *Indigofera* sp. | Indig. |  |  |  |  |  |  | X |  | X |
| *Coccinia* cf. *grandis* | Coc. gr. |  |  |  | X |  | X | X | X | X |
| *Sesamum* sp. | Ses. |  |  |  |  |  |  | X |  |  |
| *Linum* cf. *usitatissimum* | Linum |  |  |  |  |  |  | X |  |  |
| Indeterminate Oilseed/ Fibre | Indet O/F |  |  |  | X |  |  | X |  | X |

Table S4. Presence/absence of weed species by site (after Bates 2016, table 7.2)

| Species | Species Code | DVC (EH) | Burj  (EH) | Burj  (PGW) | MSD  VII  (EH) | MSD VII  (MH) | MSD VII  (LH) | MSD  I  (MH) | BHA  (LH) | BHA  (PGW) |
| --- | --- | --- | --- | --- | --- | --- | --- | --- | --- | --- |
| *Trianthema* *triquetra* | Tri. | X |  |  | X |  |  | X | X |  |
| *Stellaria* sp. | Stell. | X |  | X | X | X | X | X | X | X |
| *Stellaria* cf. *nemorum* | Stell. Nem. |  |  |  |  |  |  | X |  |  |
| *Chenopodium* sp. | Cheno. | X |  |  |  |  |  | X | X | X |
| *Eleocharis* sp. | Eleo. | X |  |  | X | X | X | X | X | X |
| Cf. *Scirpus* sp. | Scirpus |  |  | X | X | X | X | X |  | X |
| Cyperaceae | Cyp. | X |  | X | X | X | X | X | X | X |
| *Acacia* sp. | Aca. |  |  |  |  |  |  | X |  |  |
| Medicago/Melilotus/Trifolium | MMT |  |  |  |  |  |  | X |  |  |
| Small round Fabaceae | Round Fab. | X |  | X | X |  |  | X | X | X |
| Small reniform Fabaceae | Reni. Fab. |  |  |  | X |  |  | X | X |  |
| Small Fabacaeae | Fab. |  |  | X | X |  | X | X | X | X |
| Mimosoideae | Mim. |  |  |  |  |  |  | X |  |  |
| *Fumaria* cf. *officinalis* | Fum. |  |  | X | X |  |  | X |  |  |
| Papaveraceae | Pap. |  |  |  |  |  | X |  |  |  |
| *Aelupurus* sp. | Ael. |  |  |  |  |  |  | X |  |  |
| cf. *Avena* sp. | Avena | X |  |  |  |  |  | X |  |  |
| *Brachiaria* sp. | Brach. |  |  |  |  |  |  | X |  |  |
| cf. *Chrysopogon* sp. | Chrys. | X |  | X | X | X |  | X | X | X |
| *Coix lacryma-jobi* | Coix |  |  |  |  |  |  | X |  |  |
| *Echinochloa crus-galli* | Ech. c-g |  |  | X |  |  |  | X | X |  |
| *Eragrostis* sp. | Erag. | X |  |  |  |  |  | X |  | X |
| cf. *Paspalum* sp. | Pasp. |  |  |  |  |  |  |  |  | X |
| cf. *Pennisetum* sp. | Penn. |  |  | X |  |  | X |  |  |  |
| Indet. Big Millet | Big M. |  |  |  |  |  | X | X |  |  |
| Large grass | Large Grass |  |  |  |  |  |  | X |  |  |
| Indet. Grass Type 1 | Grass 1 | X |  |  | X |  |  | X | X | X |
| Indeterminate Small Grass | Indet. Grass | X | X | X | X | X | X | X | X | X |
| *Rumex* cf. *crispus* | Rum. |  |  |  |  |  |  |  | X |  |
| Polygonaceae | Poly. | X |  |  | X |  |  | X |  | X |
| *Solanum* cf. *dulcamara* | Sol. |  |  |  | X |  |  | X |  |  |
| Indet curled embryo | Curled | X |  |  |  |  |  | X | X |  |
| Indet. small round seed | Round | X |  | X | X | X | X | X | X | X |

# SI.3. Dabli vas Chugta

Table S5. Proportion of crop genera of crop assemblage at Dabli vas Chugta (after Bates 2016, table 9.1)

| Crop Taxa | Proportion of  assemblage | Season |
| --- | --- | --- |
| *Hordeum vulgare* | 34.26% | W |
| *Triticum* sp. | 0.78% | W |
| *Hordeum/Triticum* | 15.70% | W |
| *Setaria* sp. | 0.78% | S |
| *Panicum* sp. | 0.78% | S |
| SEB | 3.89% | S |
| Indet. small millet | 19.47% | S |
| *Lens* cf. *culinaris* | 2.91% | W |
| Indet. Fabaceae | 11.52% | W/S/P |
| *Ziziphus mauritiana* | 2.88% | P |
| Indet. Fruit | 1.73% | P |
| *Brassica* sp. | 5.31% | W |
| Summer crops | 24.91% |  |
| Winter crops | 58.95% |  |
| Tree/orchard | 4.61% |  |
| Unknown | 11.25% |  |

Table S6. Average count per 10l sediment of weed genera at Dabli vas Chugta (after Bates 2016, table 7.4)

| Weed Taxa | Count per 10l | Season |
| --- | --- | --- |
| *Stellaria* sp*.* | 0.01 | S |
| *Eleocharis* | 0.25 | S |
| *Trianthema triquetra* | 0.15 | S |
| *Eragrostis* sp. | 0.01 | S |
| *Chrysopogon* sp. | 0.08 | S |
| *Avena* sp. | 0.03 | W |
| *Chenopodium album* | 0.01 | W/S |
| Cyperaceae | 0.01 | W/S/P |
| Indeterminate grass | 0.39 | W/S/P |
| Small round Fabaceae | 0.03 | W/S/P |
| Indet. curled embryo | 0.08 | W/S/P |
| Round | 0.87 | W/S/P |
| Indet. seed | 0.14 | W/S/P |
| Summer weeds | 92.59% |  |
| Winter weeds | 5.56% |  |
| Both seasons weeds | 1.85% |  |

*Table S7: Relative proportions of summer weeds at Dabli vas Chugta by ecological preference indicators (after Bates 2016, table 10.1)*

| *Water* | *Wet* | | *Moist* | | *Dry* | |
| --- | --- | --- | --- | --- | --- | --- |
|  | 77.99% | | 20.30% | | 1.96% | |
|  |  | |  | |  | |
| *Flood Tolerance* | *Flood* | | *Drought* | | *Neither* | |
|  | 48.58% | | 17.65% | | 34.02% | |
|  |  | |  | |  | |
| *Soil Depth* | *Shallow* | | | *Deep* | | |
|  | 68.87% | | | 31.37% | | |
|  |  | |  | |  | |
| *Root Type* | *Rhizomes* | | *Tap* | | *Both* | |
|  | 66.23% | | 31.37% | | 2.65% | |
|  |  | |  | |  | |
| *Soil Texture* | *Sand* | *Loam* | | *Clay* | | *Any* |
|  | 45% | 0% | | 0% | | 55% |
|  |  | |  | |  | |
| *Soil Fertility* | *Fertile* | | *Between* | | *Infertile* | |
|  | 1.96% | | 98.29% | | 0% | |
|  |  | |  | |  | |
| *Soil pH* | *Acid* | *Alkali* | | *Neutral* | | *Any* |
|  | 17.65% | 32.06% | | 48.58% | | 1.96% |
|  |  | |  | |  | |
| *Reproduction* | *Seed bank* | | *Vegetal Spread* | | *Both* | |
|  | 33.33% | | 15.69% | | 51.23% | |
|  |  | |  | |  | |
| *Reproductive Cycle* | *Annual* | | *Biannual* | | *Perennial* | |
|  | 33.33% | | 51.23% | | 15.69% | |

# SI.4. Burj

*Table S8. Proportion of crop genera of crop assemblage at Burj in the Early Harappan period (after Bates 2016, table 9.3)*

| Crop Genera | | | Proportion of  Assemblage | | Season | |
| --- | --- | --- | --- | --- | --- | --- |
| *Hordeum vulgare* | | | 8.33% | | W | |
| *Hordeum*/*Triticum* | | | 33.33% | | W | |
| *Ziziphus mauritiana* | | | 16.67% | | P | |
| Indet. fruit | | | 41.67% | | P | |
| Summer crops | 0% | |  | |  |  |
| Winter crops | 41.66% | |  | |  |  |
| Tree/Orchard | 58.34% | |  | |  |  |
| Unknown | 0% | |  | |  |  |

*Table S9. Proportion of crop genera of crop assemblage at Burj in the PGW period (after Bates 2016, table 7.6)*

| Crop Genera | | | Proportion of  Assemblage | | Season | |
| --- | --- | --- | --- | --- | --- | --- |
| *Hordeum vulgare* | | | 5.42% | | W | |
| *Triticum* sp. | | | 0.24% | | W | |
| *Hordeum*/*Triticum* | | | 3.30% | | W | |
| *Echinochloa* sp. | | | 42.69% | | S | |
| *Setaria* sp. | | | 23.11% | | S | |
| *Panicum* sp. | | | 3.07% | | S | |
| SEB | | | 5.66% | | S | |
| Indet. small millet | | | 6.84% | | S | |
| *Vigna* sp. | | | 1.65% | | S | |
| *Vigna radiata* | | | 0.24% | | S | |
| *Pisum* sp. | | | 0.47% | | W | |
| *Cicer* sp. | | | 0.24% | | W | |
| *Lathyrus* sp. | | | 0.24% | | W | |
| Indet. Fabaceae | | | 0.94% | | W/S/P | |
| *Ziziphus mauritiana* | | | 2.59% | | P | |
| Indet. fruit | | | 3.30% | | P | |
| Summer crops | 83.25% | |  | |  |  |
| Winter crops | 9.91% | |  | |  |  |
| Tree/Orchard | 5.90% | |  | |  |  |
| Unknown | 0.94% | |  | |  |  |

Table S10. Average count per 10l sediment of weed genera at Burj in the PGW period (after Bates 2016, table 7.6)

| Weed Taxa | Count per 10l | Season |
| --- | --- | --- |
| *Stellaria* sp. | 0.57 | S |
| *Fumaria* cf. *officinalis* | 0.04 | S |
| *Chrysopogon* sp. | 0.04 | S |
| *Echinochloa* *crus-galli* | 0.07 | S |
| *Pennisetum* sp. | 0.07 | S |
| Cyperaceae | 0.38 | W/S/P |
| Fabaceae | 0.11 | W/S/P |
| Indeterminate small grass | 0.32 | W/S/P |
| Round | 0.18 | W/S/P |
| Indet. seed | .04 | W/S/P |
| Summer weeds | 100% |  |
| Winter weeds | 0% |  |
| Both seasons weeds | 0% |  |

*Table S11. Relative proportion of summer* *weeds at Burj in the PGW period by ecological preference indicators (after Bates 2016, table 10.2)*

| *Water* | *Wet* | | *Moist* | | *Dry* | |
| --- | --- | --- | --- | --- | --- | --- |
|  | 0% | | 90.91% | | 9.09% | |
|  |  | |  | |  | |
| *Flood Tolerance* | *Flood* | | *Drought* | | *Neither* | |
|  | 9.09% | | 18.08% | | 72.33% | |
|  |  | |  | |  | |
| *Soil Depth* | *Shallow* | | | *Deep* | | |
|  | 90.91% | | | 9.09% | | |
|  |  | |  | |  | |
| *Root Type* | *Rhizomes* | | *Tap* | | *Both* | |
|  | 22.73% | | 4.55% | | 72.73% | |
|  |  | |  | |  | |
| *Soil Texture* | *Sand* | *Loam* | | *Clay* | | *Any* |
|  | 22.73% | 0% | | 0% | | 76.85% |
|  |  | |  | |  | |
| *Soil Fertility* | *Fertile* | | *Between* | | *Infertile* | |
|  | 9.09% | | 86.36% | | 4.55% | |
|  |  | |  | |  | |
| *Soil pH* | *Acid* | *Alkali* | | *Neutral* | | *Any* |
|  | 13.64% | 77.27% | | 9.09% | | 0% |
|  |  | |  | |  | |
| *Reproduction* | *Seed bank* | | *Vegetal Spread* | | *Both* | |
|  | 4.55% | | 4.52 | | 90.42% | |
|  |  | |  | |  | |
| *Reproductive Cycle* | *Annual* | | *Biannual* | | *Perennial* | |
|  | 22.73% | | 72.73% | | 4.55% | |

# SI.5. Masudpur VII

Table S12. Proportions of crop genera of crop assemblage at Masudpur VII (after Bates 2016, table 9.5). It should be noted that there were only three samples from the Late Harappan period, and that low sample number bias may have affected results.

| Crop Genera | Proportion of  Assemblage  *Early Harappan* | *Mature Harappan* | *Late Harappan* | Season |
| --- | --- | --- | --- | --- |
| *Hordeum vulgare* | 15.82% | 1.59% | 0.86% | W |
| *Triticum* sp. | 1.27% | 19.05% |  | W |
| *Hordeum/Triticum* | 4.43% | 28.57% | 7.76% | W |
| *Oryza* sp. | 1.27% |  | 6.03% | S |
| *Echinochloa* sp. | 18.35% | 12.60% | 6.03% | S |
| *Setaria* sp. | 6.96% |  |  | S |
| *Panicum* sp. | 4.43% |  | 0.86% | S |
| SEB | 5.06% | 3.17% |  | S |
| Indet. small millet | 7.60% | 4.76% | 10.34% | S |
| *Vigna* sp. | 0.63% | 3.17% | 4.31% | S |
| *Vigna radiata* |  | 3.17% |  | S |
| *Vigna mungo* |  |  | 1.72% | S |
| *Vigna radiata/mungo* | 0.63% | 1.59% |  | S |
| *Vigna acconitifolia* |  |  | 0.86% | S |
| *Macrotyloma* cf. *uniflorum* | 13.29% | 3.17% | 5.17% | S |
| *Pisum* sp. | 0.63% |  | 0.86% | W |
| *Cicer* sp. | 1.27% |  | 1.72% | W |
| *Vicia/Lathyrus* | 1.27% | 1.59% |  | W |
| Indet. Fabaceae | 4.43% | 3.17% | 5.17% | W/S/P |
| *Ziziphus mauritiana* | 5.70% | 6.35% | 2.59% | P |
| Indet. Fruit | 1.89% | 6.35% | 0.86% | P |
| *Brassica* sp. |  | 1.659% |  | W |
| *Coccinia* cf. *grandis* | 0.63% |  | 44.81% | S |
| Indet. Oilseed/Fibre | 4.43% |  |  | W/S/P |
| Summer crops | 58.87% | 31.75% | 80.17% |  |
| Winter crops | 24.68% | 52.38% | 11.20% |  |
| Tree/Orchard | 7.59% | 12.70% | 3.45% |  |
| Unknown | 8.86% | 3.17% | 5.17% |  |

Table S13. Average count per 10l sediment of weed genera at Masudpur VII in the Early Harappan period (after Bates 2016, table 7.8)

| Weed Taxa | Count per 10l | Season |
| --- | --- | --- |
| *Stellaria* sp. | 4.6 | S |
| *Eleocharis* sp. | 1.15 | S |
| *Fumaria* cf. *officinalis* | 0.2 | S |
| *Trianthema triquetra* | 0.15 | S |
| *Chrysopogon* sp. | 0.05 | S |
| Polygonaceae | 0.1 | W/S/P |
| Cyperaceae | 5.95 | W/S/P |
| Fabaceae | 0.45 | W/S/P |
| Indeterminate small grass | 1.05 | W/S/P |
| Solanum dulcamara | 0.1 | Tree |
| Round | 0.9 | W/S/P |
| Indet. seed | 1.65 | W/S/P |
| Summer weeds | 100% |  |
| Winter weeds | 0% |  |
| Both seasons weeds | 0% |  |

*Table S14. Relative proportion of summer* *weeds at Masudpur VII in the Early Harappan period by ecological preference indicators (after Bates 2016, table 10.3)*

| *Water* | *Wet* | | *Moist* | | *Dry* | |
| --- | --- | --- | --- | --- | --- | --- |
|  | 21.14% | | 78.86% | | 0% | |
|  |  | |  | |  | |
| *Flood Tolerance* | *Flood* | | *Drought* | | *Neither* | |
|  | 18.70% | | 4.07% | | 77.24% | |
|  |  | |  | |  | |
| *Soil Depth* | *Shallow* | | | *Deep* | | |
|  | 97.56% | | | 2.44% | | |
|  |  | |  | |  | |
| *Root Type* | *Rhizomes* | | *Tap* | | *Both* | |
|  | 19.51% | | 5.69% | | 74.80% | |
|  |  | |  | |  | |
| *Soil Texture* | *Sand* | *Loam* | | *Clay* | | *Any* |
|  | 3.25% | 0% | | 0% | | 96.75% |
|  |  | |  | |  | |
| *Soil Fertility* | *Fertile* | | *Between* | | *Infertile* | |
|  | 0% | | 96.75% | | 3.25% | |
|  |  | |  | |  | |
| *Soil pH* | *Acid* | *Alkali* | | *Neutral* | | *Any* |
|  | 0.81% | 80.49% | | 18.70% | | 0% |
|  |  | |  | |  | |
| *Reproduction* | *Seed bank* | | *Vegetal Spread* | | *Both* | |
|  | 5.69% | | 0.81% | | 93.50% | |
|  |  | |  | |  | |
| *Reproductive Cycle* | *Annual* | | *Biannual* | | *Perennial* | |
|  | 5.69% | | 93.50% | | 0.81% | |

Table S15. Average count per 10l sediment of weed genera at Masudpur VII in the Mature Harappan (after Bates 2016, table 7.8)

| Weed Taxa | Count per 10l | Season |
| --- | --- | --- |
| *Stellaria* sp. | 0.21 | S |
| *Eleocharis* sp. | 0.13 | S |
| *Chrysopogon* sp. | 0.04 | S |
| Cyperaceae | 1.75 | W/S/P |
| Indeterminate small grass | 0.21 | W/S/P |
| Round | 0.63 | W/S/P |
| Indet. seed | 0.13 | W/S/P |
| Summer weeds | 100% |  |
| Winter weeds | 0% |  |
| Both seasons weeds | 0% |  |

*Table S16. Percentages of summer* *weeds from the Mature Harappan period at Masudpur VII by ecological preference indicators (after Bates 2016, table 10.4)*

| *Water* | *Wet* | | *Moist* | | *Dry* | |
| --- | --- | --- | --- | --- | --- | --- |
|  | 33.33% | | 66.67% | | 0% | |
|  |  | |  | |  | |
| *Flood Tolerance* | *Flood* | | *Drought* | | *Neither* | |
|  | 33.33% | | 11.11% | | 88.89% | |
|  |  | |  | |  | |
| *Soil Depth* | *Shallow* | | | *Deep* | | |
|  | 100% | | | 0% | | |
|  |  | |  | |  | |
| *Root Type* | *Rhizomes* | | *Tap* | | *Both* | |
|  | 44.44% | | 0% | | 55.56% | |
|  |  | |  | |  | |
| *Soil Texture* | *Sand* | *Loam* | | *Clay* | | *Any* |
|  | 11.11% | 0% | | 0% | | 88.89% |
|  |  | |  | |  | |
| *Soil Fertility* | *Fertile* | | *Between* | | *Infertile* | |
|  | 0% | | 100% | | 0% | |
|  |  | |  | |  | |
| *Soil pH* | *Acid* | *Alkali* | | *Neutral* | | *Any* |
|  | 11.11% | 55.56% | | 33.33% | | 0% |
|  |  | |  | |  | |
| *Reproduction* | *Seed bank* | | *Vegetal Spread* | | *Both* | |
|  | 0% | | 11.11% | | 88.89% | |
|  |  | |  | |  | |
| *Reproductive Cycle* | *Annual* | | *Biannual* | | *Perennial* | |
|  | 0% | | 88.89% | | 11.11% | |

Table S17. Average count per 10l sediment of weed genera at Masudpur VII in the Late Harappan (after Bates 2016, table 7.8). It should be noted that there were only three samples, and that low sample number bias may have affected results.

| Weed Taxa | Count per 10l | Season |
| --- | --- | --- |
| *Stellaria* sp. | 1.5 | S |
| *Eleocharis* sp. | 0.33 | S |
| cf. *Papaver rhoeas* | 0.17 | S |
| *Pennisetum* sp. | 0.17 | S |
| *Chrysopogon* sp. | 0.05 | S |
| Indet. Big Millet | 0.17 | S |
| Cyperaceae | 3.5 | W/S/P |
| Fabaceae | 0.5 | W/S/P |
| Indeterminate small grass | 0.33 | W/S/P |
| Round | 3.33 | W/S/P |
| Indet. seed | 0.5 | W/S/P |
| Summer weeds | 100% |  |
| Winter weeds | 0% |  |
| Both seasons weeds | 0% |  |

*Table S18. Relative proportion of summer* *weeds in the Late Harappan at Masudpur VII by ecological preference indicators (after Bates 2016, table 10.5). It should be noted that there were only three samples, and that low sample number bias may have affected results.*

| *Water* | *Wet* | | *Moist* | | *Dry* | |
| --- | --- | --- | --- | --- | --- | --- |
|  | 15.38% | | 76.92% | | 7.69% | |
|  |  | |  | |  | |
| *Flood Tolerance* | *Flood* | | *Drought* | | *Neither* | |
|  | 15.38% | | 7.69% | | 76.92% | |
|  |  | |  | |  | |
| *Soil Depth* | *Shallow* | | | *Deep* | | |
|  | 92.31% | | | 7.69% | | |
|  |  | |  | |  | |
| *Root Type* | *Rhizomes* | | *Tap* | | *Both* | |
|  | 23.08% | | 7.69% | | 69.23% | |
|  |  | |  | |  | |
| *Soil Texture* | *Sand* | *Loam* | | *Clay* | | *Any* |
|  | 15.38% | 0% | | 0% | | 84.62% |
|  |  | |  | |  | |
| *Soil Fertility* | *Fertile* | | *Between* | | *Infertile* | |
|  | 7.69% | | 92.31% | | 0% | |
|  |  | |  | |  | |
| *Soil pH* | *Acid* | *Alkali* | | *Neutral* | | *Any* |
|  | 7.69% | 69.23% | | 15.38% | | 7.69% |
|  |  | |  | |  | |
| *Reproduction* | *Seed bank* | | *Vegetal Spread* | | *Both* | |
|  | 7.69% | | 0% | | 92.31% | |
|  |  | |  | |  | |
| *Reproductive Cycle* | *Annual* | | *Biannual* | | *Perennial* | |
|  | 15.38% | | 84.62% | | 0% | |

# SI.6. Masudpur I

Table S19. Proportion of crop genera of crop assemblage at Masudpur I (after Bates 2016, table 9.7)

| Crop Genera | Proportion of  Assemblage | Season |
| --- | --- | --- |
| *Hordeum vulgare* | 17.75% | W |
| *Triticum* sp. | 1.65% | W |
| *Hordeum/Triticum* | 8.59% | W |
| *Oryza* sp. | 19.34% | S |
| *Echinochloa* sp. | 11.55% | S |
| *Setaria* sp. | 15.48% | S |
| *Panicum* sp. | 3.49% | S |
| SEB | 3.62% | S |
| Indet. small millet | 7.77% | S |
| *Vigna* sp. | 1.43% | S |
| *Vigna radiata* | 0.58% | S |
| *Vigna mungo* | 0.13% | S |
| *Vigna radiata/mungo* | 0.02% | S |
| *Vigna acconitifolia* | 0.30% | S |
| *Vigna trilobata* | 0.16% | S |
| *Macrotyloma* cf. *uniflorum* | 1.36% | S |
| *Pisum* sp. | 0.10% | W |
| *Cicer* sp. | 0.26% | W |
| *Lens* cf. *culinaris* | 0.14% | W |
| *Lathyrus* sp. | 0.06% | W |
| *Vicia/Lathyrus* | 0.04% | W |
| Indet. Fabaceae | 1.21% | W/S/P |
| *Ziziphus mauritiana* | 0.51% | P |
| Indet. fruit | 0.09% | P |
| *Brassica* sp. | 0.09% | W |
| *Sesamum* sp. | 0.48% | S |
| *Coccinia* cf. *grandis* | 0.34% | S |
| *Indigofera* sp. | 0.07% | S |
| *Linum* sp. | 0.19% | W |
| Indet. Oilseed/Fibre | 3.18% | W/S/P |
| Summer crops | 66.12% |  |
| Winter crops | 28.87% |  |
| Tree/Orchard | 0.60% |  |
| Unknown | 4.39% |  |

Table S20. Average count per 10l of sediment of weed genera at Masudpur I (after Bates 2016, table 7.10)

| Weed Taxa | Proportion of  assemblage | Season |
| --- | --- | --- |
| *Stellaria* sp. | 0.06 | S |
| *Eleocharis* sp. | 0.34 | S |
| *Fumaria* cf. *officinalis* | 0.16 | S |
| *Trianthema triquetra* | 0.27 | S |
| *Eragrostis* sp. | 7.83 | S |
| *Aeluropus* sp. | 0.14 | S |
| *Chrysopogon* sp. | 0.73 | S |
| *Brachiaria* sp. | 0.05 | S |
| Indet. big millet | 0.03 | S |
| *Medicago/Melilotus/Trifolium* | 0.99 | W |
| *Avena* sp. | 0.07 | W |
| *Chenopodium album* | 0.05 | W/S |
| *Solanum* cf. *dulcamara* | 0.06 | (Tree/shrub)) |
| Mimosoideae | 0.16 | (Tree) |
| *Acacia* sp. | 0.12 | (Tree) |
| Cyperaceae | 4.82 | W/S/P |
| Polygonaceae | 0.07 | W/S/P |
| Fabaceae | 0.06 | W/S/P |
| Indet. small grass | 2.85 | W/S/P |
| Small cereal | 1.49 | W/S/P |
| Round | 122.98 | W/S/P |
| Indet. curled embryo | 0.31 | W/S/P |
| Indet. seed | 4.35 | W/S/P |
| Summer weeds | 89.72% |  |
| Winter weeds | 9.44%% |  |
| Both seasons weeds | 0.46% |  |

*Table S21. Relative proportion of winter weeds at Masudpur I by ecological preference indicators (after Bates 2016, table 10.6)*

| *Water* | *Wet* | | *Moist* | | *Dry* | |
| --- | --- | --- | --- | --- | --- | --- |
|  | 0% | | 93.78% | | 6.22% | |
|  |  | |  | |  | |
| *Flood Tolerance* | *Flood* | | *Drought* | | *Neither* | |
|  | 0% | | 93.78% | | 6.22% | |
|  |  | |  | |  | |
| *Soil Depth* | *Shallow* | | *Deep* | | *Unknown* | |
|  | 6.22% | | 4.67% | | 89.11% | |
|  |  | |  | |  | |
| *Root Type* | *Rhizomes* | | *Tap* | | *Both* | |
|  | 6.22% | | 93.60% | | 0% | |
|  |  | |  | |  | |
| *Soil Texture* | *Sand* | *Loam* | | *Clay* | | *Any* |
|  | 0% | 0% | | 0% | | 100% |
|  |  | |  | |  | |
| *Soil Fertility* | *Fertile* | | *Between* | | *Infertile* | |
|  | 4.67% | | 95.33% | | 0% | |
|  |  | |  | |  | |
| *Soil pH* | *Acid* | *Alkali* | | *Neutral* | | *Any* |
|  | 6.22% | 89.11% | | 0% | | 4.67% |
|  |  | |  | |  | |
| *Reproduction* | *Seed bank* | | *Vegetal Spread* | | *Both* | |
|  | 100% | | 0% | | 0% | |
|  |  | |  | |  | |
| *Reproductive Cycle* | *Annual* | | *Biannual* | | *Perennial* | |
|  | 4.67% | | 95.33% | | 0% | |

*Table S22. Relative proportion of summer weeds at Masudpur I by ecological preference indicators (after Bates 2016, table 10.7)*

| *Water* | *Wet* | | *Moist* | | *Dry* | |
| --- | --- | --- | --- | --- | --- | --- |
|  | 6.40% | | 12.93% | | 80.67% | |
|  |  | |  | |  | |
| *Flood Tolerance* | *Flood* | | *Drought* | | *Neither* | |
|  | 4.22% | | 11.74% | | 84.04% | |
|  |  | |  | |  | |
| *Soil Depth* | *Shallow* | | | *Deep* | | |
|  | 96.54% | | | 3.46% | | |
|  |  | |  | |  | |
| *Root Type* | *Rhizomes* | | *Tap* | | *Both* | |
|  | 94.23% | | 5.15% | | 0.62% | |
|  |  | |  | |  | |
| *Soil Texture* | *Sand* | *Loam* | | *Clay* | | *Any* |
|  | 12.85% | 0% | | 0% | | 87.15% |
|  |  | |  | |  | |
| *Soil Fertility* | *Fertile* | | *Between* | | *Infertile* | |
|  | 1.29% | | 97.02% | | 1.69% | |
|  |  | |  | |  | |
| *Soil pH* | *Acid* | *Alkali* | | *Neutral* | | *Any* |
|  | 7.72% | 87.15% | | 4.60% | | 0.53% |
|  |  | |  | |  | |
| *Reproduction* | *Seed bank* | | *Vegetal Spread* | | *Both* | |
|  | 85.63% | | 9.15% | | 5.21% | |
|  |  | |  | |  | |
| *Reproductive Cycle* | *Annual* | | *Biannual* | | *Perennial* | |
|  | 86.77% | | 4.26% | | 8.97% | |

# SI.7. Bahola

Table S23. Proportion of crop genera of crop assemblage at Bahola in the Late Harappan and PGW periods (after Bates 2016, table 9.9)

| Crop Genera | Proportion of  Assemblage  *Late Harappan* | *PGW* | Season |
| --- | --- | --- | --- |
| *Hordeum vulgare* | 0.68% | 2.47% | W |
| *Triticum* sp. |  | 1.28% | W |
| *Hordeum/Triticum* | 6.20% | 10.71% | W |
| *Oryza* sp. | 17.35% | 9.10% | S |
| *Echinochloa* sp. | 25.82% | 16.27% | S |
| *Setaria* sp. | 14.83% | 9.33% | S |
| *Panicum* sp. | 0.68% | 4.68% | S |
| SEB | 1.88% | 4.37% | S |
| Indet. small millet | 17.20% | 25.18% | S |
| *Vigna* sp. | 4.99% | 0.77% | S |
| *Vigna radiata* | 0.92% | 1.55% | S |
| *Vigna mungo* | 0.22% |  | S |
| *Vigna radiata/mungo* | 0.11% |  | S |
| *Vigna trilobata* | 0.33% |  | S |
| *Macrotyloma* cf. *uniflorum* | 3.66% | 0.77% | S |
| *Lens* cf. *culinaris* |  | 1.55% | W |
| Indet. Fabaceae | 4.43% | 4.17% | W/S/P |
| *Ziziphus mauritiana* | 0.33% | 4.81% | P |
| Indeterminate Fruit | 0.13% |  | P |
| *Brassica* sp. |  | 1.28% | W |
| *Coccinia* cf. *grandis* | 0.22% |  | S |
| *Indigofera* sp. |  | 0.85% | S |
| Indet. Oilseed/Fibre |  | 0.85% | W/S/P |
| Summer crops | 88.24% | 72.88%% |  |
| Winter crops | 6.87% | 17.29% |  |
| Tree/Orchard | 0.46% | 4.81% |  |
| Unknown | 4.43% | 5.02% |  |

Table S24. Average count per 10l of sediment of weed genera at Bahola in the Late Harappan (after Bates 2016, table 7.12)

| Weed Taxa | Count per 10l | Season |
| --- | --- | --- |
| *Stellaria* sp. | 0.02 | S |
| *Eleocharis* sp. | 1.13 | S |
| *Eragrostis* sp. | 0.20 | S |
| *Chrysopogon* sp. | 5.16 | S |
| *Echinochloa crus-galli* | 0.02 | S |
| *Rumex* sp. | 0.02 | W |
| *Cheopodium album* | 1.58 | W/S |
| Cyperaceae | 11.15 | W/S/P |
| Fabaceae | 0.62 | W/S/P |
| Indet. small grass | 2.16 | W/S/P |
| Indet. curled embryo | 0.02 | W/S/P |
| Round | 0.92 | W/S/P |
| Indet. seed | 2.48 | W/S/P |
| Summer weeds | 80.39% |  |
| Winter weeds | 0.25% |  |
| Both seasons weeds | 19.36% |  |

*Table S25. Relative proportion of winter weeds at Late Harappan period Bahola by ecological preference indicators (after Bates 2016, table 10.8)*

| *Water* | *Wet* | | *Moist* | | *Dry* | |
| --- | --- | --- | --- | --- | --- | --- |
|  | 1.05% | | 98.95% | | 0% | |
|  |  | |  | |  | |
| *Flood Tolerance* | *Flood* | | *Drought* | | *Neither* | |
|  | 0% | | 98.95% | | 1.05% | |
|  |  | |  | |  | |
| *Soil Depth* | *Shallow* | | | *Deep* | | |
|  | 0% | | | 100% | | |
|  |  | |  | |  | |
| *Root Type* | *Rhizomes* | | *Tap* | | *Both* | |
|  | 0% | | 100% | | 0% | |
|  |  | |  | |  | |
| *Soil Texture* | *Sand* | *Loam* | | *Clay* | | *Any* |
|  | 0% | 0% | | 1.05% | | 98.95% |
|  |  | |  | |  | |
| *Soil Fertility* | *Fertile* | | *Between* | | *Infertile* | |
|  | 98.95% | | 1.05% | | 0% | |
|  |  | |  | |  | |
| *Soil pH* | *Acid* | *Alkali* | | *Neutral* | | *Any* |
|  | 98.95% | 0% | | 1.05% | | 0% |
|  |  | |  | |  | |
| *Reproduction* | *Seed bank* | | *Vegetal Spread* | | *Both* | |
|  | 98.95% | | 1.05% | | 0% | |
|  |  | |  | |  | |
| *Reproductive Cycle* | *Annual* | | *Biannual* | | *Perennial* | |
|  | 98.95% | | 0% | | 1.05% | |

*Table S26. Relative proportion of summer weeds in the Late Harappan at Bahola by ecological preference indicators (after Bates 2016, table 10.9)*

| *Water* | *Wet* | | *Moist* | | *Dry* | |
| --- | --- | --- | --- | --- | --- | --- |
|  | 14.31% | | 83.18% | | 2.51% | |
|  |  | |  | |  | |
| *Flood Tolerance* | *Flood* | | *Drought* | | *Neither* | |
|  | 14.14% | | 82.73% | | 3.13% | |
|  |  | |  | |  | |
| *Soil Depth* | *Shallow* | | | *Deep* | | |
|  | 80.23% | | | 19.77% | | |
|  |  | |  | |  | |
| *Root Type* | *Rhizomes* | | *Tap* | | *Both* | |
|  | 80.02% | | 19.77% | | 0.21% | |
|  |  | |  | |  | |
| *Soil Texture* | *Sand* | *Loam* | | *Clay* | | *Any* |
|  | 64.01% | 0% | | 0% | | 35.99% |
|  |  | |  | |  | |
| *Soil Fertility* | *Fertile* | | *Between* | | *Infertile* | |
|  | 19.60% | | 80.40% | | 0% | |
|  |  | |  | |  | |
| *Soil pH* | *Acid* | *Alkali* | | *Neutral* | | *Any* |
|  | 63.37% | 3.13% | | 14.14% | | 19.36% |
|  |  | |  | |  | |
| *Reproduction* | *Seed bank* | | *Vegetal Spread* | | *Both* | |
|  | 22.28% | | 63.37% | | 14.35% | |
|  |  | |  | |  | |
| *Reproductive Cycle* | *Annual* | | *Biannual* | | *Perennial* | |
|  | 22.52% | | 14.12% | | 63.37% | |

Table S27. Average count per 10l of sediment of weed genera at Bahola in the PGW period (after Bates 2016, table 7.12)

| Weed Taxa | Count per 10l | Season |
| --- | --- | --- |
| *Stellaria* sp. | 0.07 | S |
| *Eleocharis* sp. | 0.18 | S |
| *Eragrostis* sp. | 0.31 | S |
| *Chrysopogon* sp. | 0.06 | S |
| *Paspalum* sp. | 0.03 | S |
| *Cheopodium album* | 0.03 | W/S |
| Cyperaceae | 1.78 | W/S/P |
| Polygonaceae | 0.03 | W/S/P |
| Fabaceae | 0.15 | W/S/P |
| Indet. small grass | 0.75 | W/S/P |
| Round | 0.07 | W/S/P |
| Indet. seed | 0.98 | W/S/P |
| Summer weeds | 95.59% |  |
| Winter weeds | 0% |  |
| Both seasons weeds | 4.41% |  |

*Table S28. Relative proportion of summer weeds at PGW period Bahola by ecological preference indicators (after Bates 2016, table 10.10)*

| *Water* | *Wet* | | *Moist* | | *Dry* | |
| --- | --- | --- | --- | --- | --- | --- |
|  | 30.35% | | 23.74% | | 45.91% | |
|  |  | |  | |  | |
| *Flood Tolerance* | *Flood* | | *Drought* | | *Neither* | |
|  | 30.35% | | 13.89% | | 55.77% | |
|  |  | |  | |  | |
| *Soil Depth* | *Shallow* | | | *Deep* | | |
|  | 95.07% | | | 4.93% | | |
|  |  | |  | |  | |
| *Root Type* | *Rhizomes* | | *Tap* | | *Both* | |
|  | 85.22% | | 4.93% | | 9.85% | |
|  |  | |  | |  | |
| *Soil Texture* | *Sand* | *Loam* | | *Clay* | | *Any* |
|  | 8.96% | 0% | | 0% | | 91.04% |
|  |  | |  | |  | |
| *Soil Fertility* | *Fertile* | | *Between* | | *Infertile* | |
|  | 8.62% | | 91.31% | | 0% | |
|  |  | |  | |  | |
| *Soil pH* | *Acid* | *Alkali* | | *Neutral* | | *Any* |
|  | 12.65% | 55.77% | | 26.65% | | 4.93% |
|  |  | |  | |  | |
| *Reproduction* | *Seed bank* | | *Vegetal Spread* | | *Both* | |
|  | 50.84% | | 12.65% | | 36.51% | |
|  |  | |  | |  | |
| *Reproductive Cycle* | *Annual* | | *Biannual* | | *Perennial* | |
|  | 50.84% | | 40.20% | | 8.96% | |

# Additional Bibliography

Bates, J. (2016) Social organisation and change in Bronze Age South Asia: a mutli-proxy approach to urbanisation, deurbanisation and village life through phytolith and macrobotanical analysis (PhD). University of Cambridge, Cambridge.

Bhatt, M.D., Singh, S.P. (2007) Soil seed bank dynamics of weed flora in upland and lowland paddy cultivation areas of far western Nepal. *Scientific World* 5, 54–59.

Boutton, T.W., Harrison, A.T., Smith, B.N. (1980) Distribution of biomass of species differing in photosynthetic pathways along an altitudinal transect in southeastern Wyoming grassland. *Oecologica* 45, 287–298.

Bruhl, J.J., Wilson, K.L. (2007) Towards a comprehensive survey of C3 and C4 photosynthetic pathways in Cyperaceae. *Aliso: a journal of systematic and evolution botany* 23, 99–148.

Caton, B.P., Mortimer, M., Hill, J.E., Johnson, D.E. (2010) *A practical field guide to weeds of rice in Asia, 2nd edition*. Los Banos: International Rice Research Institute.

De Wet, J.M.J., Prasada Rao, K.E., Mengesha, M.H., Brink, D.E. (1983a) Domestication of sawa millet (*Echinochloa colona*). *Economic Botany* 37, 283–291.

De Wet, J.M.J., Prasada Rao, K.E., Brink, D.E. (1983b) Systematics and domestication of *Panicum sumatrense* (Graminae). *Journal d’Agriculture Traditionelle et de Botanique Appliquee* 30, 159–168.

Fuller, D.Q. (2000) The emergence of agricultural societies in south India: botanical and archaeological perspectives (PhD). University of Cambridge, Cambridge.

Fuller, D.Q., Sato, Y.-I., Castillo, C., Qin, L., Weisskopf, A., Kingwell-Banham, E., Song, J., Ahn, S.-M., van Etten, J. (2011) Consilience of genetics and archaeobotany in the entangled history of rice. *Archaeological and Anthropological Sciences* 2, 115–131.

Galinato, M.I., Moody, K., Piggin, C.M. (1999) *Upland rice weeds of south and southeast Asia*. Kyoto: Int. Rice Res. Inst.

García-Palacios, P., Querejeta, J.I., Maestre, F.T., Escudero, A., Valladares, F. (2011) Impact of simulated changes in rainfall regime and nutrient deposition on the relative dominance and isotopic composition of ruderal plants in anthropogenic grasslands. *Plant and Soil* 352, 303–319.

Gonçalves, M.L. (1978) *Flora Zambesiaca*. London.

Gulzar, S., Khan, M.A. (2001) Seed germination of a halophytic grass *Aeluropus lagopoides*. *Annals of Botany* 87, 319–324.

Gupta, P.C., Rao, C.V. (2012) Morpho-anatomical and physicochemical studies of *Fumaria indica* (Hausskn.) Pugsley. *Asian Pacific Journal of Tropical Biomedicine* 2, 830–834.

Harlan, H.V. & Martini, M.L. (1936) Problems and results in barley breeding. In *Yearbook of Agriculture 1936*. Washington D.C.: U.S. Department of Agriculture.

Holm, L.G., Plucknett, D., Pancho, J., Herberger, J. (1977) *World weeds: natural histories and distribution*. Honolulu:University of Hawaii Press.

Hooker, J.D. (1875) *Flora of British India. I.* London: Reeve & Co.

Johnston, M.B., Olivares, A.E., Calderon, C.E. (2009) Effect of quantity and distribution of rainfalls on *Hordeum murinum* L. growth and development. *Chilean Journal of Agricultural Research* 69, 188–197.

McNaughton, I.H., Harper, J.L. (1964) *Papaver* L. *Journal of Ecology* 52, 767–793.

Miles, J.W., Maass, B.L., Valle, C.B. (1996) *Brachiaria: biology, agronomy and improvement*. Columbia: CIAT.

Rundell, P.W. (1980) The ecological distribution of C4 and C3 grasses in Hawaiian Islands. *Oecologica* 45, 354–359.

Saraswat, K.S. (1993) Plant economy of Late Harappan at Hulas. *Puratattva* 23, 1–12.

Sen, D.N. (1981) *Ecological approaches to Indian weeds*. Jodhpur: Geobios International.

Shetty, B.V., Singh, V. (1987) *Flora of Rajasthan*. Calcutta: Botanical Survey of India.

Sikolia, S., Beck, E., Onyango, J.C. (2009) Carbon dioxide compensation points of some dicots of the centrospermeae species and their ecological implications for agroforestry. *International Journal of Botany* 5, 67–75.

Stevens, C.J. (1996) Iron Age and Roman agriculture in the Upper Thames Valley: archaeobotanical and social perspectives (PhD). University of Cambridge, Cambridge.

Tanveer, A., Mumtaz, K., Javaid, M.N., Chaudhry, M.N., Balal, R.M., Kaliq, A. (2013) Effect of ecological factors on germination of horse purslane (*Trianthema portulacastrum*). *Planta Daninha, Viçose-MĢ* 31, 587–597.

Waghmode, A.P. (1979) Kranz leaf anatomy & C4 dicarboxylic acid pathway of photosynthesis in *Aeluropus lagopoides* L. *Indian Journal of Experimental Biology* 17, 606–7.

Waller, S.S., Lewis, J.K. (1979) Occurrence of C3 and C4 photosynthetic pathways in North American grasses. *Journal of Range Management* 32, 12–28.

Weisskopf, A., Harvey, E., Kingwell-Banham, E., Kajale, M., Mohanty, R., Fuller, D.Q. (2014) Archaeobotanical implications of phytolith assemblages from cultivated rice systems, wild rice stands and macro-regional patterns. *Journal of Archaeological Science* 51, 43–53.

*Online references*

Agriculture-Aajtak (2016) [Medicago] http://www.agriculture-aajtak.blogspot.co.uk/2012/10/indian-crops-and-their-classification.html. Accessed 11^th^ March 2016 14.02

Anbg (2016) [Fumaria] http://www.anbg.gov.au/abrs/online-resources/flora/redirect.jsp. Accessed 7^th^ March 2016 17.06

Archive (2016) [Hordeum] http://archive.agric.wa.gov.au/objtwr/imported_assets/content/pw/weed/major/barleyg.pdf. Accessed 11^th^ March 2016 16.34.

Arkive (2016) [Aeluropus] http://www.arkive.org/aeluropus/aeluropus-lagopoides/. Accessed 10^th^ March 2016 18.08.

Botanicgardens (2016) [Trianthema] http://www.botanicgardens.gov.lk/herbarium/index.php?option=com_sobi2&catid=124&Itemid=90. Accessed 7^th^ March 2016 15.28

CAES (2016) [Pennisetum] http://www.caes.uga.edu/publications/pubDetail.cfm?pk_id=7172. Accessed 7^th^ March 2016 15.34

ECOCROP (2016) [Avena] http://ecocrop.fao.org/ecocrop/srv/en/dataSheet?id=3569. Accessed 7^th^ March 201615.53.

ECOCROP (2016) [Brachiaria] http://ecocrop.fao.org/ecocrop/srv/en/cropView?id=3814. Accessed 11^th^ March 2016 11.01.

ECOCROP (2016) [Chrysopogon] http://ecocrop.fao.org/ecocrop/srv/en/dataSheet?id=690. Accessed 7^th^ March 2016 12.30.

ECOCROP (2016) [Echinochloa] http://ecocrop.fao.org/ecocrop/srv/en/dataSheet?id=970. Accessed 9^th^ March 2016 15.08.

ECOCROP (2016) [Eragrostis] http://ecocrop.fao.org/ecocrop/srv/en/dataSheet?id=5746. Accessed 10^th^ March 2016 18.10.

ECOCROP (2016) [FindForm] http://ecocrop.fao.org/ecocrop/srv/en/cropFindForm. Accessed 15^th^ February 2016

ECOFLORA (2016) [Fumaria] http://www.ecoflora.co.uk/search_ecochars.php?plant_no=660120330%20&cs=1. Accessed 11^th^ March 2016 14.55.

ECOCROP (2016) [Pennisetum] http://ecocrop.fao.org/ecocrop/srv/en/dataSheet?id=8418. Accessed 7^th^ March 2016 15.18.

ECOCROP (2016) [Paspalum] http://ecocrop.fao.org/ecocrop/srv/en/dataSheet?id=1630. Accessed 10^th^ March 2016 18.47.

ECOFLORA (2016) [Papaver] http://www.ecoflora.co.uk/search_ecochars.php?plant_no=660010020. Accessed 17^th^ March 2016 15.17

eFlora (2016) [Fumaria] https://sites.google.com/site/efloraofindia/species/m---z/p/papaveraceae/fumaria/fumaria-indica. Accessed 7^th^ March 2016 16.37

FAO (2016) [Chrysopogon] http://www.fao.org/ag/agp/AGPC/doc/gbase/data/pf000203.htm. Accessed 7^th^ March 2016 15.53.

FAO (2016) [Coix] http://www.fao.org/ag/agp/agpc/doc/gbase/data/pf000205.htm. Accessed 11^th^ March 2014 15.08.

FAO (2016) [Pennisetum] http://www.fao.org/ag/AGP/AGPC/doc/GBASE/data/pf000297.htm. Accessed 7^th^ March 201615.11.

Fed (2016) [Melilotus] http://www.fs.fed.us/database/feis/plants/forb/melspp/all.html. Accessed 11^th^ March 2016 12.35.

Fed (2016) [Solanum] http://www.fs.fed.us/database/feis/plants/shrub/soldul/all.html#BOTANICAL%20AD%20ECOLOGICAL%20CHARACTERISTICS. Accessed 8^th^ March 2016 16.57.

FlowersofIndia (2016) [Fumaria] http://www.flowersofindia.net/catalog/slides/Indian%20Fumitory.html. Accessed 7^th^ March 2016 16.30

Homeguides (2016) [Papaver] http://homeguides.sfgate.com/identify-papaver-rhoeas-l 75484.html. Accessed 7^th^ March 2016 14.15.

Ibaf (2016) [Medicago] http://www.ibaf.cnr.it/phyto/schede/MEDICAGOLUPULINA-diesel.pdf. Accessed 11^th^ March 2016 9.58.

Icunredlist (2016) [Brachiaria] http://www.icunredlist.org/details/164167/0. Accessed 11^th^ March 2016.

Idao (2016) [Brachiaria] http://www.idao.cirad.fr/content/oscar/especes/b/brare/brare.html. Accessed 11^th^ March 2016 11.15

Illinoiswildflowers (2016) [Stellaria] http://www.illinoiswildflowers.info/weeds/plants/gr_chickweed.htm. Accessed 11^th^ March 2016 12.44

Indiabiodiversity (2016) [Chrysopogon] http://indiabiodiversity.org/species/show/229164. Accessed 7^th^ March 2016 15.57.

Indiabiodiversity (2016) [Trianthema] http://indiabiodiversity.org/species/show/249742. Accessed 6^th^ March 2016 15.33

Irrd (2016) [Medicago] http://www.Irrd.org/Irrd17/2/youn17018.htm 11/3/14 11.39. Accessed 11th March 2016 11.39.

JStor (2016) [Chrysopogon] http://plants.jstor.org/upwta/2_426. Accessed 7th March 2016 16.00

Kew (2016) [Aeluropus] http://www.kew.org/data/grasses-db/www/imp00061.htm. Accessed 10th March 2016 18.10.

Kew (2016) [Papaver] http://www.kew.org/plants-fungi/Papaver-rhoeas.htm. Accessed 10^th^ March 2016 15.57

Murrumbidgee (2016) [Fumitory] http://www.murrumbidgee.cma.nsw.gov.au/downloads/info_sheets/dryland_cropping Fumitory_Revised.pdf. Accessed 7^th^ March 2016 17.04

Murrumbidgee (2016) [Fumitory_revised] http://www.murrumbidgee.cma.nsw.gov.au/downloads/info_sheets/dryland_cropping Fumitory_Revised.pdf. Accessed 11^th^ March 2016 14.45.

Pfaf (2016) [Coix] http://www.pfaf.org/user/Plant.aspx?LatinName=Coix+lacryma-jobi. Accessed 7^th^ March 2016 15.57.

Pfaf (2016) [Eleocharis] http://www.pfaf.org/user/Plant.aspx?LatinName=Eleocharis. Accessed 11^th^ March 2016 9.58.

Pfaf (2016) [Medicago] http://www.pfaf.org/user/Plant.aspx?LatinName=Medicago+Lupulina. Accessed 11^th^ March 2016 9.51.

Pfaf (2016) [Papaver] http://www.pfaf.org/user/Plant.aspx?LatinName=Papaver+rhoeas. Accessed 7^th^ March 2016 13.00.

Pfaf (2016) [Stellaria] http://www.pfaf.org/user/plant.aspx?LatinName=Stellaria+media. Accessed 11^th^ March 2016 9.53.

Plantnet (2016) [Trianthema] http://plantnet.rbgsyd.nsw.gov.au/cgibin/NSWfl.pl?page=nswfl&lvl=sp&name=Trianthema~triquetra. Accessed 7^th^ March 2016 15.20

Proseanet (2016) [Coix] http://proseanet.org/prosea/e-prosea_detail.php?frt=&id=57. Accessed 7^th^ March 2016 15.53.

Prota (2016) [Coix] http://database.prota.org/PROTAhtml/Coix%20lacryma-jobi_En.htm. Accessed 11^th^ March 2106 15.05.

PSU (2016) [Stellaria] http://www.extension.psu.edu/pests/weeds/weed-id/commonchickweed. Accessed 11^th^ March 2016 12.47

RHS (2016) [Papaver] http://apps.rhs.org.uk/plantselector/plant?plantid=1378. Accessed 7^th^ March 2016 13.03

Tropicos (2016) [Aeluropus] http://www.tropicos.org/Name/25532114. Accessed 10^th^ March 2016 18.10.

Tropicos (2016) [Brachiaria] http://www.tropicos.org/Name/40030331?projectid=32. Accessed 11^th^ March 2016 11.11.

Tropicos (2016) [Chenopodium] http://www.tropicos.org/Name/7200019?projectid=32. Accessed 11^th^ March 2016 9.44

Tropicos (2016) [Cyperaceae] http://www.tropicos.org/Name/42000356?projectid=32. Accessed 10^th^ March 2016 12.10.

Tropicos (2016) [Echinochloa] http://www.tropicos.org/Name/25529345?projectid=32. Accessed 9^th^ March 2016 15.15.

Tropicos (2016) [Eleocharis] http://www.tropicos.org/Name/4000199. Accessed 11^th^ March 2016 9.53.

Tropicos (2016) [Eragrostis] http://www.tropicos.org/Name/40004497. Accessed 10^th^ March 2016 18.08.

Tropicos (2016) [Fabaceae] http://www.tropicos.org/Name/42000184?projectid=32. Accessed 10^th^ March 2016 12.05.

Tropicos (2016) [Fumaria] http://www.tropicos.org/Name/24000229?projectid=32. Accessed 11^th^ March 2016 14.55.

Tropicos (2016) [Hordeum] http://www.tropicos.org/Name/25511183?projectid=32. Accessed 11^th^ March 2016 8.58.

Tropicos (2016) [Polygonaceae] http://www.tropicos.org/Name/42000078?projectid=32. Accessed 10^th^ March 2016 12.02.

Tropicos (2016) [Rumex] http://www.tropicos.org/Name/40027658?projectid=32. Accessed 11^th^ March 2016 11.33.

Tropicos (2016) [Scirpus] http://www.tropicos.org/Name/40033372. Accessed 11^th^ March 2016 10.11.

Tropicos (2016) [Solanum] http://www.tropicos.org/Name/29600249. Accessed 8^th^ March 2016 17.00

Tropicos (2016) [Trianthema] http://www.tropicos.org/Name/700006?projectid=32. Accessed 11^th^ March 2016 15.00.

Ucanr (2016) [Medicago] http://www.ucanr.org/sites/asi/db/covercrops.cfm?crop_id=8. Accessed 11^th^ March 2016 9.53.

Ucipm (2016) [Stellaria] http://www.ucipm.udavis.edu/PGM/PESTNOTES/pn74129.html. Accessed 11^th^ March 2016 12.15.

USDA (2016) [Eleocharis] http://plants.usda.gov/core/profile?symbol=ELEOC. Accessed 11^th^ March 2016 10.02.

USDA (2016) [Eragrostis] http://plants.usda.gov/core/profile?symbol=ERAGR. Accessed 11^th^ March 2016 9.10.

Weedecology (2016) [Chenopodium] https://weedecology.css.cornell.edu/weed/weed.php?id=8. Accessed 11^th^ March 2016 9.34.

Wildseed (2016) [Papaver] http://wildseed.co.uk/species/view/8. Accessed 7th March 2016 14.26.

Wric (2016) [Hordeum] http://www.wric.ucdavis.edu/information/naturalareas/wr_H/Hprdeum_murinum_murinum.pdf. Accessed 11^th^ March 2016 16.23

1. Ley cropping – rotation of crops with pulses and/or pasture. [↑](#footnote-ref-1)
2. SEB – *Setaria/Echinochloa/Brachiaria*: a complex of small millets with long embryos which can be difficult to distinguish between if the grain is damaged (after Fuller 2000). [↑](#footnote-ref-2)
